# Enrichment of Persistent Organic Pollutants in Microplastics from Coastal Waters

**DOI:** 10.1021/acs.est.4c10835

**Published:** 2024-12-04

**Authors:** Lin-Chi Wang, Justin Chun-Te Lin, Jia-An Ye, Yee Cheng Lim, Chiu-Wen Chen, Cheng-Di Dong, Ta-Kang Liu

**Affiliations:** † Department of Marine Environmental Engineering, National Kaohsiung University of Science and Technology, Kaohsiung City 811213, Taiwan; ‡ Department of Environmental Engineering and Science, Feng Chia University, Taichung City 407102, Taiwan; § Institute of Ocean Technology and Marine Affairs, National Cheng Kung University, No. 1, University Road, Tainan City 701401, Taiwan

**Keywords:** persistent organic pollutants (POPs), enrichment factor, microplastics, concentrating effect

## Abstract

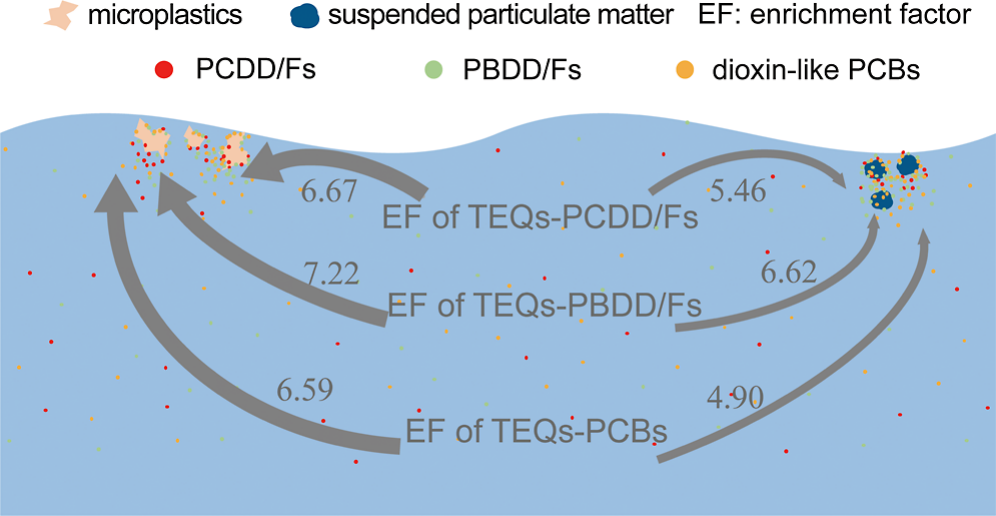

Despite the adsorption of microplastics (MPs), the precise quantification of their concentrating effect on persistent organic pollutants (POPs) remains uncertain. Therefore, in this study, POPs in MPs, POPs in suspended particulate matter (SPM), and dissolved POPs in seawater were distinguished to quantify the enrichment factor (EF) for characterizing the concentrating effects of MPs and SPM on POPs. The results showed that the logarithm of EF (log EF) for POPs in MPs was 5.94 to 7.14. For POPs, the concentrating effect of MPs was 1 to 2 orders of magnitude greater than that of SPM. Moreover, for PCDD/Fs, PBDD/Fs, and PBDEs, the concentrating effect of MPs was roughly comparable to that of organic matter in SPM, while it was 1 to 2 orders of magnitude higher than that of organic matter for dioxin-like PCBs and PBBs. The MPs were prone to sorbing highly toxic POP congeners. When the logarithm of the *n*-octanol–water partition coefficient (log *K*_OW_) of POP homologues ranged from 5.5 to 8.25, the log EF for POP homologues in MPs approximately was between 5 and 7. The heterogeneous MPs from the field environment affected their capacity to sorb POPs, causing a nonsignificant correlation between the enrichment factor and log *K*_OW_.

## Abstract 2

The concentrating effect of microplastics on POPs was higher than that of suspended particulate matter (SPM) in seawater, while it was roughly comparable to that of organic matter in SPM for PCDD/Fs, PBDD/Fs, and PBDEs.

## 1 Introduction

In the marine environment, large plastic wastes are broken into particles or fragments by natural forces such as waves, ultraviolet radiation, and physical abrasion.^1^ These particles or fragmented plastic materials are considered microplastics (MPs) when their size ranges from 1 μm to 5 mm.^2^ MPs are widely distributed in diverse marine environments from the equator to the poles through physical transport such as ocean currents, tides, and thermohaline circulation.^3^ Moreover, marine environmental conditions, such as salinity, temperature, pH, and physical forces, significantly affect the shape, surface area, and color of MPs.^2^ The MPs are classified into films, spheres, flakes, fibers, and irregular fragments according to their shape.^4^ Currently, the production, use, and disposal of plastic is clearly responsible for MPs in the global environmental system.^5^ In the marine environment, the temperature, oxygen, pH, chemicals, and salinity of seawater also involve the formation of biofilms, which affect the ability of MPs to adsorb heavy metals and organic pollutants.^6,7^

Marine MPs are easily ingested by organisms owing to their small size. Marine organisms such as cetaceans, fishes, echinoderms, and bivalves have been found to contain MPs in their bodies.^8−11^ However, MPs ingested by marine organisms can cause intestinal obstruction and negatively affect species growth and reproduction.^12^ Additionally, MPs are hydrophobic and have a higher specific surface area than large plastic wastes, which leads to their adsorption of a significant amount of chemicals.^2,6^ The chemicals adsorbed by MPs could be released in the digestive system of the organism and accumulate in the internal organs.^13^ As the quantity of MPs in the ocean increases, their total surface area also increases, leading to greater adsorption of hydrophobic chemicals.^14,15^ When MPs with toxic pollutants are ingested by organisms, the toxic pollutants are transferred to the body and bioaccumulate in the food chain.^16^ Therefore, marine organisms in the biosphere exposed to seawater enriched with MPs face the risk of toxic accumulation.^17^

Since the mid-20th century, the global environment has been gradually polluted by persistent organic pollutants (POPs). These POPs are widely found in coastal areas and aquatic sediments^18^ and have spread all over the global oceans including the Arctic and Antarctic waters.^19^ POPs could resist photodissociation and biochemical degradation to accumulate in the environment and are prone to bioaccumulation.^20^ Previous studies have shown that polybrominated diphenyl ethers (PBDEs) are already contained in the tissues of marine organisms, such as tuna, rockfish, and sea otters.^21−23^ In addition to accumulating in environmental matrices and organisms, different types of POPs such as PCDD/Fs, PBDD/Fs, PBDEs, PCBs, and PBBs could be sorbed by MPs.^18,24,25^ Moreover, MPs may desorb POPs in organisms and accumulate in biological tissues.^26^ Chua et al. (2014) indicated that PDBEs adsorbed by MPs were transferred into the tissue of marine amphipods.^27^ In terms of MPs adsorbing PCDD/Fs and PCBs, Abihssira-García et al. (2022) found that four types of MPs can adsorb Σ_6_ PCDDs and four PCB Aroclors from aquatic feeds.^24^ However, there is insufficient research data on the sorption of PBDD/Fs and PBBs by MPs, necessitating further investigation.^18^ In general, the sorption capacity of MPs depends on their polymeric properties (i.e., crystallinity, polarity, and functional groups) with POP content, while the physicochemical properties of the environment also control the sorption process.^28^ Eventually, the interaction of environmental pollutants and MPs may change their environmental behavior, toxicity, and bioavailability.^29^ Moreover, the partition coefficient of organic pollutants on MPs is closely related to the hydrophobicity.^15^ Wang et al. (2021) also found that the congeners of POPs with the higher *n*-octanol–water partition coefficient (*K*_ow_) were more likely to be adsorbed by MPs.^18^

Recently, the abundance, distribution, and fate of MPs in marine environments have been extensively studied, and studies regarding the sorption of toxic chemicals on MPs have gradually increased.^15,18,28,30^ However, literature on the interaction of MPs with PCDD/Fs, PBDD/Fs, PBDEs, PCBs, and PBBs is still limited.^18^ Despite the well-known adsorption capabilities of MPs, the precise quantification of their concentrating effect on POPs remains uncertain.^31,32^ Therefore, assessing the influence of MPs produced by humans on the distribution of POPs is challenging and still a field requiring further research. To fill the knowledge gap, this study aimed to characterize the concentrating effect of MPs on POPs by evaluating the enrichment factor. The enrichment factor describes the concentration ratio of POPs in MPs or POPs in SPM relative to dissolved POPs in seawater, respectively. The POPs identified in this study included 17 PCDD/Fs, 12 PBDD/Fs, 14 PBDEs, 12 dioxin-like PCBs, and 5 PBBs. More specifically, the objectives of this study were (1) to distinguish the concentrations of POPs in MPs, POPs in suspended particulate matter (SPM), and dissolved POPs in seawater, (2) to quantify the enrichment factors for POPs in MPs and POPs in SPM, and (3) to characterize the POP homologues and the concentrating effect of MPs and SPM in the marine environment.

## 2 Methods and Materials

### 2.1 Study Site and Sampling

**Figure 1:**
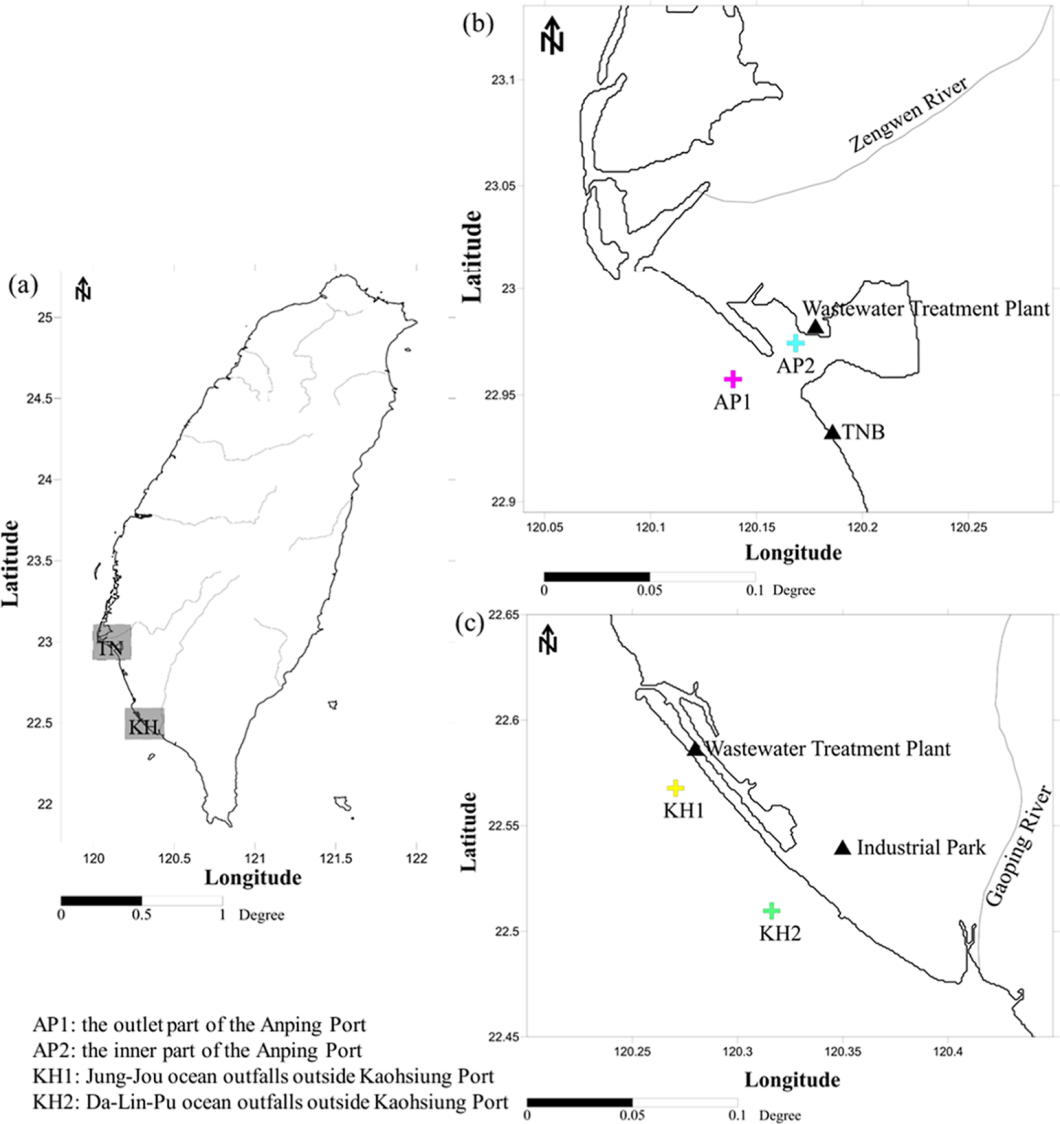
Coastal sampling locations in this study. (a) Sampling sites on the southwestern coast of Taiwan. (b) Two sampling sites in the outlet and inner part of the Anping Port in the Tainan area, and (c) two sampling sites near the ocean outfalls of wastewater treatment facilities outside of the Kaohsiung Port.

Seawater and MP samples were collected from two commercial ports in southwestern Taiwan (Figure 1a). Two sampling sites, AP1 and AP2, were located near the outlet and inner part of the Anping Port in the Tainan area (TN), as shown in Figure 1b. In the Kaohsiung area (KH), KH1 and KH2 were located near the Jung-Jou and Da-Lin-Pu ocean outfalls outside the Kaohsiung Port, respectively, as shown in Figure 1c. The four sampling sites were adjacent to wastewater treatment plants or industrial parks in TN and KH. An additional site (TNB) on Tainan Beach from our previous study was presented as a terrestrial reference for anthropogenic activities in Tainan’s coastal area.^18^

**Figure 2:**
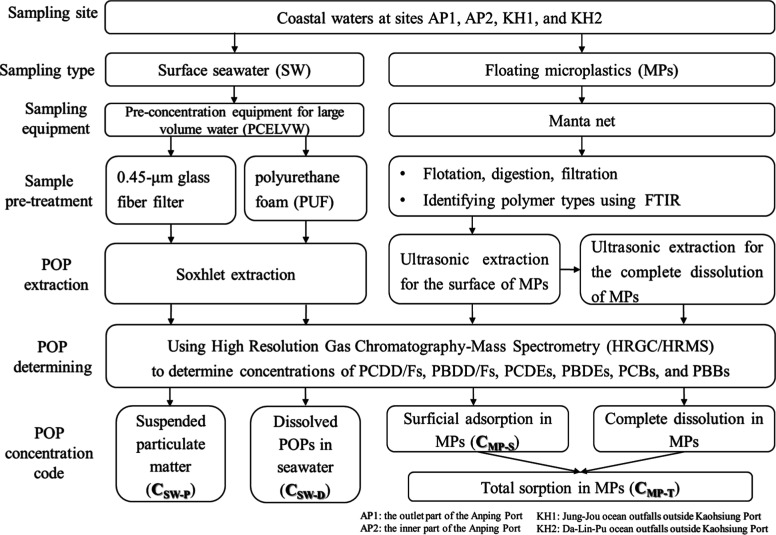
Sampling and preparation procedure in this study. The concentrations of POPs in SPM and dissolved POPs in seawater were distinguished (i.e., *C*_SW-P_ and *C*_SW-D_), and POPs in the surficial adsorption and total sorption in MPs were distinguished (i.e., *C*_MP-S_ and *C*_MP-T_).

The sample preparation and nomenclature of the fractions of POP concentrations in seawater and MPs are illustrated in the flowchart in Figure 2. The sampling method for POPs in seawater referred to the method of NIEA W790.51B by the Taiwan National Environmental Research Academy.^33,34^ Because of the relatively low concentrations of dissolved POPs in seawater, a large volume of seawater must be collected to ensure sufficient quantities of POPs for accurate measurement.^33^ A total of approximately 400 L of surface seawater was collected using pumps at sampling sites and then filtered using automatic preconcentration equipment for large volume water (PCELVW).^34^ After the seawater was injected into the PCELVW by a peristaltic pump, the SPM captured by glass fiber filters with a pore size of 0.5 μm was marked as SW-P, while the dissolved POPs adsorbed subsequently by polyurethane foam (PUF) were marked as SW-D.^34^ During the filtration process, the glass fiber filters were replaced periodically to prevent the breaking of particulates. The filters and PUF were removed from the PCELVW and stored in brown glass bottles for subsequent analysis of POPs in SPM and dissolved POPs in seawater. All sampling tools, equipment, and containers were made from nonplastic materials to minimize plastic contamination. When the filter paper and PUF were replaced, the tweezers and containers used were rinsed three times with filtered distilled water prior to use. Detailed information on the sampling method for POPs in seawater is described in Section S1 of the Supporting Information.

MP samples in surface seawater were collected using a modified Manta net (net opening of 55 cm width × 25 cm height, net length of 180 cm, net mesh of 330 μm) by stern towing for 30 min^35^ A mechanical flow meter was attached to the net port to estimate the amount of seawater flowing into the net. After the net was recovered back to the ship’s deck, MP samples intercepted by the net were flushed into stainless-steel sieves with a mesh of 250 μm and carefully transferred into a 1 L brown glass bottle. Six tows were collected at each sampling site to obtain sufficient samples of MPs. The total amounts of filtered seawater were 1553, 1085, 1770, and 1327 m^3^ at sites AP1, AP2, KH1, and KH2, respectively.

### 2.2 MPs’ Isolation and Identification

The MP samples were isolated through sieving, digestion, flotation, and filtration procedures.^35^ First, the MP samples in the brown glass bottle were carefully flushed into a combined stainless-steel sieve of 1 mm and 250 μm mesh. The MPs remaining on the 1 mm mesh sieve were visually picked up using a stainless-steel tweezer and transferred to Petri dishes for further microscopic observation. Next, the residues remaining on the 250 μm mesh sieve were carefully flushed into a 250 mL separatory funnel with a saturated sodium chloride (NaCl) solution (density 1.2 g/cm^3^). After 6 h of flotation, the precipitated material leaked through the bottom of the funnel. The floating materials were digested with 35% hydrogen peroxide (H_2_O_2_) at room temperature (25 °C) for 12 h to remove the organic components. After digestion, the floating materials were filtered through glass fiber filters (Advantec GS-25, Tokyo, Japan) with a pore size of 0.6 μm and dried in a desiccator. Subsequently, the MPs on the glass fiber filter were carefully transferred to Petri dishes using stainless-steel tweezers under a stereomicroscope (Euromex, Nexius Zoom NZ1903-P, Arnhem, The Netherlands). The number and morphological characteristics of MPs, including their size, shape, and color, were recorded. The polymer types of the MPs were identified using a Fourier-transform infrared microscope (Thermo Scientific Nicolet iN10, MA, USA) and verified based on the spectrum using Thermo Scientific OMNIC Specta software.^35^ After identification of the plastic type, MPs from each sampling site were stored in a glass vial for further POP analysis.

### 2.3 POP Extraction and Analysis

The determination of POPs in seawater referred to the method of NIEA M801.13B, M802.00B, and M803.00B by the Taiwan National Environmental Research Academy, which were modified from US-EPA M1613B, M8290A, M1614, and M1668, respectively. The particulates captured by the filters were used to determine the concentration of POPs in the SPM (*C*_SW-P_), whereas PUF was used to determine the concentration of dissolved POPs (*C*_SW-D_) in seawater. Before extraction, both the filter and PUF samples were spiked with a labeled ^13^C_12_-isotope internal standard solution of PCDD/Fs, PBDD/Fs, PBDEs, dioxin-like PCBs, and PBBs. The filter and PUF samples were placed in a Soxhlet extractor with toluene. After the filter and PUF samples were extracted using Soxhlet extraction for 24 h, the extracts were collected and concentrated under reduced pressure.^33,34^

The identified MPs were used to determine the concentrations of POPs in surficial adsorption (marked as *C*_MP-S_) and total sorption (marked as *C*_MP-T_). Currently, there are no uniform procedures for determining POPs in MPs. The extraction and analysis methods for POPs in MPs were performed according to the protocol established previously in our previous studies.^18,36,37^ According to Table S3 in the Supporting Information, previous research has utilized ultrasonic extraction and various solvents to dissolve plastics for analyzing organic pollutants. Therefore, in this study, ultrasonic extraction was used to extract the POPs in the MPs. The MPs were subjected to chemical analyses to first determine POPs in surficial adsorption (*C*_MP-S_) and then to determine the total POP contents (*C*_MP-T_).^18^ Initially, after adding 50 mL of *n*-hexane to the extractor, ultrasonic extraction was performed at least three times for 15 min to extract the POPs adsorbed on the surface of the MPs, and the extracts were then collected to determine *C*_MP-S_. Subsequently, the MPs were sequentially extracted by (1) adding 5 mL of tetrahydrofuran (THF) with ultrasonic extraction twice for 30 min each time, (2) adding 5 mL of *n*-hexane with ultrasonic extraction for 30 min, and (3) adding 5 mL of toluene with ultrasonic extraction for 30 min. The MPs were extracted until complete dissolution, and the extracts were collected to determine *C*_MP-T_.^18,37^

The extracts were concentrated to near dryness under reduced pressure and subjected to acid pickling by using concentrated sulfuric acid. Thereafter, the extracts were purified and separated using silica gel, alumina, and activated carbon. Finally, the extracts were reconcentrated to 1 mL and transferred to glass vials. These concentrated extracts were injected into a high-resolution gas chromatograph (Hewlett-Packard 6970 Series gas, CA)/high-resolution mass spectrometer (Micromass Autospec Ultima, Manchester, UK) to determine the POP content. Detailed information on the analytical procedures, quality assurance, and quality control for analyzing POPs in MPs is included in Section S2 of the Supporting Information.

### 2.4 Enrichment Factor Analysis and Toxic Equivalents

The analysis of the enrichment factor from previous research was slightly modified and applied to this study.^38−40^ The enrichment factor in this study was used to describe the concentration ratio of POPs in MPs or SPM relative to those of dissolved POPs in seawater. The enrichment factor was calculated using the following equation
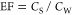
1where *C*_S_ represents the concentration of POPs in the MPs or SPM, while *C*_W_ is the concentration of dissolved POPs in seawater. To calculate the enrichment factor, *C*_MP-T_ and *C*_SW-P_ were calculated based on the MPs and particulates per unit weight, respectively, whereas *C*_SW-D_ was calculated based on the seawater per unit volume. Therefore, the enrichment factor for POPs in floating MPs was defined as the MP-dissolved phase partition coefficient (*C*_MP-T_ divided by *C*_SW-D_), denoted as EF_MP-T_. Likewise, the enrichment factor in SPM was defined as the particulate-dissolved phase partition coefficient (*C*_SW-P_ divided by *C*_SW-D_), denoted as EF_SW-P_.

The toxic equivalents (TEQs) of individual PCDD/Fs, PBDD/Fs, and dioxin-like PCB congeners were calculated using The World Health Organization 2005 toxic equivalency factors (WHO2005-TEFs).^41^ The TEQs were calculated using the following equation
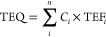
2where *C*_*i*_ represents the concentration of the POP congener, while TEF_*i*_ is the toxic equivalency factor of the POP congener.

## 3 Results and Discussion

### 3.1 Characteristics of Floating MPs

**Figure 3:**
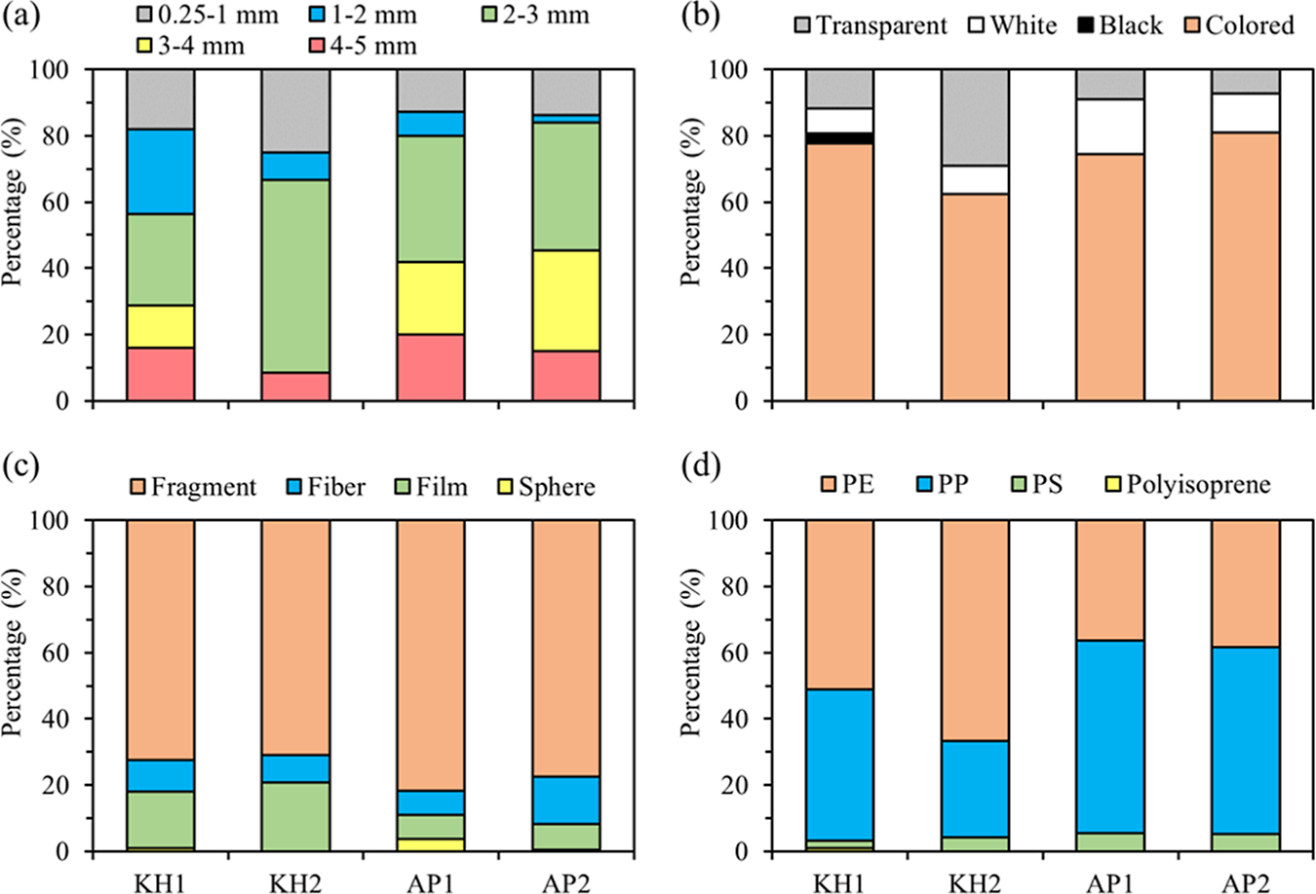
Percentages of (a) sizes, (b) colors, (c) shapes, and (d) polymer types of floating MPs based on the number.

The total number of MPs in AP1, AP2, KH1, and KH2 was 55, 232, 94, and 24, respectively. The abundance of MPs was 0.0354, 0.214, 0.0531, and 0.0181 items/m^3^ in AP1, AP2, KH1, and KH2, respectively. As shown in Figure 3a, the dominant sizes of MPs were 0.25 to 3 mm, which accounted for 58.2%, 54.7%, 71.3%, and 91.7% for AP1, AP2, KH1, and KH2, respectively. The MPs smaller than 250 μm were not investigated in this study as they are too light to meet the minimum weight required for trace detection of POPs. The larger MPs were dominant and may more easily absorb or release organic chemicals.^42^ In this study, the colors of MPs were classified into transparent, white, black, and colored, and the proportion of colored MPs was 74.5%, 81.0%, 77.7%, and 62.5% in AP1, AP2, KH1, and KH2, respectively (Figure 3b). As shown in Figure 3c, MPs were classified into fragments, fibers, films, and spheres. The MPs from all sampling sites were dominated by fragment shape, and the proportions of fragment shapes at sites AP1, AP2, KH1, and KH2 were 81.8%, 77.6%, 72.3%, and 70.8%, respectively. In addition, spherical MPs were extremely low at the four sampling sites. In this study, the polymer types of the MPs were identified as polyethylene (PE), polypropylene (PP), polystyrene (PS), and polyisoprene using FTIR spectroscopy. For both Kaohsiung sites, PE was the dominant type, accounting for 51.1% and 66.7% for KH1 and KH2, respectively, whereas PP was the dominant type at the Tainan sites, accounting for 58.2% and 56.5% for TN1 and TN2, respectively (Figure 3d). Other studies have also indicated that PE and PP are the major polymers of MPs in surface seawater in eastern and southwestern Taiwan.^35,43^ The dominant polymer types of MPs in surface seawater in this study were similar to those in other studies in Taiwan.

### 3.2 POPs in Seawater

**Figure 4:**
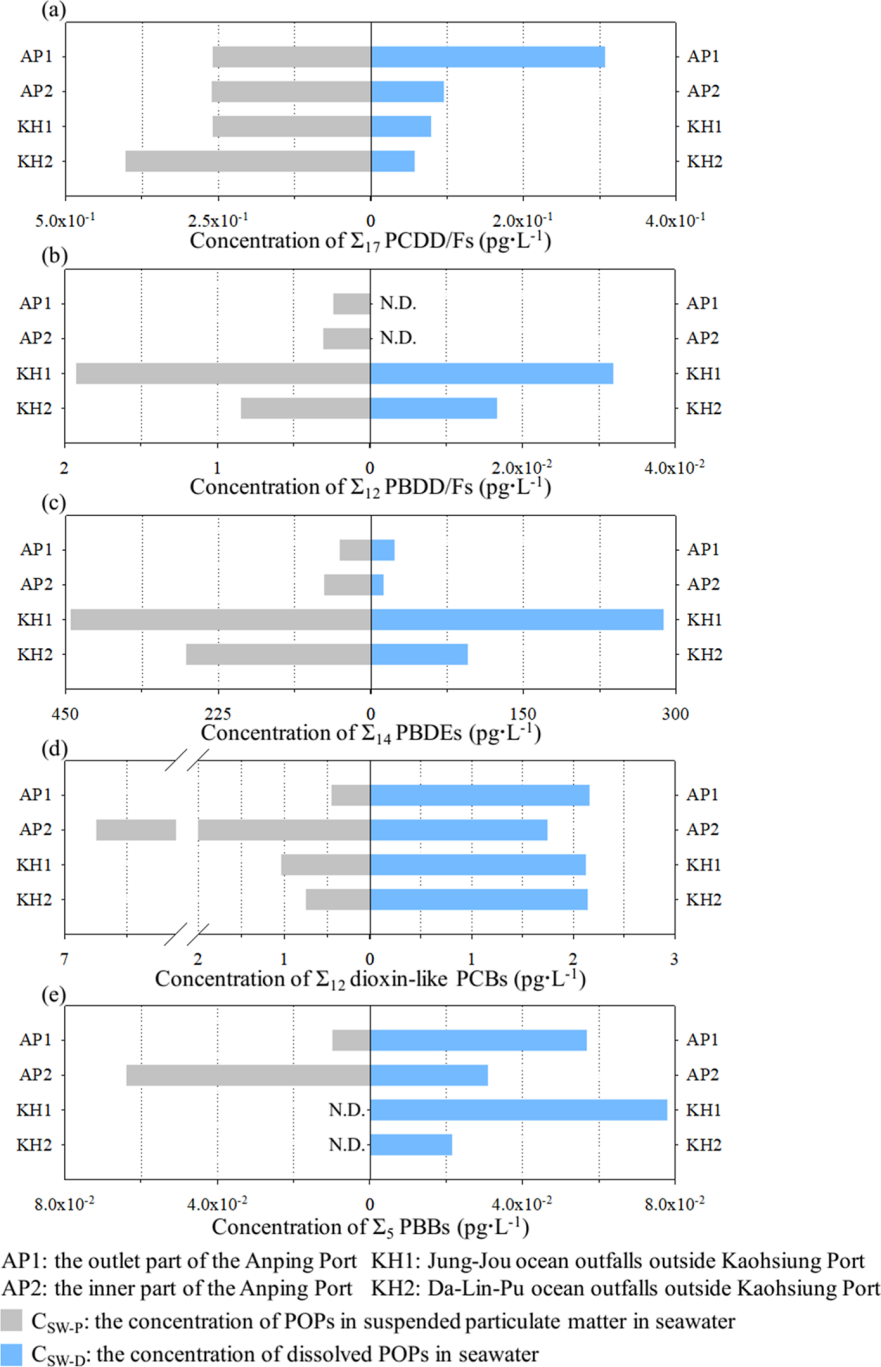
Concentration of POPs in SPM (*C*_SW-P_, marked as gray bars) and dissolved POPs (*C*_SW-D_, marked as blue bars) in seawater at four sampling sites. (a) Σ_17_ PCDD/Fs, (b) Σ_12_ PBDD/Fs, (c) Σ_14_ PBDEs, (d) Σ_12_ dioxin-like PCBs, and (e) Σ_5_ PBBs.

Figure 4 shows the POP concentrations in the seawater. The *C*_SW-P_ and *C*_SW-D_ values were calculated based on seawater per unit volume. In the gray bars in Figure 4a, the *C*_SW-P_ for Σ_17_ PCDD/Fs sampled from the four coastal sites (i.e., AP1, AP2, KH1, and KH2) ranged from 2.59 × 10^–1^ to 4.02 × 10^–1^ pg·L^–1^. Pouch et al. (2021) documented that the concentration of Σ_16_ PCDD/Fs in SPM in the Arctic seawater ranged predominantly from 6.60 × 10^–2^ to 7.90 × 10^–1^ pg·L^–1^, with only a few samples exhibiting high concentrations.^44^ The *C*_SW-P_ for Σ_17_ PCDD/Fs in our data was within the range reported by Pouch et al. (2021).^44^ As shown by the blue bars in Figure 4a, the *C*_SW-D_ for Σ_17_ PCDD/Fs in AP1 was 3.07 × 10^–1^ pg·L^–1^, and it dropped to 9.57 × 10^–2^, 7.88 × 10^–2^, and 5.74 × 10^–2^ pg·L^–1^ in the other three sampling sites. The average *C*_SW-D_ from the last three sites was only 7.73 × 10^–2^ pg·L^–1^ (SD of 1.91 × 10^–2^), which was approximately one-quarter of the *C*_SW-D_ at AP1. The *C*_SW-D_ for Σ_17_ PCDD/Fs in our results was lower than the concentrations of dissolved Σ_17_ PCDD/Fs in the river water (2.66 × 10^0^ to 4.60 × 10^0^ pg·L^–1^) reported by Liu et al. (2008).^45^ Additionally, Thuan et al. (2011) indicated that the mean total concentration of Σ_17_ PCDD/Fs in the surface water and groundwater of Taiwan’s terrestrial hydrosphere was 2.41 × 10^1^ and 3.27 × 10^0^ pg·L^–1^, respectively.^33^ In our study, the average sum of *C*_SW-P_ and *C*_SW-D_ for Σ_17_ PCDD/Fs in seawater was 4.30 × 10^–1^ pg·L^–1^, which was 1–2 orders of magnitude lower than those reported by Thuan et al. (2011).^33^ In the seaward part of Kaohsiung Port, the total concentration of Σ_17_ PCDD/Fs in seawater was 1.33 × 10^0^ and 2.31 × 10^–1^ pg·L^–1^ at two sampling sites, which were comparable to our findings.^46^ Obviously, the concentration of PCDD/Fs in terrestrial water is higher compared to that in seawater.

In the gray bars in Figure 4b, the *C*_SW-P_ for Σ_12_ PBDD/Fs in KH1 was detected to be as high as 1.93 × 10^0^ pg·L^–1^. The second highest Σ_12_ PBDD/Fs was determined as 8.49 × 10^–1^ pg·L^–1^ in KH2. The *C*_SW-P_ for Σ_12_ PBDD/Fs at site AP1 and AP2 were 2.42 × 10^–1^ and 3.06 × 10^–1^ pg·L^–1^, respectively. The *C*_SW-P_ for Σ_12_ PBDD/Fs at site KH1 was 4.1 times higher than the mean concentrations of the other three sites. In terms of the dissolved PBDD/Fs in seawater, the *C*_SW-D_ for Σ_12_ PBDD/Fs was detected at much lower concentrations in Kaohsiung (3.19 × 10^–2^ pg·L^–1^ in KH1 and 1.67 × 10^–2^ pg·L^–1^ in KH2) or not detected at all in samples from Anping Port (the blue bars in Figure 4b). In this study, the average sum of *C*_SW-P_ and *C*_SW-D_ for Σ_12_ PBDD/Fs was 8.44 × 10^–1^ pg·L^–1^. Wardiani et al. (2024) reported that the concentrations of Σ_12_ PBDD/Fs in seawater near the seaward part of Kaohsiung Port were 7.2 × 10^–1^ and 1.95 × 10^–1^ pg·L^–1^, which fall within the same range as our findings.^46^ Generally, the main source of PBDD/Fs is the byproduct of industrial chemicals due to combustion and incineration.^47^ Additionally, PBDD/Fs have occurred in marine primary producers and are produced by marine algae.^48^ The current information on PBDD/Fs in seawater is limited, so the exposure of marine organisms to PBDD/Fs in marine environments is unclear.

As shown in Figure 4c, the concentrations of the Σ_14_ PBDEs were higher than those of the other POPs. The highest concentration of Σ_14_ PBDEs among the four sampling sites was registered at KH1, either in the SPM at a concentration of 4.43 × 10^2^ pg·L^–1^ or in the dissolved PBDEs at a concentration of 2.88 × 10^2^ pg·L^–1^. The second highest *C*_SW-P_ for Σ_14_ PBDEs was sampled in KH2 (2.72 × 10^2^ pg·L^–1^). The *C*_SW-P_ in AP1 and AP2 were 4.66 × 10^1^ and 6.89 × 10^1^ pg·L^–1^, respectively. The *C*_SW-D_ in KH2, AP1, and AP2 were determined as 9.50 × 10^1^, 2.39 × 10^1^, and 1.29 × 10^1^ pg·L^–1^, respectively. The highest *C*_SW-P_ and *C*_SW-D_ for Σ_14_ PBDEs were 9.5 and 22.3 times higher than the lowest concentration, respectively. Ju et al. (2016) indicated that the concentration of Σ_14_ PBDEs in SPM and dissolved Σ_14_ PBDEs in seawater was 1.17 × 10^0^ to 5.45 × 10^0^ and 9 × 10^–2^ to 1.35 × 10^0^ ng·L^–1^, respectively, in the bay of China.^49^ Moreover, the concentrations of Σ_8_ PBDEs in the dissolved phase in seawater were 1.54 × 10^1^ to 6.55 × 10^1^ ng·L^–1^ in the coastal waters of the Bohai Sea.^50^ A comparison revealed that the *C*_SW-P_ and *C*_SW-D_ values for PBDEs in this study were lower than those in other areas. Additionally, the average sum of *C*_SW-P_ and *C*_SW-D_ for Σ_14_ PBDEs was 3.13 × 10^2^ pg·L^–1^. In the seaward part of Kaohsiung Port, the total concentration of Σ_14_ PBDEs in seawater was 1.83 × 10^2^ and 6.24 × 10^1^ pg·L^–1^, which were slightly lower than our findings.^46^ In terms of congeners, the mean concentration of BDE#209(10Br) was the most abundant congener in the SPM and dissolved PBDEs in seawater, as shown in Figure S2.

The *C*_SW-P_ for Σ_12_ dioxin-like PCBs sampled from AP2 was much higher (6.74 × 10^0^ pg·L^–1^) than those sampled from the other three sites, as indicated by the gray bars in Figure 4d. The *C*_SW-P_ for Σ_12_ dioxin-like PCBs in AP1, KH1, and KH2 were 4.47 × 10^–1^, 1.04 × 10^0^, and 7.45 × 10^–1^ pg·L^–1^, respectively. In the blue bars in Figure 4d, the *C*_SW-D_ for Σ_12_ dioxin-like PCBs in AP2 was smaller (1.74 pg·L^–1^) than those of the other three sites (mean of 2.14 pg·L^–1^). In the seawater of Arctic fjords, the concentrations of Σ_7_ PCBs in SPM and dissolved PCBs were 2 × 10^–3^ to 4.12 × 10^1^ and 4 × 10^–3^ to 4 × 10^–1^ ng·L^–1^, respectively.^51^ The *C*_SW-D_ for Σ_12_ dioxin-like PCBs determined in this study were within the range reported by Pouch et al. (2021).^51^ Additionally, in this study, the average sum of *C*_SW-P_ and *C*_SW-D_ for Σ_12_ dioxin-like PCBs in seawater was 4.28 × 10^0^ pg·L^–1^. This result was comparable to the concentrations of Σ_12_ dioxin-like PCBs in both terrestrial water and seawater, as reported by Nguyen et al. (2017) and Wardiani et al. (2024), respectively.^34,46^ Additionally, in terms of congeners, PCB#118(5CL) was the most abundant congener in seawater in this study, as shown in Figure S2.

As shown in Figure 4e, the *C*_SW-P_ for Σ_5_ PBBs was detected only outside (AP1) and inside (AP2) the Anping Port. The *C*_SW-P_ in AP2 (6.38 × 10^–2^ pg·L^–1^) was higher than the *C*_SW-P_ in AP1 (9.89 × 10^–3^ pg L^–1^). However, PBBs were not detected at KH1 and KH2 near the Kaohsiung Port as both were located 3 km away from the shore. In contrast, the *C*_SW-D_ for Σ_5_ PBBs observed in AP1, AP2, KH1, and KH2 was 5.68 × 10^–2^, 3.10 × 10^–2^, 7.81 × 10^–2^, and 2.17 × 10^–2^ pg·L^–1^, respectively. In terms of congeners, PBB#15(2Br) was the most abundant congener in dissolved PBBs in seawater, as shown in Table S22.

### 3.3 POPs within MPs in Surficial Adsorption and Total Sorption

**Figure 5:**
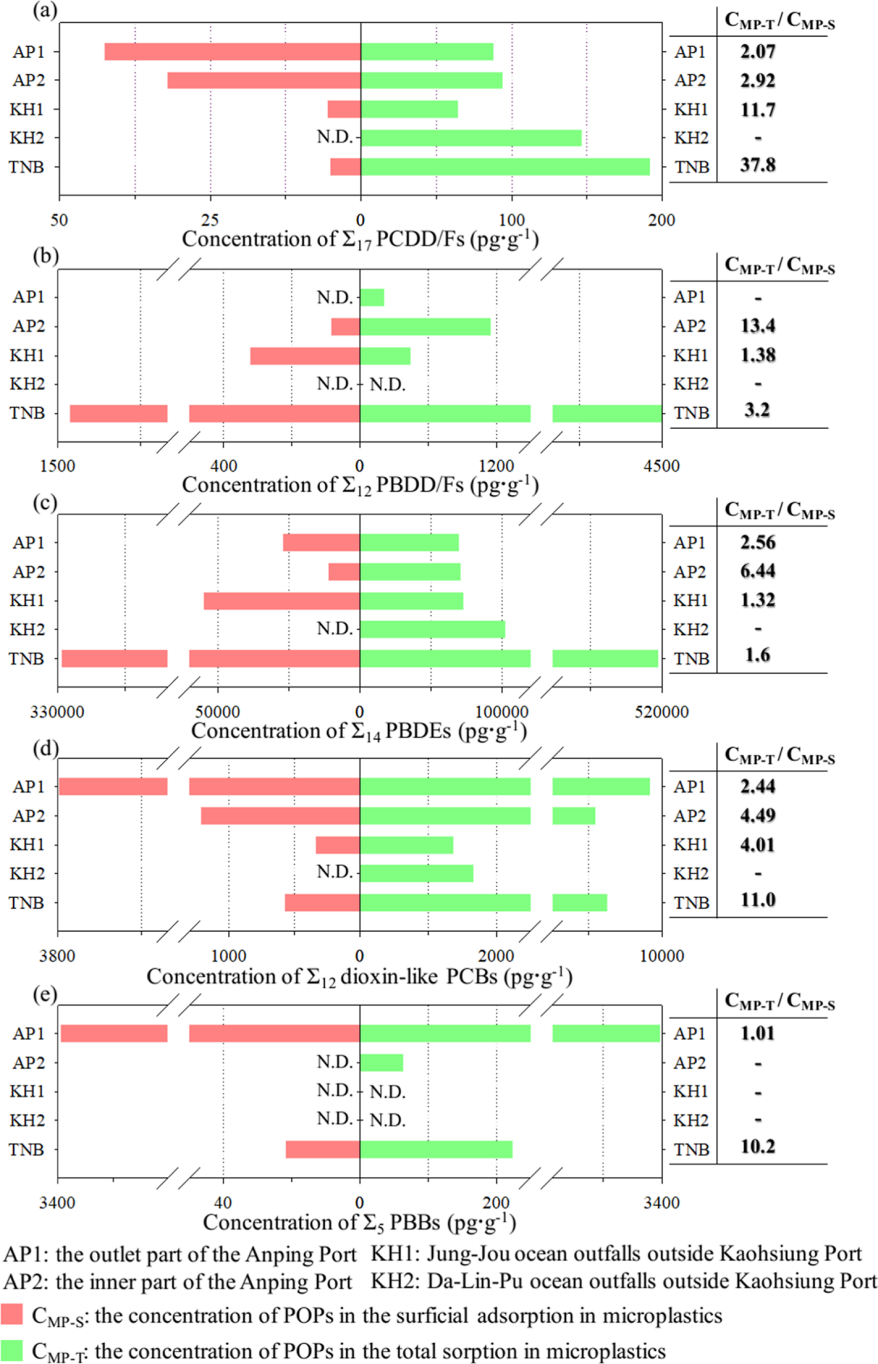
POP concentration in the surficial adsorption (*C*_MP-S_, marked as red bars) and in the total sorption (*C*_MP-T_, marked as green bars) within floating MPs from four sampling sites. Bold numbers represent the ratios of *C*_MP-T_ to *C*_MP-S_. *C*_MP-S_ in KH2 could not be determined due to the lower amount and weight of MPs. (a) Σ_17_ PCDD/Fs, (b) Σ_12_ PBDD/Fs, (c) Σ_14_ PBDEs, (d) Σ_12_ dioxin-like PCBs, and (e) Σ_5_ PBBs.

Figure 5 shows the POP concentrations in the MPs. The *C*_MP-S_ and *C*_MP-T_ values were calculated based on MPs per unit weight. In the red bars in Figure 5a, the *C*_MP-S_ for Σ_17_ PCDD/Fs in AP1, AP2, KH1, and KH2 were 4.26 × 10^1^, 3.22 × 10^1^, 5.50 × 10^0^ pg·g^–1^, and N.D., respectively. Wang et al. (2021) found that the concentration of Σ_17_ PCDD/Fs in the surficial adsorption within MP pellets on Tainan beach (TNB) was 5.1 × 10^0^ pg·g^–1^.^18^ The *C*_MP-S_ for Σ_17_ PCDD/Fs from seawater around the Anping Port in the Tainan area were higher than those from TNB. The *C*_MP-T_ for Σ_17_ PCDD/Fs from the four sites were 8.81 × 10^1^, 9.38 × 10^1^, 6.44 × 10^1^, and 1.47 × 10^2^ pg·g^–1^, respectively, as shown by the green bars in Figure 5a. However, the *C*_MP-T_ for Σ_17_ PCDD/Fs determined in TNB was up to 1.92 × 10^2^ pg·g^–1^, which was almost double the *C*_MP-T_ in AP1 and AP2. Our data indicated that a higher *C*_MP-T_ for Σ_17_ PCDD/Fs was observed on the beach than in the port area in Tainan. Moreover, the *C*_MP-S_ for Σ_17_ PCDD/Fs in MPs from seawater in AP1, AP2, and KH1 were higher than those in MP pellets from the beach in the TNB. This observation may indicate that weathering mechanisms, such as hydrolysis or sunlight photodegradation in marine environments, contribute to the aging of MPs’ surface, resulting in enhanced adsorption of pollutants due to increased surface roughness of MPs.^52,53^ Additionally, the distribution pattern of congener profiles of Σ_17_ PCDD/Fs in MPs in this study (Figures S3 and S4) was similar to that reported by Wang et al.^18^ The primary congeners of PCDD/Fs found in MPs were species with a higher number of chlorines or bromines, including HpCDD/Fs and OCDD/Fs. The detailed results and discussion are provided in Section S3 of the Supporting Information.

In Figure 5b, the *C*_MP-S_ for Σ_12_ PBDD/Fs were only detected at sites AP2 and KH1, registered at 8.56 × 10^1^ and 3.22 × 10^2^ pg·g^–1^, respectively. The *C*_MP-T_ for Σ_12_ PBDD/Fs in AP1, AP2, and KH1 were detected as 2.17 × 10^2^, 1.15 × 10^3^, and 4.44 × 10^2^ pg·g^–1^, respectively (the green bars in Figure 5b). The *C*_MP-T_ for Σ_12_ PBDD/Fs sampled from AP2 was 5.3 and 2.6 times higher than those of the other two sites (i.e., AP1 and KH1). Wang et al. (2021) indicated that the overall concentrations of Σ_12_ PBDD/Fs within MP pellets from the sandy beaches of Taiwan were 3.92 × 10^3^ to 2.93 × 10^4^ pg·g^–1^.^18^ Comparing the previous study with this study, it was found that the *C*_MP-T_ for Σ_12_ PBDD/Fs within floating MPs from seawater was lower than that of MP pellets from beaches.

As shown in Figure 5c, the sums of the 14 PBDE concentrations detected on the surface and whole floating MPs were comparatively higher than those of the other POPs in this study. The highest *C*_MP-S_ for Σ_14_ PBDEs was sampled from KH1 (5.50 × 10^4^ pg·g^–1^), following those sampled from AP1 (2.71 × 10^4^ pg·g^–1^) and AP2 (1.10 × 10^4^ pg·g^–1^). The *C*_MP-T_ for Σ_14_ PBDEs within MPs sampled from the three sites (AP1, AP2, and KH1) were found to be close, with values of 6.95 × 10^4^, 7.07 × 10^4^, and 7.26 × 10^4^ pg·g^–1^, respectively. Only the *C*_MP-T_ for Σ_14_ PBDEs sampled from the KH2 showed a much higher value at 1.02 × 10^5^ pg·g^–1^, but the *C*_MP-S_ was not detected. In the North Pacific accumulation zone, the concentrations of Σ_15_ PBDEs in floating plastics with sizes less than 5 mm were 5.10 × 10^3^ to 3.23 × 10^4^ pg·g^–1^.^54^ Yeo et al. (2020) found that the concentrations of Σ_19_ PBDEs within MP particles in seawater were 2.28 × 10^3^ to 8.34 × 10^5^ pg·g^–1^ at Tokyo Bay and 1.64 × 10^4^ to 2.21 × 10^6^ pg·g^–1^ at Sagami Bay.^55^ Compared with other studies showing large fluctuations, the Σ_14_ PBDE concentration within MPs varied slightly in this study.

In the red bars in Figure 5d, the concentrations of Σ_12_ dioxin-like PCBs in surficial adsorption within floating MPs were determined at three sites (AP1, AP2, and KH1), with values of 3.77 × 10^3^, 1.21 × 10^3^, and 3.41 × 10^2^ pg·g^–1^, respectively. The *C*_MP-S_ for Σ_12_ dioxin-like PCBs sampled from AP1 was 3.1 and 11.1 times higher than that sampled from AP2 and KH1, respectively. The *C*_MP-T_ for Σ_12_ dioxin-like PCBs in the four sampling sites (AP1, AP2, KH1, and KH2) were all detected, with values of (9.20 × 10^3^, 5.44 × 10^3^, 1.37 × 10^3^, and 1.66 × 10^3^ pg·g^–1^), as shown by the green bars in Figure 5d. There was a similar trend in total sorption and surficial adsorption among the three sampling sites (for the quantity of Σ_12_ dioxin-like PCB concentrations: (AP1 > AP2 > KH1). In Japan, the concentrations of Σ_13_ PCBs within MP particles sampled from seawater were 2.88 × 10^3^ to 1.24 × 10^5^ pg·g^–1^ at Tokyo Bay and 8.47 × 10^2^ to 5.77 × 10^4^ pg·g^–1^) at Sagami Bay.^54^ The Σ_12_ dioxin-like PCB concentrations within MPs in this study were much lower than those reported by Yeo et al. (2020).^55^

As shown in Figure 5e, the *C*_MP-S_ for Σ_5_ PBBs were only detected in AP1, registered at 3.31 × 10^3^ pg·g^–1^, which was quite close to its *C*_MP-T_ (3.34 × 10^3^ pg·g^–1^). However, the *C*_MP-T_ of the PBBs within the MPs sampled from AP2 was much lower (6.32 × 10^1^ pg·g^–1^). Neither *C*_MP-S_ nor *C*_MP-T_ for Σ_5_ PBBs from the MPs sampled at KH1 and KH2 were detected. However, the *C*_MP-T_ for Σ_5_ PBBs within floating MPs in seawater from AP1 was almost 15 times higher than that of MP pellets on beaches from TNB. Gieroń et al. (2010) reported that the concentration of Σ_5_ PBBs ranged from 4.50 × 10^–1^ to 2.69 × 10^2^ pg·g^–1^ in the muscle tissue of fish from Baltic and North seas.^56^ The *C*_MP-T_ for PBBs in this study was higher than the PBB concentration within marine organisms.

In Figure 5, the bold numbers on the right indicate the ratios of *C*_MP-T_ to *C*_MP-S_ (*C*_MP-T_/*C*_MP-S_). For the Σ_17_ PCDD/Fs in Figure 5a, the highest ratio of *C*_MP-T_/*C*_MP-S_ was 11.7 for KH1, followed by the ratios for AP2 and AP1. Moreover, the *C*_MP-T_/*C*_MP-S_ ratio of the PBDEs was 6.44 for AP2, which was higher than that of AP1 and KH1 (Figure 5c). As shown in Figure 5b,d,e, the highest ratios of *C*_MP-T_/*C*_MP-S_ for the PBDD/Fs, dioxin-like PCBs, and PBBs were 13.4, 4.49, and 1.01, respectively. Wang et al. (2021) found that the highest ratios of total sorption to surficial adsorption for PCDD/Fs and PBDEs on MP pellets were 355 and 8,069, respectively.^18^ The ratios of *C*_MP-T_/*C*_MP-S_ for PCDD/Fs and PBDEs in this study were much lower than those in the previous study. Compared to the MP pellets found on beaches, as reported by Wang et al. (2021),^18^ the predominant shape of floating MPs from seawater samples in this study was fragments, and MP fragments accounted for over 70% of the total number. The irregular MP fragments lead to differences in the specific surface area and distribution equilibrium of POPs on the particles. The heterogeneity of fragments causes differences in the surface/volume ratios of individual MPs, which affects the sorption of hydrophobic organic pollutants on MPs.^57,58^ Additionally, PBDEs may have been artificially added to MP pellets during the manufacturing stage to serve as a brominated flame retardant.^55^ However, PCDD/Fs and PBDD/Fs are not synthetic chemicals but are formed through precursor pathways or de novo synthesis during incomplete combustion.^59^ Given the longstanding ban on PCBs and PBBs, it is highly improbable that suppliers would add these chemicals during the manufacturing process.^60^ To determine the level of sorption of POPs in MPs in this study, virgin MP pellets from two manufacturing suppliers were preliminarily tested for concentrations of POPs. The test on virgin pellets indicated that the concentrations of POPs were either below the detection limit or present only in trace amounts, as shown in Table S41. Therefore, it is possible that the secondary fragmented MPs sorbed POPs from the environment.

### 3.4 Enrichment for POPs in MPs and SPM

**Figure 6:**
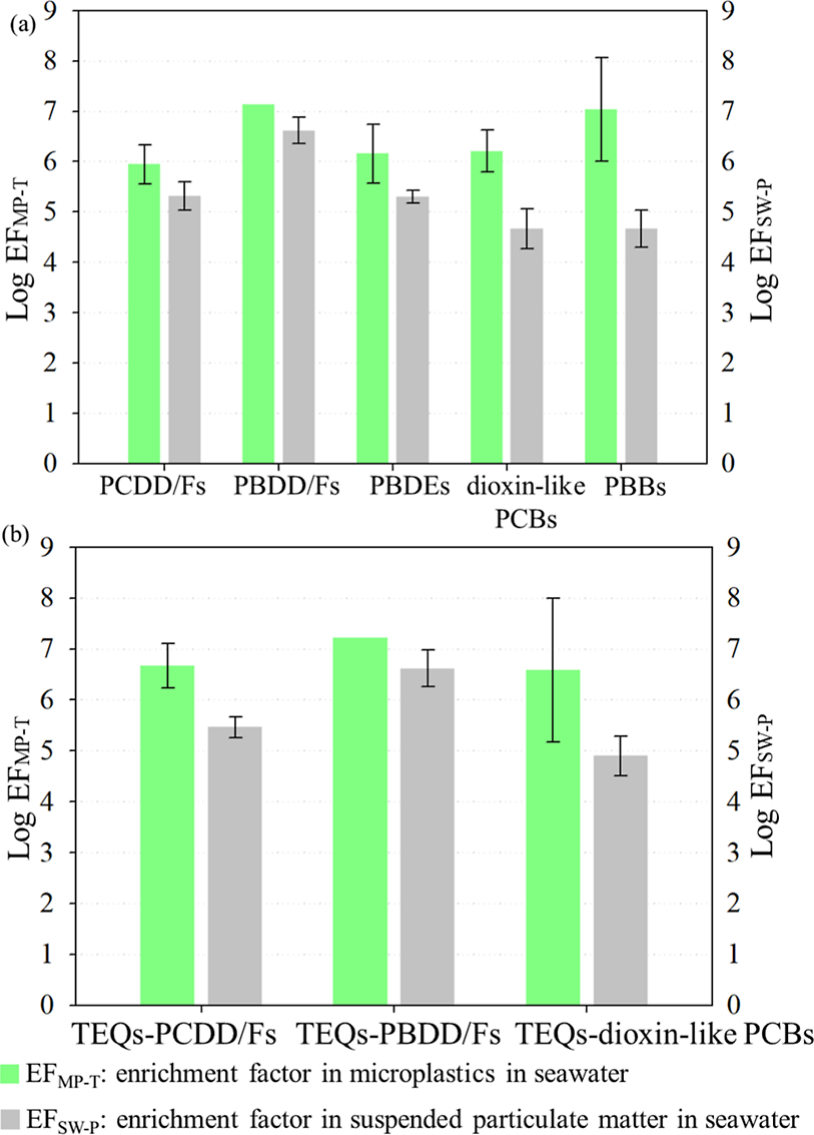
Mean and standard deviation in the log EF_MP-T_ (marked as green bars) and log EF_SW-P_ (marked as gray bars). (a) POP mass concentrations and (b) POP concentrations calculated by toxic equivalents (TEQs) of POP congeners.

Figure 6 shows the means and standard deviations of the logarithm of EF_MP-T_ (log EF_MP-T_) and EF_SW-P_ (log EF_SW-P_). The concentrating effect was evaluated by comparing the enrichment factor of MPs and SPM. In Figure 6a, the mean log EF_MP-T_ values of PCDD/Fs, PBDD/Fs, PBDEs, dioxin-like PCBs, and PBBs were 5.94, 7.14, 6.16, 6.21, and 7.04, respectively. The mean log EF_SW-P_ values of PCDD/Fs, PBDD/Fs, PBDEs, dioxin-like PCBs, and PBBs were 5.32, 6.62, 5.30, 4.67, and 4.67, respectively. According to the statistics of the Mann–Whitney *U* test (with a *p*-value of 0.047), log EF_MP-T_ was significantly higher than log EF_SW-P_. A comparison of these results revealed that the concentrating effect of floating MPs was greater than that of SPM. As shown in Figure 6a, for PCDD/Fs, PBDD/Fs, and PBDEs, the concentrating effect of floating MPs was approximately 0.5–1 order of magnitude higher than that of SPM in seawater. Moreover, the concentrating effect of floating MPs was approximately 1.5–2 and 2–2.5 orders of magnitude higher than that of SPM for dioxin-like PCBs and PBBs, respectively. Compared with SPM in seawater, the secondary fragmented floating MPs in this study sorb larger amounts of POPs from the environment. In sorption of hydrophobic organic pollutants, sediments also adsorb less organic pollutants (phenanthrene or nonylphenol) compared to MPs.^32,61^ Moreover, our data indicated that the log EF_MP-T_ of five POPs (i.e., PCDD/Fs, PBDD/Fs, PBDEs, dioxin-like PCBs, and PBBs) within floating MPs ranged from 5.94 to 7.14. Wright et al. (2013) indicated that the concentration of POPs, such as PCBs, within MPs was 5–6 orders of magnitude higher than that in seawater.^1^ However, it was found that the EF_MP-T_ for dioxin-like PCBs can exceed 6 orders of magnitude in this study. In general, the log EF_MP-T_ values in this study fall within the range of logarithm of distribution coefficient (log *K*_d_ or *K*_f_) of sorption between organic pollutants and MPs in review studies.^62^

In this study, the toxic equivalents (TEQs) of individual PCDD/Fs, PBDD/Fs, and dioxin-like PCB congeners were calculated using the WHO2005-TEFs.^41^Figure 6b displays the mean and standard deviation of log EF_MP-T_ and log EF_SW-P_ for TEQs-PCDD/Fs, TEQs-PBDD/Fs, and TEQs-dioxin-like PCBs. The mean log EF_MP-T_ values of TEQ-PCDD/Fs, TEQ-PBDD/Fs, and TEQ-dioxin-like PCBs were 6.67, 7.22, and 6.59, respectively. The mean log EF_SW-P_ values of the TEQ-PCDD/Fs, TEQ-PBDD/Fs, and TEQ-dioxin-like PCBs were 5.46, 6.62, and 4.90, respectively. The concentrating effect of floating MPs were approximately 1–1.5, 0.5–1, and 1.5–2 orders of magnitude higher than those of SPM in seawater for TEQs-PCDD/Fs, TEQs-PBDD/Fs, and TEQs-dioxin-like PCBs, respectively. Additionally, the concentrating effect of floating MPs was approximately 0.5–1/1 and 1–1.50 orders of magnitude higher than that of SPM for PCDD/Fs and TEQs-PCDD/Fs, respectively (Figure 6a,b). Comparing the results of PCDD/Fs and TEQs-PCDD/Fs, the concentrating effect differed by 0.5 orders of magnitude. This difference is attributed to the varying distributions of highly toxic congeners. In the congener profile of PCDD/Fs, the mean concentrations of the most toxic 1,2,3,7,8-PeCDD (WHO2005-TEF of 1) and 2,3,7,8-TeCDF (WHO2005-TEF of 0.1) within MPs were 9.79 × 10^0^ pg·g^–1^ (accounting for 8.11%) and 1.06 × 10^1^ pg·g^–1^ (accounting for 10.4%) (Figure S2a), respectively. Meanwhile, the mean concentrations of 1,2,3,7,8-PeCDD and 2,3,7,8-TeCDF in SPM were only 2.53 × 10^–3^ pg·L^–1^ (accounting for 0.86%) and 5.06 × 10^–3^ pg·L^–1^ (accounting for 1.26%) (Figure S1a), respectively.

In this study, the contents of volatile solids in SPM were 21.8% at site AP1 and 28.9% at KH1 (Table S40). Previous studies indicated that the content of organic matter in SPM can affect its capacity to adsorb hydrophobic organic pollutants.^63^ Therefore, assuming that all volatile solids are considered the organic matter in SPM and that the inorganic matter of SPM does not absorb POPs, the concentration of POPs in the organic matter of SPM is roughly calculated as shown in Tables S50–S54 in the Supporting Information. Moreover, based on the rough estimation of the enrichment factor for POPs in organic matter (EF_Organic_), the mean log EF_Organic_ of PCDD/Fs, PBDD/Fs, PBDEs, dioxin-like PCBs, and PBBs were 5.92, 7.22, 5.90, 5.27, and 5.27, respectively, as shown in Table S55 in the Supporting Information. A comparison of EF_MP-T_ and EF_Organic_ revealed that the concentrating effect of MPs was approximately 1–2 orders of magnitude higher than that of organic matter for dioxin-like PCBs and PBBs. For PCDD/Fs, PBDD/Fs, and PBDEs, the concentrating effect of organic matter is approximately comparable to that of MPs. However, in this study, it is important to underscore that the findings related to organic matter are rough estimates, and further experimental data are necessary to accurately assess the concentration of POPs in organic matter extracted from SPM. Nevertheless, the findings presented in the preceding paragraph indicate that highly toxic congeners, including 1,2,3,7,8-PeCDD and 2,3,7,8-TeCDF, are more likely to be sorbed and accumulated by secondary MPs than SPM in seawater. In a study on the sorption of POPs in primary MPs reported by Abihssira-García et al. (2022), the highly toxic congeners of PCDD/Fs, 1,2,3,7,8-PeCDD (WHO2005-TEF of 1), 2,3,7,8-TCDD (WHO2005-TEF of 1), and 1,2,3,4,7,8-HxCDD (WHO2005-TEF of 0.1), showed a higher binding affinity to MPs made of unplasticized polyvinyl chloride (uPVC) compared to high-density polyethylene (HDPE).^24^ Generally, the POPs within MPs are often dominated by highly chlorinated or brominated congeners.^18,55,64^ However, as discussed above, whether it is primary MPs from commercial production or secondary MPs in the marine environment, they also tend to sorb and accumulate highly toxic PCDD/F congeners. When highly toxic POP-enriched MPs enter marine organisms, the digestive fluid in the organism may lead to the desorption of toxic pollutants from the MPs.^32,65^ These trends showed the potential for toxicity to marine organisms if floating secondary MPs acted as vectors of highly toxic POPs.

### 3.5 Relationship between the Enrichment Factor and *K*_OW_ of POP Homologues

**Figure 7:**
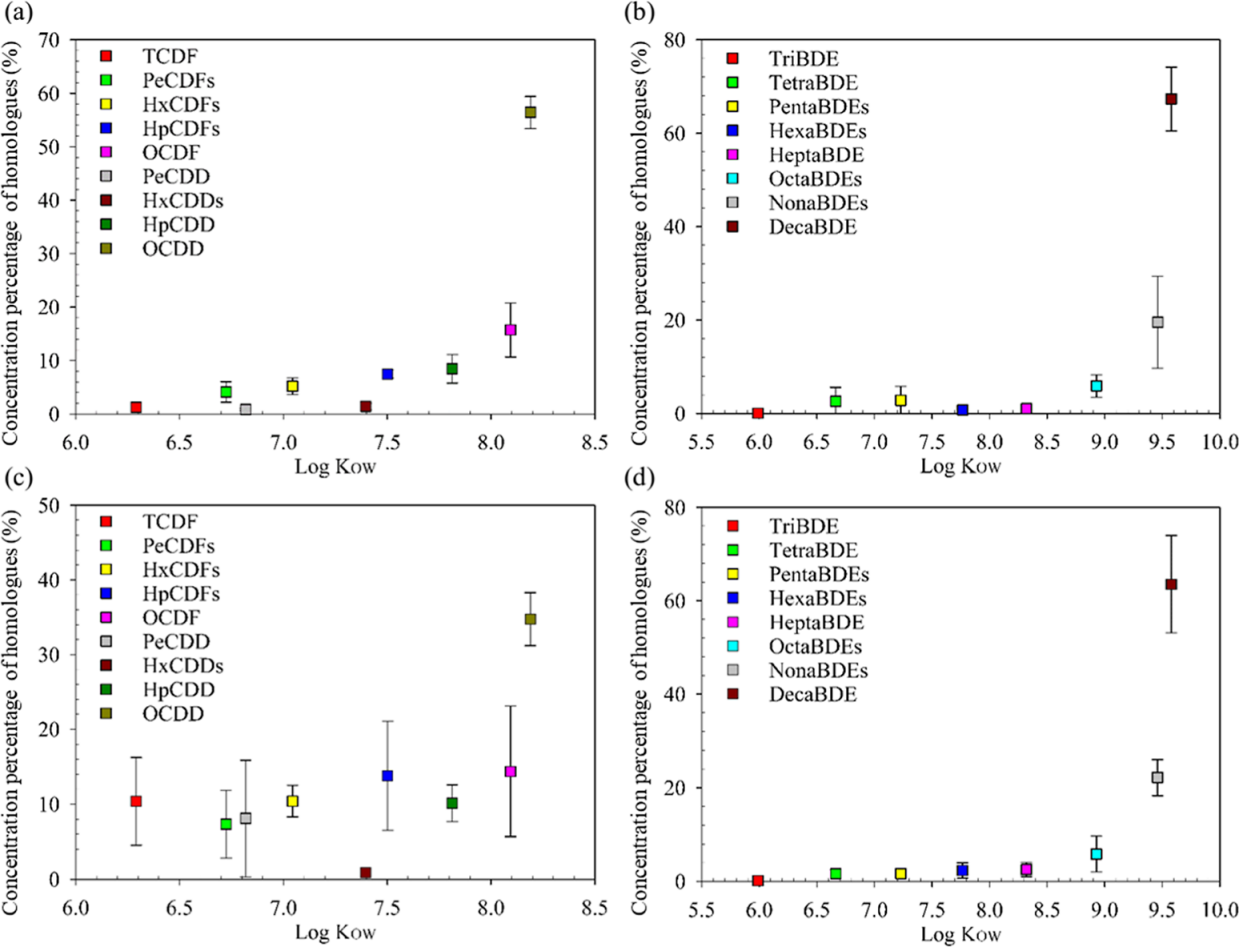
Relationships between log *K*_OW_ and total sorption concentration of PCDD/F and PBDE homologues. (a,b) In SPM in seawater. (c,d) In the floating MPs. The log *K*_OW_ of POP homologues were derived from previous studies (Tables S33).

The *n*-octanol–water partition coefficient (*K*_OW_) is commonly used as a hydrophobicity parameter, which is an important characteristic of the sorption behavior of organic compounds.^57,66−68^ In this study, Figure 7 illustrates the relationship between the concentration and *K*_OW_ of PCDD/F and PBDE homologues. Higher concentration percentages of highly chlorinated or brominated homologues, such as OCDD and DecaBDE, were observed in SPM compared to MPs (Figure 7a,b). In terms of marine floating MPs, the concentration of OCDD was the highest. With the exception of HxCDDs, the concentrations of other PCDD/F homologues were generally comparable (Figure 7c). In Figure 7d, the distribution pattern of PBDE homologues in MPs is generally consistent with the SPM in seawater. The findings indicated that highly brominated PBDE homologues and highly chlorinated PCDD/F homologues were likely to be sorbed by SPM and marine floating MPs. The results of our study align with findings from previous research. Highly chlorinated or brominated homologues with highly hydrophobic characteristics are prevalent in SPM in seawater.^44,48,69−71^ Moreover, the strong hydrophobicity of MPs leads to the preferential sorption of organic pollutants with high *K*_OW_.^14,18,72,73^

**Figure 8:**
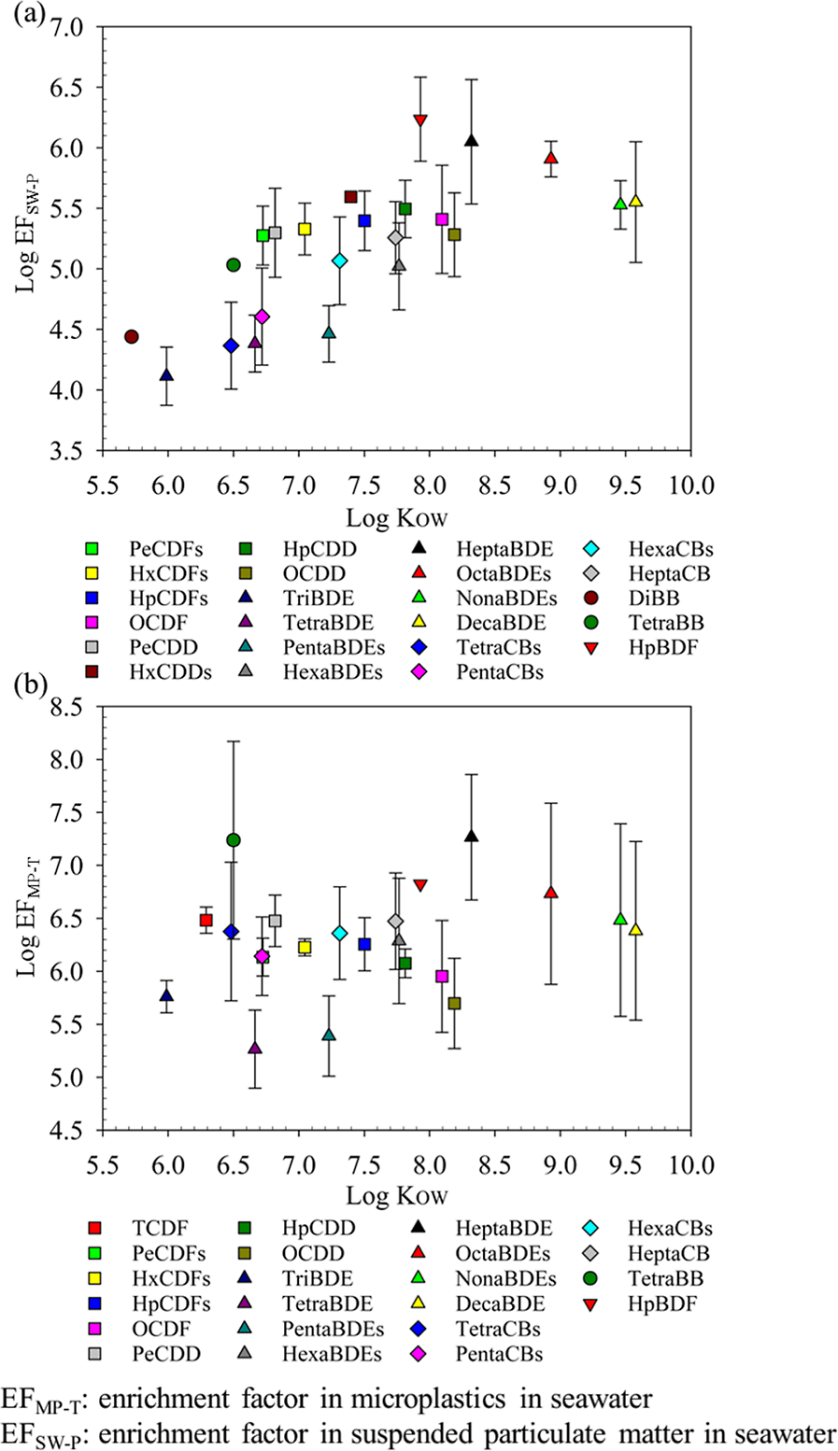
Relationships between *K*_OW_ and enrichment factor for POP homologues. (a) Log *K*_OW_ versus log EF_SW-P_, and (b) log *K*_OW_ versus log EF_MP-T_.

In this study, the distribution of enrichment factors and *K*_OW_ values for POP homologues is displayed in Figure 8. According to Spearman rank correlation analysis, the log *K*_OW_ and log EF_SW-P_ of POP homologues in SPM in seawater exhibited a significantly high correlation (*r*_s_ = 0.802, *p* < 0.01), as shown in Figure 8a. When the log *K*_OW_ was below 8.25, the log EF_SW-P_ tended to increase along with the rise in log *K*_OW_. This result indicated that SPM in seawater has a stronger adsorbing capacity for organic pollutants with higher hydrophobicity. This positive correlation was also observed between the organic carbon normalized partition coefficient (*K*_OC_) and *K*_OW_ in the sorption of organic pollutants.^74−76^ Therefore, these results indicated that hydrophobicity (*K*_ow_) can be utilized to estimate the distribution of POPs in SPM and dissolved POPs in seawater. Additionally, when the log *K*_OW_ exceeded 8.25, the log EF_SW-P_ of hepta- to deca-BDE homologues with higher molecular weight started to decrease (Figure 8a). A similar distribution trend was also observed in the sorption of MPs (Figure 8b). Apparently, for BDEs with higher hydrophobicity, log EF_SW-P_ or log EF_MP-T_ tended to decrease at a certain threshold of log *K*_OW_. This result was similar to the relationship between the logarithm of polydimethylsiloxane (PDMS)–water partition coefficient and *K*_OW_.^77−80^ Bao et al. (2011) and Qiu et al. (2021) indicated that there was a downward trend in the PE–water partition coefficients of PBDE congeners or novel halogenated flame retardants when log *K*_OW_ approximately exceeded 8.^79,80^ For hydrophobic organic pollutants, pollutants with stronger hydrophobicity (higher *K*_OW_) were characterized by larger molecules and required more Gibbs free energy to penetrate the internal structure of polymers.^80^

In floating MPs, when the log *K*_OW_ ranged from 5.5 to 8.25, the log EF_MP-T_ was observed to fall within the range of approximately 5 to 7 without exhibiting any increasing or decreasing trend (Figure 8b). Evidently, there were differences in the sorption characteristics of MPs and SPM on POPs when the *K*_OW_ values of POP congeners were below 8.25. This difference could be ascribed to the heterogeneity in the composition and structure of MPs, including the material difference, crystallinity of plastic, and aging.^52,53,58^ In general, PE, characterized by its expanded and flexible structure, is often conducive to the sorption of hydrophobic organic pollutants because of its high *K*_d_ and low desorption hysteresis.^81,82^ In the comprehensive analysis of various organic pollutants, the sorption capacity of PE is significantly higher than that of PS and PVC.^61^ Moreover, the crystallinity of plastics significantly influences their crystalline region and diffusion properties.^83^ Under the low concentration of organic contaminants, the sorption of MPs may predominantly occur in the crystalline region.^84^ Plastics with high crystallinity, such as PS, were not conducive to the adsorption of POPs in the environment.^32^ Most research on the sorption of organic pollutants was conducted in the laboratory using controlled uniform MP particles, whereas the mixed MP samples from the field environment in this study comprised PE, PP, and PS (as shown in Figure 3), predominantly in the form of irregular fragmented shapes. The structure of aged MPs in the field environment determined the level of heterogeneity and affected the sorption of organic pollutants.^85,86^ Therefore, the heterogeneous MPs in this study may contribute to the absence of a clear correlation (*r*_s_ = 0.244, *p* = 0.273) between the log *K*_OW_ and log EF_MP-T_ of POPs. However, the log EF_MP-T_ for PeCDD (highly toxic 1,2,3,7,8-PeCDD) in MP was 1–1.5 orders of magnitude higher than the log EF_SW-P_ (Figure 8). This characteristic may indicate that MPs were more prone to sorbing highly toxic POPs than suspended solids.

## Supporting Information Available

Sampling method for persistent organic pollutants in seawater, method for analyzing POPs, congener of POPs, *n*-octanol–water partition coefficient, elemental analysis of suspended particulate matter, concentration of POPs in the virgin microplastic pellets, data on enrichment factors and the concentration percentage of both PCDD/F and PBDE homologues, estimated POPs, and estimated enrichment factor for POPs in organic matter of SPM (PDF)
