# Supplementary material for: Enrichment of Persistent Organic Pollutants in Microplastics from Coastal Waters
Source: Environ Sci Technol. 2024 Dec 4;58(50):22391–404. doi: 10.1021/acs.est.4c10835 (PMC11656714; doi:10.1021/acs.est.4c10835)
Supplement: Supplementary file 1 — es4c10835_si_001.pdf [file es4c10835_si_001.pdf]

Enrichment of persistent organic pollutants (POPs) in microplastics from coastal waters

Supplementary Information

Lin-Chi Wang [a], Justin Chun-Te Lin [b], Jia-An Ye [c], Yee Cheng Lim [a], Chiu-Wen Chen [a], Cheng-Di Dong [a], Ta-Kang Liu [*, c]

[a] Department of Marine Environmental Engineering, National Kaohsiung University of Science and Technology, Kaohsiung City, 811213, Taiwan

[b] Department of Environmental Engineering and Science, Feng Chia University, Taichung City, 407102, Taiwan

[c] Institute of Ocean Technology and Marine Affairs, National Cheng Kung University, 1 University Road, Tainan City, 701401, Taiwan

[*] Corresponding author Tel: +886-6-2757575 Ext. 63283; Fax: +886-6-2753364

Email address: tkliu@mail.ncku.edu.tw (T.K. Liu)

Contents

S1. Sampling method for persistent organic pollutants (POPs) in seawater..................5
S2. Method for analyzing POPs...6
S2.1. Analytical procedures of POPs in seawater...6
S2.2. Analytical procedures of POPs in microplastics (MPs)..............................8
S2.3. Instrumental analysis of POPs 10
S2.4. Quality assurance and quality control (QA/QC).......................................10
S3. Congener of POPs...15
S3.1. POPs in seawater...15
S3.2. POPs in microplastics 26
S4. The n-octanol-water partition coefficient (K_{OW}) 35
S5. The elemental analysis of suspended particulate matter (SPM) 44
S6. The concentration of POPs in the virgin microplastic pellets...............................45
S7. The data on EF_{SW-P}, EF_{MP-T}, and the concentration percentage (%) of both PCDD/F and PBDE homologues 46
S8. The estimated POPs and the estimated enrichment factor for POPs in organic matter of SPM 50

Table and Figure of Contents

Fig. S1. Diagram of an automatic pre-concentration equipment revised from the NIEA W790.51B by Taiwan National Environmental Research Academy 5
Table S1. The targeted chemicals of the POPs 6
Table S2. The internal standards and cleanup standard of POPs 7
Table S3. The extraction methods and solvents used for different pollutants in microplastic samples. ... 9
Table S4. Recovery of standards and their corresponding criteria for analyzing POPs in SPM (filter samples) 11
Table S5. Recovery of standards and their corresponding criteria for analyzing dissolved POPs in seawater (PUF samples) 12
Table S6. Recovery of standards and their corresponding criteria for analyzing POPs in microplastics (MPs) 13
Table S7. Blank test of POP analysis 14
Table S8. The data on congeners of PCDD/Fs in SPM in seawater 15
Table S9. The data on congeners of PBDD/Fs in SPM in seawater 16
Table S10. The data on congeners of PBDEs in SPM in seawater 16
Table S11. The data on congeners of dioxin-like PCBs in SPM in seawater 17
Table S12. The data on congeners of PBBs in SPM in seawater 17
Table S13. The data on congeners of PCDD/Fs in SPM in seawater (unit: $pg \cdot g^{-1}$) 18
Table S14. The data on congeners of PBDD/Fs in SPM in seawater (unit: $pg \cdot g^{-1}$) 19
Table S15. The data on congeners of PBDEs in SPM in seawater (unit: $pg \cdot g^{-1}$) 19
Table S16. The data on congeners of dioxin-like PCBs in SPM in seawater (unit: $pg \cdot g^{-1}$) 20
Table S17. The data on congeners of PBBs in SPM in seawater (unit: $pg \cdot g^{-1}$) 20
Table S18. The data on congeners of dissolved PCDD/Fs in seawater 21
Table S19. The data on congeners of dissolved PBDD/Fs in seawater 21
Table S20. The data on congeners of dissolved PBDEs in seawater 22
Table S21. The data on congeners of dissolved dioxin-like PCB in seawater 22
Table S22. The data on congeners of dissolved PBBs in seawater 23
Fig. S2. Concentration percentages of individual congeners of POPs in SPM ($C_{SW\text{-}P}$, in the left column) and dissolved POPs ($C_{SW\text{-}D}$, in the right column) in seawater. (a, b) Σ_{17} PCDD/Fs, (c, d) Σ_{14} PBDEs, and (e, f) Σ_{12} dioxin-like PCBs. 25
Table S23. The data on congeners of PCDD/Fs in the surficial adsorption within MPs 26
Table S24. The data on congeners of PBDD/Fs in the surficial adsorption within MPs 27
Table S25. The data on congeners of PBDEs in the surficial adsorption within MPs 27
Table S26. The data on congeners of dioxin-like PCBs in the surficial adsorption within MPs 28
Table S27. The data on congeners of PBBs in the surficial adsorption within MPs 28
Table S28. The data on congeners of PCDD/Fs in the total sorption within MPs 29
Table S29. The data on congeners of PBDD/Fs in the total sorption within MPs 29

Table S30. The data on congeners of PBDEs in the total sorption within MPs 30
Table S31. The data on congeners of dioxin-like PCBs in the total sorption within MPs 30
Table S32. The data on congeners of PBBs in the total sorption within MPs..31
Fig. S3. Concentration percentages of individual congeners in the total sorption within floating MPs (C_{MP-T}). (a) Σ_{17} PCDD/Fs, (b) Σ_{12} PBDD/Fs, (c) Σ_{14} PBDEs, (d) Σ_{12} dioxin-like PCBs, and (e) Σ_5 PBBs. 33
Fig. S4. Concentration percentages of individual congeners in the surficial adsorption within floating MPs (C_{MP-S}). (a) Σ_{17} PCDD/Fs, (b) Σ_{12} PBDD/Fs, (c) Σ_{14} PBDEs, (d) Σ_{12} dioxin-like PCBs, and (e) Σ_5 PBBs..34
Table S33. Average of logarithm of K_{OW} of POP homologues..35
Table S34. K_{OW} of PCDD/F congeners 36
Table S35. K_{OW} of PBDD/F congeners 38
Table S36. K_{OW} of PBDE congeners 39
Table S37. K_{OW} of dioxin-like PCB congeners 42
Table S38. K_{OW} of PBB congeners..43
Table S39. Elemental component of SPM...44
Table S40. The proportion of fixed solids and volatile solids in SPM..44
Table S41. The concentrations of POPs in the virgin LDPE pellets 45
Table S42. The data on logarithm of EF_{SW-P} of POPs and TEQ-POPs..46
Table S43. The data on logarithm of EF_{MP-T} of POPs and TEQ-POPs 46
Table S44. The data on the concentration percentage (%) of PCDD/F homologues in SPM in seawater 46
Table S45. The data on the concentration percentage (%) of PBDE homologues in SPM in seawater..47
Table S46. The data on the concentration percentage (%) of PCDD/F homologues in the floating MPs 47
Table S47. The data on the total concentration percentage (%) of PBDE homologues in the floating MPs 47
Table S48. The data on logarithm of EF_{SW-P} of POP homologues in SPM in seawater 48
Table S49. The data on logarithm of EF_{MP-T} of POP homologues in the floating MPs..........................49
Table S50. The estimated PCDD/Fs in organic matter of SPM in seawater 50
Table S51. The estimated PBDD/Fs in organic matter of SPM in seawater 51
Table S52. The estimated PBDEs in organic matter of SPM in seawater...51
Table S53. The estimated dioxin-like PCBs in organic matter of SPM in seawater...............................52
Table S54. The estimated PBBs in organic matter of SPM in seawater..52
Table S55. The estimated logarithm of $EF_{Organic}$ of POPs and TEQ-POPs...52

S1. Sampling method for persistent organic pollutants (POPs) in seawater

An automatic pre-concentration equipment for large volume water (PCELVW) is used to collect a sufficient volume of water at a flow rate of approximately 1 liter per minute. Suspended particulate matter (SPM) and dissolved POPs in the water are captured using glass fiber filters and polyurethane foam (PUF). The PCELVW consists of a glass fiber filter holder, a polyurethane foam holder (PUF holder), a vacuum pressure sensor, a pump, a stainless-steel container, liquid level sensor, a flow meter, and a computer controller, as shown in Fig. S1. During sampling, a glass fiber filter (Advantec GC-50, diameter: 142 mm, pore size: 0.5 µm, Advantec Co., Tokyo, Japan) and PUF (TISCH TE1010, diameter: 63 mm, height: 3-inch, density more than 0.022 g/cm^{-3}, Tisch Environmental Inc., OH, USA) is placed into a glass fiber filter holder and PUF holder, respectively.[1,2] The PUF can absorb a sufficient quantity of dissolved POPs in water by passing a large volume of water through PCELVW.[1,2]

Additionally, before sampling, the blank filter and PUF were made by pre-processing to eliminate all organic contaminants. The filter and PUF is placed inside a Soxhlet extractor, where it is subjected to toluene reflux for more than four cycles per hour, with a total extraction time of four hours. After extraction, the filter and PUF is rinsed with acetone, and then placed in a vacuum oven to dry. The dried filter and PUF were sealed in clean glass jars for sampling use.[3,4] Additionally, to avoid external contamination and self-contamination during sampling and experimentation, all equipment, instruments, and containers were made from non-plastic materials. These items underwent a thorough cleaning process, which included soaking in a cleaning solution, ultrasonic cleansing, and a hot water rinse. Subsequently, they were sequentially rinsed with solvents like reagent water, acetone, and dichloromethane. After drying, the items were wrapped in aluminum foil for storage and future use.

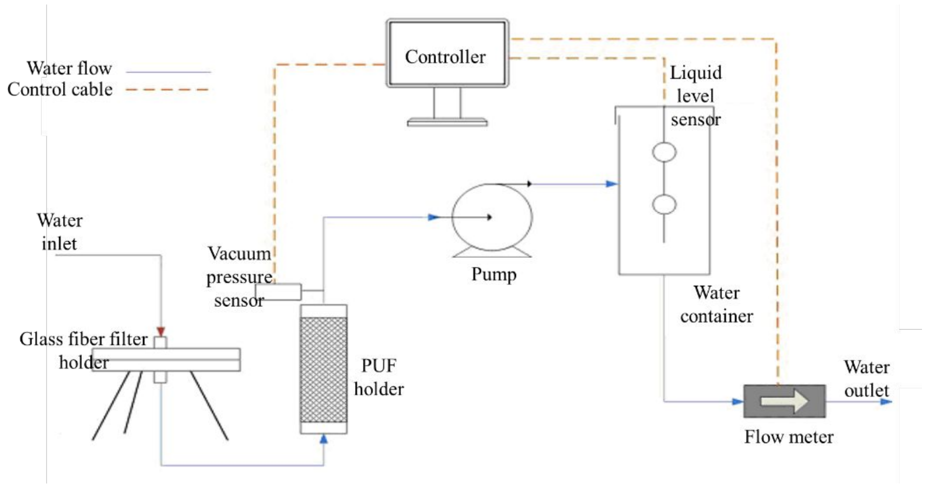

Fig. S1. Diagram of an automatic pre-concentration equipment revised from the NIEA W790.51B by Taiwan National Environmental Research Academy

S2. Method for analyzing POPs

S2.1. Analytical procedures of POPs in seawater

The determination of POPs in seawater referred to the method of NIEA M801.13B, M802.00B, and M803.00B by the Taiwan National Environmental Research Academy, the Ministry of Environment which were modified from US EPA M1613B, M8290A, M1614, and M1668, respectively.[1,2] All the chemical analyses for POPs in this study were carried out in the accredited laboratory certified by the Ministry of Environment, Taiwan. The study examined five types of POPs, i.e., PCDD/Fs, PBDD/Fs, PBDEs, dioxin-like PCBs, and PBBs. The congeners analyzed were detailed in Table S1. The internal standards and cleanup standard in Table S2 were included in the samples before extraction. The standards for quantification and identification of known substances were purchased from Cambridge Isotope Laboratories Inc. (Andover, MA, USA) or Wellington Laboratories Inc. (Ontario, Canada).

Table S1. The targeted chemicals of the POPs

POPs	Congeners
PCDD/Fs	17 species: 7 dioxins (i.e., 2,3,7,8-TeCDD 、 1,2,3,7,8-PeCDD, 1,2,3,4,7,8-HxCDD, 1,2,3,6,7,8-HxCDD, 1,2,3,7,8,9-HxCDD, 1,2,3,4,6,7,8-HpCDD and OCDD); 10 furans (i.e., 2,3,7,8-TeCDF, 1,2,3,7,8-PeCDF, 2,3,4,7,8-PeCDF, 1,2,3,4,7,8-HxCDF, 1,2,3,6,7,8-HxCDF, 2,3,4,6,7,8-HxCDF, 1,2,3,7,8,9-HxCDF, 1,2,3,4,6,7,8-HpCDF, 1,2,3,4,7,8,9-HpCDF and OCDF).
dioxin-like PCBs	12 species: 2 tetra-chlorinated (PCB 77, PCB 81), 5 penta-chlorinated (PCB 105, PCB 114, PCB 118, PCB 123 and PCB 126), 4 hexa-chlorinated (PCB 156, PCB 157, PCB 167 and PCB 169) and 1 hepta-chlorinated (PCB 189) biphenyl.
PBDD/Fs	12 species: 6 dioxins (i.e. 2,3,7,8,-TeBDD, 1,2,3,7,8-PeBDD, 1,2,3,4/6,7,8-HxBDD, 1,2,3,7,8,9-HxBDD, 1,2,3,4,6,7,8-HpBDD and OctBDD); 6 furans (i.e. 2,3,7,8-TeBDF, 1,2,3,7,8-PeBDF, 2,3,4,7,8-PeBDF, 1,2,3,4,7,8-HxBDF, 1,2,3,4,6,7,8-HpBDF and OctBDF)
PBBs	5 species: PBB 15 (4,4'-diBB), PBB 52 (2,2',5,5'-tetraBB), PBB 153 (2,2',4,4',5,5'-hexaBB), PBB 180 (2,2',3,4,4',5,5'-heptaBB), PBB 194 (2,2',3,3',4,4',5,5'-octaBB)
PBDEs	14 species: 1 tri-brominated (BDE 28), 1 tetra-brominated (BDE 47), 2 penta-brominated (BDE 99 and BDE 100), 2 hexa-brominated (BDE 153 and BDE 154), 1 hepta-brominated (BDE 183), 3 octa-brominated (BDE 196, BDE 197 and BDE 203), 3 nona-brominated (BDE 206, BDE 207 and BDE 208), and 1 deca-brominated (BDE 209) diphenyl ether.

Table S2. The internal standards and cleanup standard of POPs

Analytes	Internal standards *(CAS Number or Cambridge Isotope Laboratories product code)*
PCDD/Fs	$^{13}C_{12}$-2,3,7,8-TeCDD *(76523-40-5)*, $^{13}C_{12}$-1,2,3,7,8-PeCDD *(109719-79-1)*, $^{13}C_{12}$-1,2,3,4,7,8-HxCDD *(109719-80-4)*, $^{13}C_{12}$-1,2,3,6,7,8-HxCDD *(109719-81-5)*, $^{13}C_{12}$-1,2,3,4,6,7,8-HpCDD *(109719-83-7)*, $^{13}C_{12}$-OCDD *(114423-97-1)*, $^{13}C_{12}$-2,3,7,8-TeCDF *(89059-46-1)*, $^{13}C_{12}$-1,2,3,7,8-PeCDF *(109719-77-9)*, $^{13}C_{12}$-2,3,4,7,8-PeCDF *(116843-02-8)*, $^{13}C_{12}$-1,2,3,4,7,8-HxCDF *(114423-98-2)*, $^{13}C_{12}$-1,2,3,6,7,8-HxCDF *(116843-03-9)*, $^{13}C_{12}$-2,3,4,6,7,8-HxCDF *(116843-05-1)*, $^{13}C_{12}$-1,2,3,7,8,9-HxCDF *(116843-04-0)*, $^{13}C_{12}$-1,2,3,4,6,7,8-HpCDF *(109719-84-8)*, and $^{13}C_{12}$-1,2,3,4,7,8,9-HpCDF *(109719-94-0)*
PCBs	$^{13}C_{12}$-3,4,4',5-TeCB *(208461-24-9)*, $^{13}C_{12}$-3,3',4,4'-TeCB *(105600-23-5)*, $^{13}C_{12}$-2,3,3',4,4'-PeCB *(208263-62-1)*, $^{13}C_{12}$-2,3,4,4',5-PeCB *(208263-63-2)*, $^{13}C_{12}$-2,3',4,4',5-PeCB *(104130-40-7)*, $^{13}C_{12}$-2',3,4,4',5-PeCB *(208263-64-3)*, $^{13}C_{12}$-3,3',4,4',5-PeCB *(208263-65-4)*, $^{13}C_{12}$-2,3,3',4,4',5-HxCB *(208263-68-7)*, $^{13}C_{12}$-2,3,3',4,4',5'-HxCB *(235416-30-5)*, $^{13}C_{12}$-2,3',4,4',5,5'-HxCB *(208263-69-8)*, $^{13}C_{12}$-3,3',4,4',5,5'-HxCB *(208263-70-1)*, and $^{13}C_{12}$-2,3,3',4,4',5,5'-HpCB *(208263-73-4)*
PBDD/Fs	$^{13}C_{12}$-2,3,7,8-TeBDD *(125749-35-1)*, $^{13}C_{12}$-1,2,3,7,8-PeBDD *(161880-52-0)*, $^{13}C_{12}$-1,2,3,4/6,7,8-HxBDD *(CIL-ED-2534/5237-A)*, $^{13}C_{12}$-1,2,3,7,8,9-HxBDD *(1265827-76-6)*, $^{13}C_{12}$-1,2,3,4,6,7,8-HpBDD *(CIL-ED-5357)*, $^{13}C_{12}$-OctBDD *(161880-56-4)*, $^{13}C_{12}$-2,3,7,8-TeBDF *(161880-53-1)*, $^{13}C_{12}$-1,2,3,7,8-PeBDF *(161880-54-2)*, $^{13}C_{12}$-2,3,4,7,8-PeBDF *(1265827-80-2)*, $^{13}C_{12}$-1,2,3,4,7,8-HxBDF *(1265827-82-4)*, $^{13}C_{12}$-1,2,3,4,6,7,8-HpBDF *(161880-55-3)*, and $^{13}C_{12}$-OctBDF *(CIL-EF-5266)*
PBBs	$^{13}C_{12}$-PBB#52(4Br) *(1401248-99-4)*, $^{13}C_{12}$-PBB#153(6Br) *(1401249-00-0)*, and $^{13}C_{12}$-PBB#194(8Br) *(1401249-01-1)*
PBDEs	$^{13}C_{12}$-BDE#28(3Br) *(488710-19-6)*, $^{13}C_{12}$-BDE#47(4Br) *(488710-20-9)*, $^{13}C_{12}$-BDE#99(5Br) *(488710-21-0)*, $^{13}C_{12}$-BDE#154(6Br) *(488710-23-2)*, $^{13}C_{12}$-BDE#153(6Br) *(488710-22-1)*, $^{13}C_{12}$-BDE#183(7Br) *(488710-24-3)*, $^{13}C_{12}$-BDE#197(8Br) *(1367487-31-7)*, $^{13}C_{12}$-BDE#207(9Br) *(1367487-33-9)*, and $^{13}C_{12}$-BDE#209(10Br) *(562099-68-7)*
**Analytes**	**Cleanup Standard** *(CAS Number or Cambridge Isotope Laboratories product code)*
PCDD/Fs	$^{37}Cl_{4}$-2,3,7,8-TeCDD *(85508-50-5)*

S2.2. Analytical procedures of POPs in microplastics (MPs)

Currently, there are no uniform procedures for determining POPs in microplastics. Table S1 provides a compilation of the typical extraction methods and solvents utilized for various contaminants in microplastic samples. In Table S3, previous research has frequently utilized methods such as maceration, solvent-rinsed tweezers, sonication accelerated extraction, microwave accelerated extraction, and soxhlet extraction using various solvents. Some studies utilized dichloromethane (DCM), tetrahydrofuran and m-cresol to dissolve plastics for analyzing organic pollutants. Therefore, in this study, we opted to use ultrasonic extraction (sonication accelerated extraction) with n-hexane to extract surficial POPs in microplastic samples. To extract the inner POPs in microplastic samples without damaging the instrument, we also used ultrasonic extraction instead of Soxhlet extraction. The same microplastic samples were then subjected to sonication with tetrahydrofuran, n-hexane, and toluene in succession.

The internal standards in Table S2 were included in the samples before extraction. The concentrated extracts were then treated with concentrated sulfuric acid, followed by a sequence of cleanup and fractionation steps using multi-layered silica, alumina, and activated carbon columns. In brief, while performing the alumina column cleanup, non- planar PCBs/PBBs were eluted using 15 mL of n-hexane and 25 mL of DCM/n-hexane (4/96, v/v), followed by an additional elution with 25 mL of DCM/n-hexane (40/60, v/v) for activated carbon column. The activated carbon column was eluted with 5 mL of toluene/methanol/ethyl acetate/hexane (5/5/10/80, v/v) to extract planar PCBs/PBBs and, PBDEs. This was then followed by an additional elution with 40 mL of toluene to extract PCDD/Fs and PBDD/Fs. Prior to conducting instrumental analysis, the eluates of planar and non-planar PCBs/PBBs were combined to represent the samples of PCBs and PBBs. The eluate was concentrated to 1 mL and then transferred to a vial. Subsequently, it was further concentrated to almost complete dryness using a stream of nitrogen. Prior to injection, 10 μL of the standard solution for recovery checking was added to the sample extract to minimize potential loss. The detailed description of the analytical procedures can be found in our previous publications (Wang et al., 2010; Chang et al., 2014; Chao et al., 2014; Mwangi et al., 2016; Wang et al., 2021; Ye et al., 2024).[19,20,21,22,23,24]

Table S3. The extraction methods and solvents used for different pollutants in microplastic samples.

Plastic types	Extraction method (solvent)	Analytes	References
Marine debris	Sonication accelerated extraction (hexane)	Phthalates, BFR, benzotriazoles	(Cormier et al., 2021)[5]
Marine debris	Sonication accelerated extraction (toluene)	Phthalates, BPA derivatives,benzotriazoles, phenolicantioxidants, NIAS	(Llorca et al., 2021)[6]
Recycled product	Sonication accelerated extraction (toluene)	PBDEs	(Sun et al., 2021)[7]
Marine plastic debris	Dissolution and precipitation (dichloromethane)	Hexabromocyclododecane	(Aminot et al., 2020)[8]
marine debris	Maceration (hexane)	Benzotriazoles, benzophenone, BFR, NIAS	(Tanaka et al., 2019)[9]
Styrene oligomers	Dissolution and precipitation (dichloromethane)	NIAS	(Tian et al., 2020)[10]
microplastic materials	Sonication accelerated extraction (acetonitrile, tetrahydrofuran/propan-2-ol)	Phthalates, BPA	(Fikarová et al., 2019)[11]
recycled product (<5 mm)	Microwave accelerated extraction (toluene/Acetone,	PBDEs, BTBPE	(Sun et al., 2019)[12]
new product (PE, PS, PET, PVC)	Dissolution and precipitation (tetrahydrofuran, dichloromethane, xylol, m-cresol)	Phthalates, organophosphites, NIAS	(Suhrhoff and Scholz-Böttcher, 2016)[13]
Marine plastic debris	Soxhlet extraction (dichloromethane)	BFR, BPA, alkyl phenols	(Hirai et al., 2011)[14]
Plastic particle	Sonication accelerated extraction (hexane)	PCBs	(Syberg et al., 2020)[15]
Plastic resin pellets	solvent-rinsed tweezers (acetone, dichloromethane and n-hexane)	Organochlorine Pesticides	(Pflieger et al., 2017)[16]
Plastic debris	Sonication accelerated extraction (acetone/hexane, 1:1)	PAHs, PCBs, DDT and chlordanes	(Van et al., 2012)[17]
Plastic resin pellets	Maceration (hexane)	n-alkanes, hopanes, PCBs, DDT, DDD, 4 HCH isomers, PAHs	(Heskett et al., 2012)[18]

S2.3. Instrumental analysis of POPs

A high-resolution gas chromatograph/high-resolution mass spectrometer (HRGC/HRMS) is utilized to conduct the analysis of POPs. The HRGC (Hewlett–Packard 6970 Series gas, CA) is eaaquipped with a silica capillary column (J&W Scientific, CA) and a splitless injector. The HRMS (Micromass Autospec Ultima, Manchester, UK) features a positive electron impact (EI+) source. The selected ion monitoring (SIM) mode with a resolving power of 10,000 is employed for analysis. The electron energy and source temperature are set at 35 eV and 250 °C, respectively. Each analyte necessitates separate injections, meaning that five injections are needed for analyzing PCDD/Fs, PBDD/Fs, PBDEs, dioxin-like PCBs, and PBBs. Further details on the instrumental analysis parameters can be found in our previous publications.[19,20,21,22,23,24]

S2.4. Quality assurance and quality control (QA/QC)

The methodological quality assurance and quality control (QA/QC) employed in this study adhered to the standards set by the Taiwan National Environmental Research Academy's method NIEA M801.13B, M802.00B, and M803.00B, as well as the US EPA's method M1613B, M8290A, M1614, and M1668. The limits of detection (LOD) were defined as the ratio of signal to noise (S/N) over 3, while the limit of quantification (LOQ) was defined as the ratio of S/N over 10. The method detection limits (MDLs) were determined to be 2.5–5.0 times the estimated LOD. The mass ratios of at least two characteristic ions agreed with the theoretical values to within a deviation of 15%.

Prior to the extraction process, the $^{13}C_{12}$-labeled internal standards were added to the samples to enable the quantitative assessment of POPs during the analytical procedures. Internal standards were employed to monitor the performance of the extraction and cleanup. Moreover, the recovery and reproducibility of the analysis were evaluated by spiking blank samples with a known amount of the targeted analyte. The precision and recovery standard (PAR), internal standards, and cleanup standard of the POPs all satisfied the applicable criteria (Tables S4, S5, and S6). Moreover, the blank test was systematically examined in every batch of the analyses, and table S7 showed the result of blank test for POP analysis.

Table S4. Recovery of standards and their corresponding criteria for analyzing POPs in SPM (filter samples)

Standard	Analytes	Homologue	Recovery (%)	Criteria (%)
PAR	PCDD/Fs	All	89.1–113	70–130
	PBDD/Fs	tetra through hepta	86.9–100	60–140
		Octa	90.8–91.9	50–150
	PBDEs	All	85.2–122	60–140
	dioxin-like PCBs	All	82.4–121	60–140
	PBBs	Di	85.9	50–150
		tetra through octa	71.3–76.8	60–140
Cleanup Standard	PCDD/Fs	$^{37}Cl_4$-2,3,7,8-TeCDD	44.9–60.3	30–130
Internal Standards ($^{13}C_{12}$-labeled)	PCDD/Fs	tetra and penta	31.2–68.9	30–130
		hexa through octa	41.4–63.6	40–130
	PBDD/Fs	tetra through hepta	21.4–136	20–150
		Octa	106–147	10–150
	PBDEs	BDE-28, 47, 99, 154, 153,183, 197, 207, 209	26.1–111	20–150
	dioxin-like PCBs	All	37.1–123	25–150

Table S5. Recovery of standards and their corresponding criteria for analyzing dissolved POPs in seawater (PUF samples)

Standard	Analytes	Homologue	Recovery (%)	Criteria (%)
PAR	PCDD/Fs	All	89.1-113	70–130
	PBDD/Fs	tetra through hepta	86.9–100	60–140
		Octa	90.8–91.9	50–150
	PBDEs	All	85.2–122	60–140
	dioxin-like PCBs	All	82.4–121	60–140
	PBBs	Di	85.9	50–150
		tetra through octa	71.3–76.8	60–140
Cleanup Standard	PCDD/Fs	$^{37}Cl_4$-2,3,7,8-TeCDD	45.2–60.5	30–130
Internal Standards ($^{13}C_{12}$-labeled)	PCDD/Fs	tetra and penta	30.5–61.4	30–130
		hexa through octa	42.6–58.5	40–130
	PBDD/Fs	tetra through hepta	21.8–129	20–150
		Octa	124–147	10–150
	PBDEs	BDE-28, 47, 99, 154, 153,183, 197, 207, 209	27.3–96.0	20–150
	dioxin-like PCBs	All	32.7–102	25–150

Table S6. Recovery of standards and their corresponding criteria for analyzing POPs in microplastics (MPs)

Standard	Analytes	Homologue	surficial adsorption	total sorption	Criteria (%)
			Recovery (%)	Recovery (%)	
PAR	PCDD/Fs	All	98.3–127	98.3–127	70–130
	PBDD/Fs	tetra through hepta	111–124	111–124	60–140
		Octa	116–124	116–124	50–150
	PBDEs	All	85.0–127	85.0–127	60–140
	dioxin-like PCBs	All	94.0–127	94.0–127	60–140
	PBBs	Di	105	105	50–150
		tetra through octa	85.5–95.3	85.5–95.3	60–140
Cleanup Standard	PCDD/Fs	$^{37}C_{14}$-2,3,7,8-TeCDD	61.3–89.6	76.7–91.1	30–130
Internal Standards ($^{13}C_{12}$-labeled)	PCDD/Fs	tetra and penta	42.2–89.1	50.9–102	30–130
		hexa through octa	61.5–108	50.1–108	40–130
	PBDD/Fs	tetra through hepta	41.0–109	42.9–114	20–150
		Octa	55.0–122	59.1–116	10–150
	PBDEs	BDE-28, 47, 99, 154, 153,183, 197, 207, 209	20.7–80.2	20.7–110	20–150
	dioxin-like PCBs	All	38.6–90.7	31.3–92.6	25–150

Table S7. Blank test of POP analysis

(a) PCDD/Fs	Concentration ($pg \cdot g^{-1}$)
2,3,7,8-TeCDF	N.D.
1,2,3,7,8-PeCDF	N.D.
2,3,4,7,8-PeCDF	N.D.
1,2,3,4,7,8-HxCDF	N.D.
1,2,3,6,7,8-HxCDF	N.D.
2,3,4,6,7,8-HxCDF	N.D.
1,2,3,7,8,9-HxCDF	N.D.
1,2,3,4,6,7,8-HpCDF	N.D.
1,2,3,4,7,8,9-HpCDF	N.D.
OCDF	0.221
2,3,7,8-TeCDD	N.D.
1,2,3,7,8-PeCDD	N.D.
1,2,3,4,7,8-HxCDD	N.D.
1,2,3,6,7,8-HxCDD	N.D.
1,2,3,7,8,9-HxCDD	N.D.
1,2,3,4,6,7,8-HpCDD	N.D.
OCDD	0.237
**Σ_{17} PCDD/Fs**	**0.458**

(c) PBDEs	Concentration ($pg \cdot g^{-1}$)
BDE #28(3Br)	0.230
BDE #47(4Br)	0.291
BDE #100(5Br)	0.328
BDE #99(5Br)	0.527
BDE #154(6Br)	0.286
BDE #153(6Br)	0.601
BDE #183(7Br)	0.807
BDE #197(8Br)	2.85
BDE #203(8Br)	4.39
BDE #196(8Br)	4.33
BDE #208(9Br)	17.2
BDE #207(9Br)	20.0
BDE #206(9Br)	10.6
BDE #209(10Br)	133
**Σ_{14} PBDEs**	**195**

(b) PBDD/Fs	Concentration ($pg \cdot g^{-1}$)
2,3,7,8-TeBDF	N.D.
1,2,3,7,8-PeBDF	N.D.
2,3,4,7,8-PeBDF	N.D.
1,2,3,4,7,8-HxBDF	N.D.
1,2,3,4,6,7,8-HpBDF	N.D.
OctBDF	N.D.
2,3,7,8,-TeBDD	N.D.
1,2,3,7,8-PeBDD	N.D.
1,2,3,4/6,7,8-HxBDD	N.D.
1,2,3,7,8,9-HxBDD	N.D.
1,2,3,4,6,7,8-HpBDD	N.D.
OctBDD	N.D.
**Σ_{12} PBDD/Fs**	**N.D.**

(d) dioxin-like PCBs	Concentration ($pg \cdot g^{-1}$)
PCB#77(4CL)	0.440
PCB#81(4CL)	N.D.
PCB#105(5CL)	0.623
PCB#114(5CL)	N.D.
PCB#118(5CL)	0.586
PCB#123(5CL)	N.D.
PCB#126(5CL)	N.D.
PCB#156(6CL)	N.D.
PCB#157(6CL)	N.D.
PCB#167(6CL)	N.D.
PCB#169(6CL)	N.D.
PCB#189(7CL)	N.D.
**Σ_{12} dioxin-like PCB**	**1.65**

(e) PBBs	Concentration ($pg \cdot g^{-1}$)
PBB#15(2Br)	N.D.
PBB#52(4Br)	N.D.
PBB#153(6Br)	N.D.
PBB#180(7Br)	N.D.
PBB#194(8Br)	N.D.
**Σ_5 PBB**	**N.D.**

S3. Congener of POPs

S3.1. POPs in seawater

A total of five types of POPs, including PCDD/Fs, PBDD/Fs, PBDEs, dioxin-like PCBs, and PBBs, were analyzed. Tables S8–S17 displayed the data on congeners of POPs in suspended particulate matter (SPM) in seawater, while Tables S18–S22 showed the data on congeners of dissolved POPs in seawater. The data in Tables S8–S12 and S18–S22 was calculated based on the seawater per unit volume, whereas the data in Tables S13–S17 in was calculated based on the SPM per unit weight. Furthermore, the characteristics of PCDD/F, PBDE, and dioxin-like PCB congeners in seawater were discussed in the following paragraphs.

Table S8. The data on congeners of PCDD/Fs in SPM in seawater

Concentration (unit: $pg \cdot L^{-1}$)	AP1	AP2	KH1	KH2
2,3,7,8-TeCDF	N.D.	N.D.	N.D.	0.00506
1,2,3,7,8-PeCDF	0.00332	0.00829	0.00282	0.00808
2,3,4,7,8-PeCDF	0.00472	0.00970	0.00383	0.00782
1,2,3,4,7,8-HxCDF	N.D.	0.00496	0.00513	0.00854
1,2,3,6,7,8-HxCDF	0.00333	0.00659	0.00356	0.00731
2,3,4,6,7,8-HxCDF	0.00493	0.00560	0.00368	0.00932
1,2,3,7,8,9-HxCDF	N.D.	N.D.	N.D.	N.D.
1,2,3,4,6,7,8-HpCDF	0.0148	0.0154	0.0171	0.0299
1,2,3,4,7,8,9-HpCDF	0.00368	0.00217	0.00247	0.00441
OCDF	0.0374	0.0313	0.0600	0.0534
2,3,7,8-TeCDD	N.D.	N.D.	N.D.	N.D.
1,2,3,7,8-PeCDD	0.00293	0.00238	0.00142	0.00338
1,2,3,4,7,8-HxCDD	N.D.	N.D.	N.D.	N.D.
1,2,3,6,7,8-HxCDD	0.00193	0.00301	N.D.	0.00257
1,2,3,7,8,9-HxCDD	N.D.	0.00275	0.00298	0.00388
1,2,3,4,6,7,8-HpCDD	0.0315	0.0201	0.0215	0.0231
OCDD	0.150	0.149	0.135	0.235
Σ17PCDD/Fs	0.259	0.262	0.259	0.402

Table S9. The data on congeners of PBDD/Fs in SPM in seawater

Concentration (unit: pg·L^{-1})	AP1	AP2	KH1	KH2
2,3,7,8-TeBDF	N.D.	N.D.	N.D.	N.D.
1,2,3,7,8-PeBDF	N.D.	N.D.	N.D.	N.D.
2,3,4,7,8-PeBDF	N.D.	N.D.	0.0108	N.D.
1,2,3,4,7,8-HxBDF	N.D.	N.D.	0.100	0.0392
1,2,3,4,6,7,8-HpBDF	0.102	0.134	0.921	0.306
OctBDF	0.140	0.172	0.895	0.504
2,3,7,8,-TeBDD	N.D.	N.D.	N.D.	N.D.
1,2,3,7,8-PeBDD	N.D.	N.D.	N.D.	N.D.
1,2,3,4/6,7,8-HxBDD	N.D.	N.D.	N.D.	N.D.
1,2,3,7,8,9-HxBDD	N.D.	N.D.	N.D.	N.D.
1,2,3,4,6,7,8-HpBDD	N.D.	N.D.	N.D.	N.D.
OctBDD	N.D.	N.D.	N.D.	N.D.
Σ12PBDD/Fs	0.242	0.306	1.93	0.849

Table S10. The data on congeners of PBDEs in SPM in seawater

Concentration (unit: pg·L^{-1})	AP1	AP2	KH1	KH2
BDE #28(3Br)	0.0245	0.109	0.0337	0.0247
BDE #47(4Br)	1.42	4.57	1.30	1.15
BDE #100(5Br)	0.352	0.804	0.198	0.212
BDE #99(5Br)	1.68	3.52	0.703	0.825
BDE #154(6Br)	0.269	0.413	0.569	0.175
BDE #153(6Br)	0.301	0.492	1.02	0.225
BDE #183(7Br)	0.642	1.16	2.86	1.07
BDE #197(8Br)	0.341	0.746	7.21	2.61
BDE #203(8Br)	0.581	1.66	17.1	6.47
BDE #196(8Br)	0.648	1.33	16.0	6.19
BDE #208(9Br)	0.751	1.68	18.6	12.0
BDE #207(9Br)	1.93	3.06	56.8	31.1
BDE #206(9Br)	2.11	3.52	55.9	28.2
BDE #209(10Br)	35.5	45.9	264	182
Σ14BDE	46.6	68.9	443	272

Table S11. The data on congeners of dioxin-like PCBs in SPM in seawater

Concentration (unit: $pg \cdot L^{-1}$)	AP1	AP2	KH1	KH2
PCB#77(4CL)	0.0295	0.166	0.0530	0.0378
PCB#81(4CL)	0.00137	0.00734	N.D.	N.D.
PCB#105(5CL)	0.0735	0.895	0.182	0.120
PCB#114(5CL)	0.00245	0.0411	0.0109	0.00478
PCB#118(5CL)	0.232	3.56	0.527	0.366
PCB#123(5CL)	0.00748	0.192	0.00954	0.00834
PCB#126(5CL)	0.00246	0.0226	0.00783	0.00612
PCB#156(6CL)	0.0475	1.05	0.120	0.0984
PCB#157(6CL)	0.00810	0.125	0.0214	0.0165
PCB#167(6CL)	0.0355	0.524	0.0840	0.0653
PCB#169(6CL)	N.D.	0.00292	N.D.	N.D.
PCB#189(7CL)	0.00760	0.156	0.0195	0.0215
Σ12PCB	0.447	6.74	1.04	0.745

Table S12. The data on congeners of PBBs in SPM in seawater

Concentration (unit: $pg \cdot L^{-1}$)	AP1	AP2	KH1	KH2
PBB#15(2Br)	0.00989	N.D.	N.D.	N.D.
PBB#52(4Br)	N.D.	0.00667	N.D.	N.D.
PBB#153(6Br)	N.D.	0.0571	N.D.	N.D.
PBB#180(7Br)	N.D.	N.D.	N.D.	N.D.
PBB#194(8Br)	N.D.	N.D.	N.D.	N.D.
Σ5PBB	0.00989	0.0638	N.D.	N.D.

Table S13. The data on congeners of PCDD/Fs in SPM in seawater (unit: pg·g^{-1})

Concentration (unit: pg·g^{-1})	AP1	AP2	KH1	KH2
2,3,7,8-TeCDF	N.D.	N.D.	N.D.	0.271
1,2,3,7,8-PeCDF	0.497	0.340	0.296	0.432
2,3,4,7,8-PeCDF	0.706	0.398	0.402	0.418
1,2,3,4,7,8-HxCDF	N.D.	0.203	0.539	0.457
1,2,3,6,7,8-HxCDF	0.498	0.270	0.373	0.391
2,3,4,6,7,8-HxCDF	0.737	0.229	0.386	0.498
1,2,3,7,8,9-HxCDF	N.D.	N.D.	N.D.	N.D.
1,2,3,4,6,7,8-HpCDF	2.22	0.629	1.79	1.60
1,2,3,4,7,8,9-HpCDF	0.549	0.0891	0.259	0.236
OCDF	5.59	1.28	6.30	2.85
2,3,7,8-TeCDD	N.D.	N.D.	N.D.	N.D.
1,2,3,7,8-PeCDD	0.438	0.0975	0.149	0.181
1,2,3,4,7,8-HxCDD	N.D.	N.D.	N.D.	N.D.
1,2,3,6,7,8-HxCDD	0.288	0.124	N.D.	0.137
1,2,3,7,8,9-HxCDD	N.D.	0.113	0.313	0.208
1,2,3,4,6,7,8-HpCDD	4.70	0.824	2.25	1.23
OCDD	22.5	6.12	14.2	12.6
Σ17PCDD/Fs	38.7	10.7	27.2	21.5

Table S14. The data on congeners of PBDD/Fs in SPM in seawater (unit: pg·g^{-1})

Concentration (unit: pg·g^{-1})	AP1	AP2	KH1	KH2
2,3,7,8-TeBDF	N.D.	N.D.	N.D.	N.D.
1,2,3,7,8-PeBDF	N.D.	N.D.	N.D.	N.D.
2,3,4,7,8-PeBDF	N.D.	N.D.	1.13	N.D.
1,2,3,4,7,8-HxBDF	N.D.	N.D.	10.5	2.10
1,2,3,4,6,7,8-HpBDF	15.2	5.50	96.7	16.3
OctBDF	21.0	7.07	94.0	27.0
2,3,7,8,-TeBDD	N.D.	N.D.	N.D.	N.D.
1,2,3,7,8-PeBDD	N.D.	N.D.	N.D.	N.D.
1,2,3,4/6,7,8-HxBDD	N.D.	N.D.	N.D.	N.D.
1,2,3,7,8,9-HxBDD	N.D.	N.D.	N.D.	N.D.
1,2,3,4,6,7,8-HpBDD	N.D.	N.D.	N.D.	N.D.
OctBDD	N.D.	N.D.	N.D.	N.D.
Σ12PBDD/Fs	36.2	12.6	202	45.4

Table S15. The data on congeners of PBDEs in SPM in seawater (unit: pg·g^{-1})

Concentration (unit: pg·g^{-1})	AP1	AP2	KH1	KH2
BDE #28(3Br)	3.66	4.45	3.54	1.32
BDE #47(4Br)	212	187	136	61.5
BDE #100(5Br)	52.6	32.9	20.8	11.4
BDE #99(5Br)	251	144	73.8	44.1
BDE #154(6Br)	40.2	16.9	59.7	9.34
BDE #153(6Br)	44.9	20.2	107	12.0
BDE #183(7Br)	95.9	47.4	300	57.2
BDE #197(8Br)	51.0	30.6	757	140
BDE #203(8Br)	86.8	68.2	1,800	346
BDE #196(8Br)	96.8	54.6	1,680	331
BDE #208(9Br)	112	68.8	1,950	640
BDE #207(9Br)	288	125	5,960	1,660
BDE #206(9Br)	315	144	5,860	1,510
BDE #209(10Br)	5,310	1,880	27,700	9,730
Σ14BDE	6,960	2,830	46,400	14,600

Table S16. The data on congeners of dioxin-like PCBs in SPM in seawater (unit: $pg \cdot g^{-1}$)

Concentration (unit: $pg \cdot g^{-1}$)	AP1	AP2	KH1	KH2
PCB#77(4CL)	4.41	6.79	5.56	2.02
PCB#81(4CL)	0.205	0.301	N.D.	N.D.
PCB#105(5CL)	11.0	36.7	19.1	6.40
PCB#114(5CL)	0.366	1.69	1.14	0.256
PCB#118(5CL)	34.6	146	55.3	19.6
PCB#123(5CL)	1.12	7.88	1.00	0.446
PCB#126(5CL)	0.368	0.926	0.822	0.327
PCB#156(6CL)	7.09	43.2	12.6	5.26
PCB#157(6CL)	1.21	5.11	2.24	0.880
PCB#167(6CL)	5.31	21.5	8.82	3.49
PCB#169(6CL)	ND	0.120	N.D.	N.D.
PCB#189(7CL)	1.14	6.38	2.05	1.15
Σ12PCB	66.8	276	109	39.8

Table S17. The data on congeners of PBBs in SPM in seawater (unit: $pg \cdot g^{-1}$)

Concentration (unit: $pg \cdot g^{-1}$)	AP1	AP2	KH1	KH2
PBB#15(2Br)	1.48	N.D.	N.D.	N.D.
PBB#52(4Br)	N.D.	0.273	N.D.	N.D.
PBB#153(6Br)	N.D.	2.34	N.D.	N.D.
PBB#180(7Br)	N.D.	N.D.	N.D.	N.D.
PBB#194(8Br)	N.D.	N.D.	N.D.	N.D.
Σ5PBB	1.48	2.61	N.D.	N.D.

Table S18. The data on congeners of dissolved PCDD/Fs in seawater

Concentration (unit: pg·L^{-1})	AP1	AP2	KH1	KH2
2,3,7,8-TeCDF	0.00322	N.D.	0.00302	N.D.
1,2,3,7,8-PeCDF	0.00223	0.00221	0.00302	0.00377
2,3,4,7,8-PeCDF	0.00210	N.D.	0.00320	0.00329
1,2,3,4,7,8-HxCDF	N.D.	0.00216	0.00144	0.00249
1,2,3,6,7,8-HxCDF	N.D.	0.00166	0.00143	0.00312
2,3,4,6,7,8-HxCDF	N.D.	0.00186	0.00112	N.D.
1,2,3,7,8,9-HxCDF	N.D.	N.D.	N.D.	N.D.
1,2,3,4,6,7,8-HpCDF	0.00752	0.00524	0.00441	0.00590
1,2,3,4,7,8,9-HpCDF	0.00112	0.00147	0.00124	N.D.
OCDF	0.0583	0.00950	0.00670	0.00809
2,3,7,8-TeCDD	N.D.	N.D.	N.D.	N.D.
1,2,3,7,8-PeCDD	0.00121	N.D.	0.00137	N.D.
1,2,3,4,7,8-HxCDD	N.D.	N.D.	N.D.	N.D.
1,2,3,6,7,8-HxCDD	N.D.	N.D.	0.000795	N.D.
1,2,3,7,8,9-HxCDD	N.D.	N.D.	N.D.	N.D.
1,2,3,4,6,7,8-HpCDD	0.0123	0.00574	0.00438	0.00367
OCDD	0.219	0.0659	0.0467	0.0271
Σ17PCDD/Fs	0.307	0.0957	0.0788	0.0574

Table S19. The data on congeners of dissolved PBDD/Fs in seawater

Concentration (unit: pg·L^{-1})	AP1	AP2	KH1	KH2
2,3,7,8-TeBDF	N.D.	N.D.	N.D.	N.D.
1,2,3,7,8-PeBDF	N.D.	N.D.	N.D.	N.D.
2,3,4,7,8-PeBDF	N.D.	N.D.	N.D.	N.D.
1,2,3,4,7,8-HxBDF	N.D.	N.D.	N.D.	N.D.
1,2,3,4,6,7,8-HpBDF	N.D.	N.D.	0.0319	0.0167
OctBDF	N.D.	N.D.	N.D.	N.D.
2,3,7,8,-TeBDD	N.D.	N.D.	N.D.	N.D.
1,2,3,7,8-PeBDD	N.D.	N.D.	N.D.	N.D.
1,2,3,4/6,7,8-HxBDD	N.D.	N.D.	N.D.	N.D.
1,2,3,7,8,9-HxBDD	N.D.	N.D.	N.D.	N.D.
1,2,3,4,6,7,8-HpBDD	N.D.	N.D.	N.D.	N.D.
OctBDD	N.D.	N.D.	N.D.	N.D.
Σ12PBDD/Fs	N.D.	N.D.	0.0319	0.0167

Table S20. The data on congeners of dissolved PBDEs in seawater

Concentration (unit: pg·L^{-1})	AP1	AP2	KH1	KH2
BDE #28(3Br)	0.232	0.217	0.233	0.228
BDE #47(4Br)	9.26	4.82	4.09	5.33
BDE #100(5Br)	1.44	0.589	0.645	0.614
BDE #99(5Br)	6.66	2.87	3.12	3.14
BDE #154(6Br)	0.362	0.194	0.225	0.210
BDE #153(6Br)	0.438	0.222	0.315	0.306
BDE #183(7Br)	0.0628	0.0628	0.0628	0.199
BDE #197(8Br)	0.0351	0.0351	0.821	0.388
BDE #203(8Br)	0.0659	0.0266	1.75	0.631
BDE #196(8Br)	0.134	0.120	1.58	0.637
BDE #208(9Br)	0.194	0.0699	8.86	3.24
BDE #207(9Br)	0.516	0.320	24.8	7.74
BDE #206(9Br)	0.548	0.395	22.8	6.66
BDE #209(10Br)	3.93	2.97	219	65.7
Σ14BDE	23.9	12.9	288	95.0

Table S21. The data on congeners of dissolved dioxin-like PCB in seawater

Concentration (unit: pg·L^{-1})	AP1	AP2	KH1	KH2
PCB#77(4CL)	0.153	0.113	0.249	0.242
PCB#81(4CL)	0.00894	0.00648	0.0109	0.00823
PCB#105(5CL)	0.431	0.311	0.408	0.413
PCB#114(5CL)	0.0324	0.0220	0.0316	0.0308
PCB#118(5CL)	1.30	1.04	1.20	1.22
PCB#123(5CL)	0.0168	0.0347	0.0358	0.0339
PCB#126(5CL)	0.00648	0.00358	0.00818	0.0102
PCB#156(6CL)	0.0995	0.108	0.104	0.103
PCB#157(6CL)	0.0161	0.0143	0.0148	0.0160
PCB#167(6CL)	0.0836	0.0767	0.0528	0.0537
PCB#169(6CL)	N.D.	N.D.	N.D.	N.D.
PCB#189(7CL)	0.00896	0.0139	0.0106	0.0121
Σ12PCB	2.16	1.74	2.12	2.14

Table S22. The data on congeners of dissolved PBBs in seawater

Concentration (unit: pg·L^{-1})	AP1	AP2	KH1	KH2
PBB#15(2Br)	0.0537	0.0284	0.0781	0.0195
PBB#52(4Br)	0.00309	0.00254	ND	0.00226
PBB#153(6Br)	N.D.	N.D.	N.D.	N.D.
PBB#180(7Br)	N.D.	N.D.	N.D.	N.D.
PBB#194(8Br)	N.D.	N.D.	N.D.	N.D.
Σ5PBB	0.0568	0.0310	0.0781	0.0217

Figure S2 shows the congener profiles of POPs in SPM and dissolved POPs in seawater. For Σ_{17} PCDD/Fs shown in Fig. S2(a) and S2(b), OCDD was the most abundant congener in the four sampling sites, both in the SPM (58.1%, 57.1%, 52.0%, and 58.5% for AP1, AP2, KH1, and KH2, respectively) and the dissolved PCDD/Fs (71.3%, 68.8%, 59.2%, and 47.2%, respectively). The second most abundant congener of PCDD/Fs was OCDF, which was consistent at the four sites, either in the SPM (14.5%, 12.0%, 23.1%, and 13.3% for AP1, AP2, KH1, and KH2, respectively) or the dissolved PCDD/Fs in seawater (19.0%, 9.90%, 8.50%, and 14.1%, respectively). Moreover, the proportion of the most toxic 1,2,3,7,8-PeCDD in SPM and dissolved PCDD/Fs in seawater was extremely low, whereas that of the most toxic 2,3,7,8-TeCDD was below the limit of quantification. Pouch et al. (2021) also found that OCDD and OCDF were dominant in seawater from the Hornsund fjord,[25] which is consistent with our findings.

All the 14 PBDE congeners were identified in this study. BDE#209(10Br) was the most abundant species in the SPM, accounting for 76.3% at AP1, 66.5% at AP2, 59.7% at KH1 and 66.7% at KH2 (Fig. S2(c)). However, the trend of BDE#209(10Br) in the dissolved PBDEs in seawater was not consistent with that in the SPM. As shown in Fig. S2(d), BDE#209(10Br) in the dissolved PBDEs in seawater was dominant only in KH1 and KH2, accounting for 76.0% and 69.1 %, respectively. Low-brominated BDE#47(4Br) in the dissolved PBDEs in seawater was the most abundant congener in AP1 and AP2, accounting for 38.8% and 37.4% of the total, respectively. Liu et al. (2015) indicated that BDE#47(4Br) tends to bioaccumulate more than higher brominated congeners and causes disruption of the endocrine system in aquatic organisms.[26] However, Ge et al. (2018) found that BDE#47(4Br) in the dissolved PBDEs in seawater from Sanggou Bay was 1.2–1.9%.[27] A regional difference in the PBDE congener profile in the dissolved PBDEs in seawater was observed in this study and that of Ge et al. (2018).[27]

Among the 12 congeners, PCB#118(5CL) was the most abundant congener in this

study. In the SPM in seawater, it accounted for 49.2–52.8% in all the four sites, as shown in Fig. S2(e). The second most common PCB congener in the SPM was PCB#105(5CL), which accounted for 13.2–17.6%. In the dissolved PCBs in seawater, PCB#118(5CL) accounted for 56.3–60.2% in all four sites, as shown in Fig. S2(f). PCB#105(5CL) was the second most common congener in the dissolution phase. Nguyen et al. (2017) reported that PCB#118(5CL) showed the highest concentration, followed by PCB#105(5CL), in Taiwan's terrestrial waters,[1] which is generally consistent with our seawater results. Additionally, PCB#156(6CL) and #167(6CL) in this study were ranked as the third and fourth most common congeners in the SPM, respectively, but accounted for less than 5% in the dissolved PCBs in seawater.

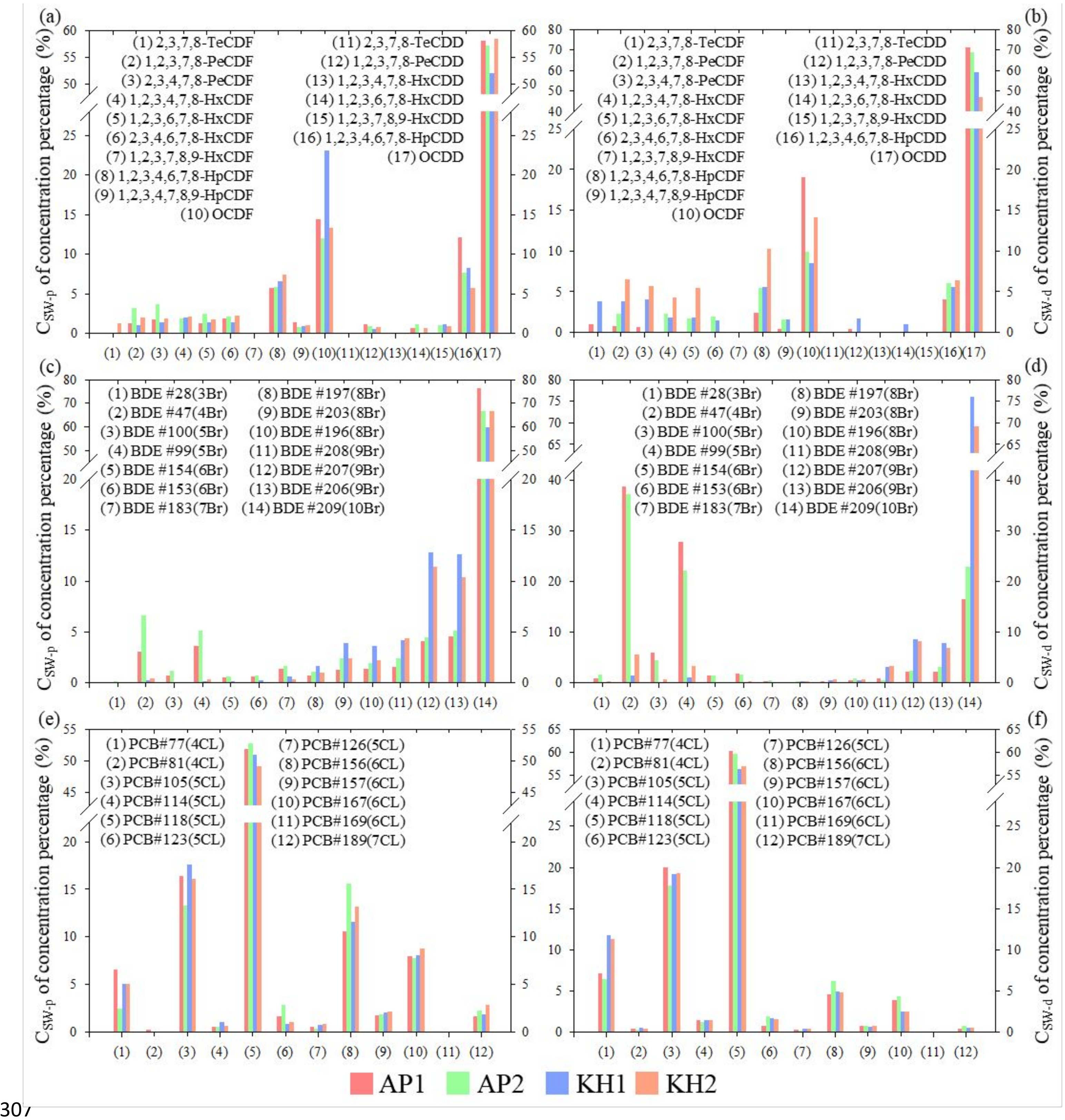

Fig. S2. Concentration percentages of individual congeners of POPs in SPM ($C_{SW\text{-}P}$, in the left column) and dissolved POPs ($C_{SW\text{-}D}$, in the right column) in seawater. (a, b) Σ_{17} PCDD/Fs, (c, d) Σ_{14} PBDEs, and (e, f) Σ_{12} dioxin-like PCBs.

S3.2. POPs in microplastics

A total of five types of POPs, including PCDD/Fs, PBDD/Fs, PBDEs, dioxin-like PCBs, and PBBs, were analyzed. Tables S23–S27 displayed the data on congeners of POPs in the surficial adsorption within MPs, while Tables S28–S32 showed the data on congeners of POPs in the total sorption within MPs. Furthermore, the characteristics of PCDD/F, PBDD/F, PBDE, dioxin-like PCB, and PBB congeners within MPs were discussed in the following paragraphs.

Table S23. The data on congeners of PCDD/Fs in the surficial adsorption within MPs

Concentration (unit: $pg{\cdot}g^{-1}$)	AP1	AP2	KH1	KH2
2,3,7,8-TeCDF	N.D.	N.D.	0.793	N.D.
1,2,3,7,8-PeCDF	4.00	1.36	0.483	N.D.
2,3,4,7,8-PeCDF	N.D.	1.00	N.D.	N.D.
1,2,3,4,7,8-HxCDF	N.D.	0.760	N.D.	N.D.
1,2,3,6,7,8-HxCDF	N.D.	0.988	N.D.	N.D.
2,3,4,6,7,8-HxCDF	N.D.	0.917	N.D.	N.D.
1,2,3,7,8,9-HxCDF	N.D.	N.D.	N.D.	N.D.
1,2,3,4,6,7,8-HpCDF	4.42	3.87	N.D.	N.D.
1,2,3,4,7,8,9-HpCDF	N.D.	N.D.	N.D.	N.D.
OCDF	5.58	2.41	0.946	N.D.
2,3,7,8-TeCDD	N.D.	N.D.	N.D.	N.D.
1,2,3,7,8-PeCDD	5.39	1.56	0.637	N.D.
1,2,3,4,7,8-HxCDD	N.D.	N.D.	N.D.	N.D.
1,2,3,6,7,8-HxCDD	N.D.	N.D.	N.D.	N.D.
1,2,3,7,8,9-HxCDD	N.D.	N.D.	N.D.	N.D.
1,2,3,4,6,7,8-HpCDD	5.06	3.98	0.610	N.D.
OCDD	18.1	15.3	2.03	N.D.
Σ17PCDD/Fs	42.6	32.2	5.50	N.D.

Table S24. The data on congeners of PBDD/Fs in the surficial adsorption within MPs

Concentration (unit: pg·g^{-1})	AP1	AP2	KH1	KH2
2,3,7,8-TeBDF	N.D.	N.D.	N.D.	N.D.
1,2,3,7,8-PeBDF	N.D.	N.D.	N.D.	N.D.
2,3,4,7,8-PeBDF	N.D.	N.D.	N.D.	N.D.
1,2,3,4,7,8-HxBDF	N.D.	15.9	12.0	N.D.
1,2,3,4,6,7,8-HpBDF	N.D.	69.6	110	N.D.
OctBDF	N.D.	N.D.	200	N.D.
2,3,7,8,-TeBDD	N.D.	N.D.	N.D.	N.D.
1,2,3,7,8-PeBDD	N.D.	N.D.	N.D.	N.D.
1,2,3,4/6,7,8-HxBDD	N.D.	N.D.	N.D.	N.D.
1,2,3,7,8,9-HxBDD	N.D.	N.D.	N.D.	N.D.
1,2,3,4,6,7,8-HpBDD	N.D.	N.D.	N.D.	N.D.
OctBDD	N.D.	N.D.	N.D.	N.D.
Σ12PBDD/Fs	N.D.	85.6	322	N.D.

Table S25. The data on congeners of PBDEs in the surficial adsorption within MPs

Concentration (unit: pg·g^{-1})	AP1	AP2	KH1	KH2
BDE #28(3Br)	26.3	31.7	21.5	N.D.
BDE #47(4Br)	269	569	143	N.D.
BDE #100(5Br)	86.7	116	22.6	N.D.
BDE #99(5Br)	202	334	57.9	N.D.
BDE #154(6Br)	838	185	33.4	N.D.
BDE #153(6Br)	602	213	26.6	N.D.
BDE #183(7Br)	1,660	529	137	N.D.
BDE #197(8Br)	378	326	279	N.D.
BDE #203(8Br)	446	579	798	N.D.
BDE #196(8Br)	453	754	544	N.D.
BDE #208(9Br)	1,680	467	3,380	N.D.
BDE #207(9Br)	1,960	722	4,690	N.D.
BDE #206(9Br)	2,780	1,430	5,230	N.D.
BDE #209(10Br)	15,800	4,720	39,600	N.D.
Σ14BDE	27,100	11,000	55,000	N.D.

Table S26. The data on congeners of dioxin-like PCBs in the surficial adsorption within MPs

Concentration (unit: pg·g $^{-1}$)	AP1	AP2	KH1	KH2
PCB#77(4CL)	1,010	106	30.5	N.D.
PCB#81(4CL)	24.4	4.21	1.36	N.D.
PCB#105(5CL)	441	209	72.7	N.D.
PCB#114(5CL)	N.D.	10.1	6.11	N.D.
PCB#118(5CL)	1,480	583	188	N.D.
PCB#123(5CL)	59.5	14.1	4.32	N.D.
PCB#126(5CL)	161	34.0	2.86	N.D.
PCB#156(6CL)	252	124	20.4	N.D.
PCB#157(6CL)	93.9	22.8	4.55	N.D.
PCB#167(6CL)	213	80.2	8.27	N.D.
PCB#169(6CL)	6.54	3.88	0.522	N.D.
PCB#189(7CL)	29.8	21.0	1.60	N.D.
Σ12PCB	3,770	1,210	341	N.D.

Table S27. The data on congeners of PBBs in the surficial adsorption within MPs

Concentration (unit: pg·g $^{-1}$)	AP1	AP2	KH1	KH2
PBB#15(2Br)	N.D.	N.D.	N.D.	N.D.
PBB#52(4Br)	212	N.D.	N.D.	N.D.
PBB#153(6Br)	3,010	N.D.	N.D.	N.D.
PBB#180(7Br)	92.9	N.D.	N.D.	N.D.
PBB#194(8Br)	N.D.	N.D.	N.D.	N.D.
Σ5PBB	3,310	N.D.	N.D.	N.D.

Table S28. The data on congeners of PCDD/Fs in the total sorption within MPs

Concentration (unit: $pg·g^{-1}$)	AP1	AP2	KH1	KH2
2,3,7,8-TeCDF	12.0	1.71	7.50	21.3
1,2,3,7,8-PeCDF	4.00	2.69	3.73	N.D.
2,3,4,7,8-PeCDF	N.D.	1.96	4.36	N.D.
1,2,3,4,7,8-HxCDF	N.D.	1.74	3.04	N.D.
1,2,3,6,7,8-HxCDF	N.D.	3.79	2.11	N.D.
2,3,4,6,7,8-HxCDF	N.D.	2.87	2.52	N.D.
1,2,3,7,8,9-HxCDF	N.D.	N.D.	N.D.	N.D.
1,2,3,4,6,7,8-HpCDF	8.76	23.2	5.66	14.5
1,2,3,4,7,8,9-HpCDF	N.D.	N.D.	1.21	N.D.
OCDF	14.5	7.35	4.77	37.9
2,3,7,8-TeCDD	N.D.	N.D.	N.D.	N.D.
1,2,3,7,8-PeCDD	5.39	2.33	2.75	28.7
1,2,3,4,7,8-HxCDD	N.D.	N.D.	N.D.	N.D.
1,2,3,6,7,8-HxCDD	N.D.	0.827	N.D.	N.D.
1,2,3,7,8,9-HxCDD	N.D.	ND	N.D.	N.D.
1,2,3,4,6,7,8-HpCDD	11.1	9.55	4.93	N.D.
OCDD	32.3	35.8	21.8	44.2
Σ17PCDD/Fs	88.1	93.8	64.4	147

Table S29. The data on congeners of PBDD/Fs in the total sorption within MPs

Concentration (unit: $pg·g^{-1}$)	AP1	AP2	KH1	KH2
2,3,7,8-TeBDF	N.D.	N.D.	N.D.	N.D.
1,2,3,7,8-PeBDF	N.D.	N.D.	N.D.	N.D.
2,3,4,7,8-PeBDF	N.D.	N.D.	N.D.	N.D.
1,2,3,4,7,8-HxBDF	N.D.	86.6	30.9	N.D.
1,2,3,4,6,7,8-HpBDF	217	563	213	N.D.
OctBDF	N.D.	499	200	N.D.
2,3,7,8,-TeBDD	N.D.	N.D.	N.D.	N.D.
1,2,3,7,8-PeBDD	N.D.	N.D.	N.D.	N.D.
1,2,3,4/6,7,8-HxBDD	N.D.	N.D.	N.D.	N.D.
1,2,3,7,8,9-HxBDD	N.D.	N.D.	N.D.	N.D.
1,2,3,4,6,7,8-HpBDD	N.D.	N.D.	N.D.	N.D.
OctBDD	N.D.	N.D.	N.D.	N.D.
Σ12PBDD/Fs	217	1150	444	N.D.

Table S30. The data on congeners of PBDEs in the total sorption within MPs

Concentration (unit: pg·g $^{-1}$)	AP1	AP2	KH1	KH2
BDE #28(3Br)	126	169	169	82.2
BDE #47(4Br)	1,120	1,630	1,680	365
BDE #100(5Br)	193	365	511	143
BDE #99(5Br)	533	1,550	1,230	446
BDE #154(6Br)	1,160	737	2,100	38.5
BDE #153(6Br)	987	815	795	97.2
BDE #183(7Br)	2,590	2,500	1,830	481
BDE #197(8Br)	712	1,800	445	1,620
BDE #203(8Br)	1,340	2,570	915	1,460
BDE #196(8Br)	1,310	3,710	665	1,440
BDE #208(9Br)	4,660	4,170	3,970	5,230
BDE #207(9Br)	5,430	7,900	5,380	6,090
BDE #206(9Br)	4,610	7,540	5,930	7,870
BDE #209(10Br)	44,800	35,200	47,000	77,000
Σ14BDE	69,500	70,700	72,600	102,000

Table S31. The data on congeners of dioxin-like PCBs in the total sorption within MPs

Concentration (unit: pg·g $^{-1}$)	AP1	AP2	KH1	KH2
PCB#77(4CL)	2,320	468	115	291
PCB#81(4CL)	72.6	14.5	5.64	N.D.
PCB#105(5CL)	1,320	916	278	356
PCB#114(5CL)	47.6	46.7	24.1	21.3
PCB#118(5CL)	3,840	2,500	747	780
PCB#123(5CL)	133	50.9	20.9	20.9
PCB#126(5CL)	348	233	9.95	N.D.
PCB#156(6CL)	475	500	90.9	86.5
PCB#157(6CL)	164	119	19.1	21.5
PCB#167(6CL)	419	452	47.5	62.4
PCB#169(6CL)	16.4	28.7	2.15	N.D.
PCB#189(7CL)	52.4	112	8.52	24.9
Σ12PCB	9,200	5,440	1,370	1,660

Table S32. The data on congeners of PBBs in the total sorption within MPs

Concentration (unit: pg·g^{-1})	AP1	AP2	KH1	KH2
PBB#15(2Br)	N.D.	N.D.	N.D.	N.D.
PBB#52(4Br)	244	9.63	N.D.	N.D.
PBB#153(6Br)	3,010	45.7	N.D.	N.D.
PBB#180(7Br)	92.9	7.82	N.D.	N.D.
PBB#194(8Br)	N.D.	N.D.	N.D.	N.D.
Σ5PBB	3,340	63.2	N.D.	N.D.

Figure S3 shows the congener profiles of POPs in the total sorption within floating MPs. As shown in Fig. S3(a), the most abundant congener in the total sorption within the MPs was OCDD, accounting for 36.7%, 38.2%, 33.9%, and 30.2% in the four samples, respectively. Other studies have also shown that OCDD is the most abundant among PCDD/F congeners in marine sediments, river water, soil, and lichens.[22,28,29] Therefore, highly chlorinated PCDD/Fs are abundant in environmental matrices. However, in the present study, the distributions of 2,3,7,8-TeCDF and 1,2,3,7,8-PeCDD in MPs differed from those in seawater. The mean of highly toxic 2,3,7,8-TeCDF (WHO2005-TEF of 0.1)[30] in the total sorption within the MPs was 10.4% at all four sampling sites (Fig. S3(a)), while 2,3,7,8-TeCDF in seawater did not exceed 4% (Fig. S2(a) and 2(b)). Moreover, the most toxic 1,2,3,7,8-PeCDD (WHO2005-TEF of 1)[30] among the MPs ranged from 2.5% to 19.6% (Fig. S3(a)), whereas 1,2,3,7,8-PeCDD in seawater was less than 2% (Fig. S2(a) and S2(b)). In general, the sorption of organic pollutants is mainly determined by their partitioning properties, such as the n-octanol/water partition coefficient and polymer-water partition coefficient in seawater.[31] Moreover, in marine organisms, 1,2,3,7,8-PeCDD is usually one of the dominant congeners contributing the most to toxic equivalents (TEQs).[32] In contrast to the diverse congeners of PCDD/Fs within MPs, the detected PBDD/F congeners within MPs in this study were limited to three furan-type congeners. Regarding the total sorption of PBDD/Fs within the MPs, Fig. S3(b) shows that 1,2,3,4,6,7,8-HpBDF was the dominant congener, accounting for 100%, 49.0%, and 47.9% of AP1, AP2, and KH1, respectively. Other studies have also found that 1,2,3,4,6,7,8-HpBDF is the dominant congener of PBDD/Fs in European eels, cod liver-derived products, and marine sediments.[33,34,35]

All 14 PBDE congeners were detected within the MPs. BDE#209(10Br) was the most abundant PBDE congener in the total sorption of MPs. BDE#209(10Br) was 64.4%, 49.8%, 64.7%, and 75.2% for AP1, AP2, KH1, and KH2, respectively (Fig. S3(c)). Additionally, BDE#47(4Br), a less-brominated congener, was a relatively insignificant congener. However, in the dissolved PBDEs in seawater, BDE#47(4Br)

contributed approximately 40% in some cases (Fig. S2(d)). Xu et al. (2019) found that MPs have a stronger sorption capacity for high-brominated congeners of PBDEs compared to low-brominated congeners.[36] High-brominated PBDEs were more hydrophobic than low-brominated PBDEs.[37] Therefore, low-brominated congeners of PBDEs may not be easily adsorbed by MPs in seawater.

For total sorption within the MPs, Fig. S3(d) shows that the most abundant congener was PCB#118(5CL), accounting for 41.7%, 45.9%, 54.6%, and 46.9% of AP1, AP2, KH1, and KH2, respectively. PCB#105(5CL) and PCB#77(4CL) were the second most abundant. Wang et al. (2021) also found that PCB#118(5CL) was the most dominant congener on MP pellets from beaches.[38] Moreover, in a study area close to this study, Jiang et al. (2011) found that tetra-CBs in surface sediments were dominant off the coast of Tainan near AP1 and AP2, and tetra- and penta-CBs were dominant along the Kaohsiung coast near KH1 and KH2.[39] Cheng et al. (2021) also indicated that tri-, tetra-, and penta-CBs were dominant congeners found in surface sediments from the southwestern coast of Taiwan.[40] Therefore, the dominant dioxin-like PCB congeners found in marine floating MPs in this study were tetra- and penta-CBs. This finding is consistent with those of previous studies on coastal sediments and microplastic pellets on beaches.

As shown in Fig. S3(e), PBB #153 (6Br) was dominant in the total sorption within the MPs, accounting for 89.9% and 72.4% of AP1 and AP2, respectively. In marine mammals from North America, PBB#153(6Br) was also dominant among hexa-BBs.[41] In marine fish of the North Sea, Gieroń et al. (2010) found that the dominant congeners of PBBs were tetra-BBs (PBB#49 and PBB#52) and hexa-BBs (PBB#153).[42] In this study, the distribution trend of PBBs congeners within MPs was roughly consistent with that in marine organisms.

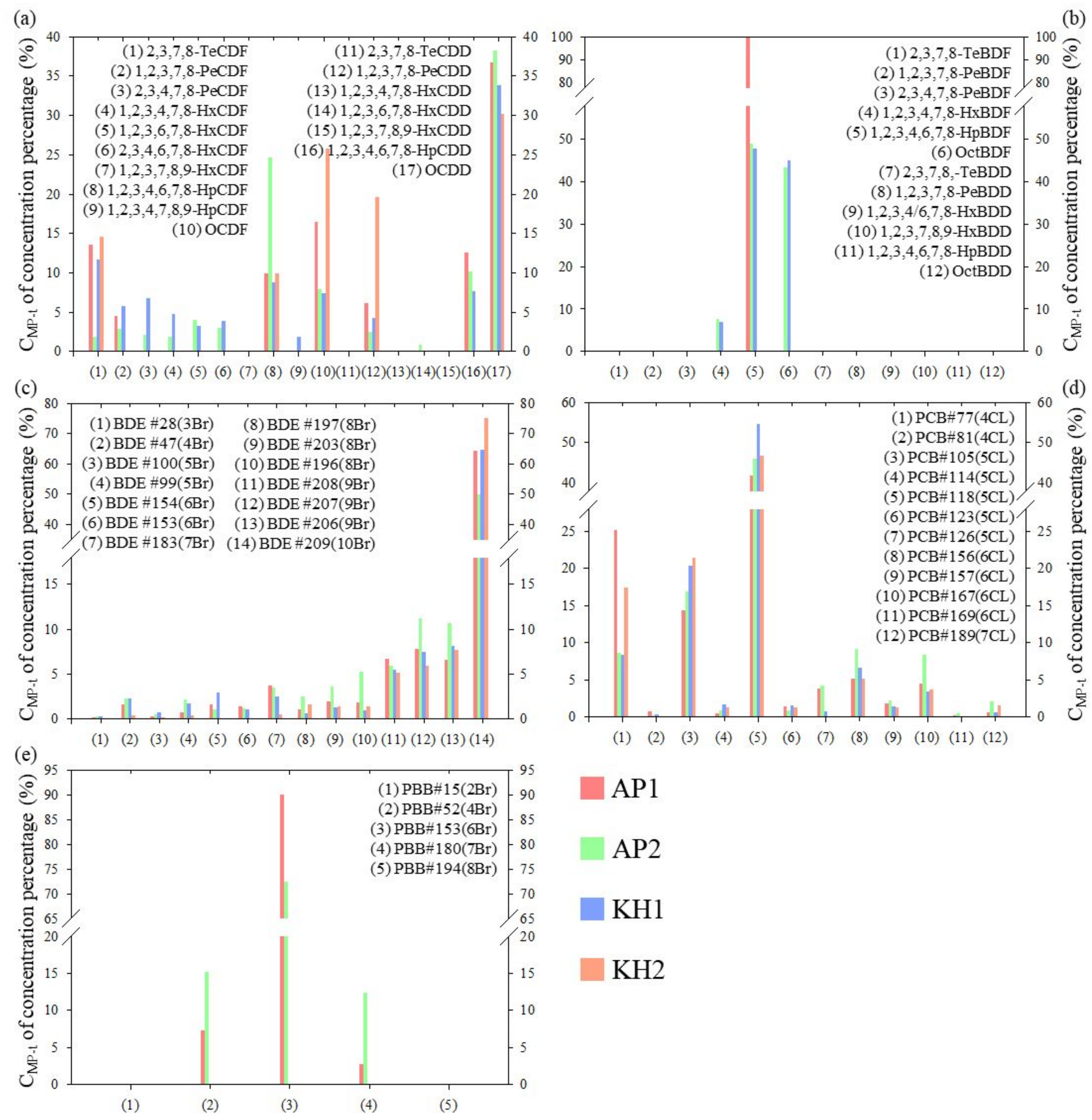

Fig. S3. Concentration percentages of individual congeners in the total sorption within floating MPs (C_{MP-T}). (a) Σ_{17} PCDD/Fs, (b) Σ_{12} PBDD/Fs, (c) Σ_{14} PBDEs, (d) Σ_{12} dioxin-like PCBs, and (e) Σ_5 PBBs.

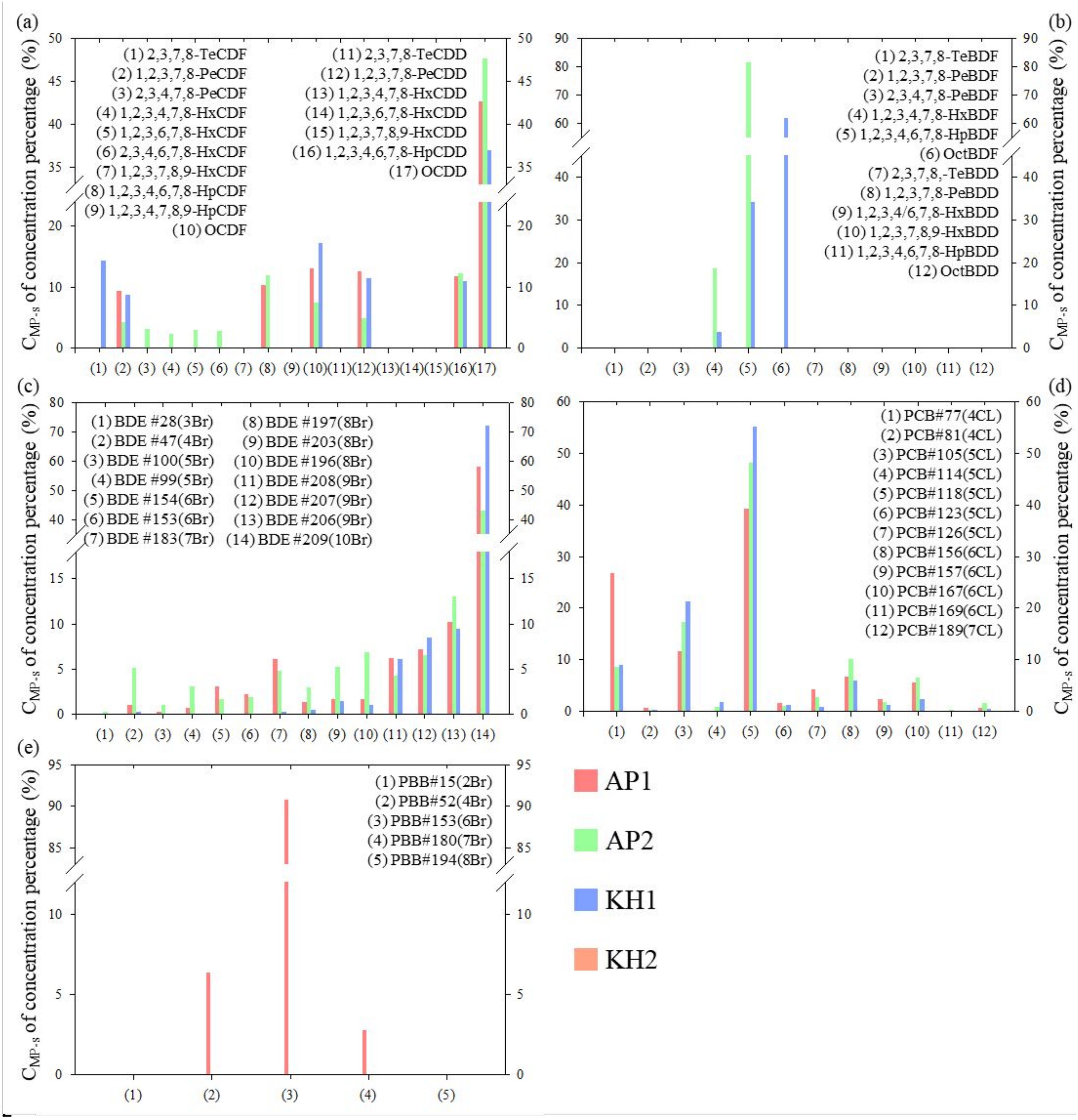

Fig. S4. Concentration percentages of individual congeners in the surficial adsorption within floating MPs ($C_{MP\text{-}S}$). (a) Σ_{17} PCDD/Fs, (b) Σ_{12} PBDD/Fs, (c) Σ_{14} PBDEs, (d) Σ_{12} dioxin-like PCBs, and (e) Σ_5 PBBs.

S4. The n-octanol-water partition coefficient (K_{OW})

In this study, the logarithm of K_{OW} values of POP homologues represents the average of congeners with the same number of chlorine or bromine atoms, as shown in Table S33. Table S33 provides the average logarithm of K_{OW} for POP homologues, derived from the data presented in Tables S34–S38. Tables S34–S38 list the logarithm of K_{OW} values of individual POP congeners from literature.

Table S33. Average of logarithm of K_{OW} of POP homologues

(a) PCDD/Fs homologues	Log K_{OW}
TCDF	6.29
PeCDFs	6.72
HxCDFs	7.04
HpCDFs	7.50
OCDF	8.10
TCDD	6.64
PeCDD	6.82
HxCDDs	7.40
HpCDD	7.81
OCDD	8.19

(b) PBDD/Fs homologues	Log K_{OW}
TBDF	6.35
PeBDFs	7.04
HxBDF	7.54
HpBDF	7.93
OBDF	8.26
TBDD	6.72
PeBDD	7.32
HxBDDs	7.77
HpBDD	8.16
OBDD	8.60

(c) PBDEs homologues	Log K_{OW}
TriBDE	5.99
TetraBDE	6.66
PentaBDEs	7.23
HexaBDEs	7.77
HeptaBDE	8.32
OctaBDEs	8.93
NonaBDEs	9.46
DecaBDE	9.58

(d) PCBs homologues	Log K_{OW}
TetraCBs	6.48
PentaCBs	6.72
HexaCBs	7.31
HeptaCB	7.74

(e) PBBs homologues	Log K_{OW}
DiBB	5.72
TetraBB	6.50
HexaBB	7.75
HeptaBB	8.30
OctaBB	8.70

Table S34. K_{OW} of PCDD/F congeners

PCDD/F congeners	Log K_{OW}	literature
2,3,7,8-TeCDF	6.1	U.S. EPA (2004)[43]
	6.5	Fernandez-Gonzalez et al. (2015)[44]
	6.53	Geyer et al. (2000)[45]
	6.1	Chen et al. (2001)[46]
	6.225	Chen et al. (2001)[46]
1,2,3,7,8-PeCDF	6.79	U.S. EPA (2004)[43]
	6.8	Fernandez-Gonzalez et al. (2015)[44]
2,3,4,7,8-PeCDF	6.5	U.S. EPA (2004)[43]
	6.8	Fernandez-Gonzalez et al. (2015)[44]
	6.92	Geyer et al. (2000)[45]
	6.5	Chen et al. (2001)[46]
	6.757	Chen et al. (2001)[46]
1,2,3,4,7,8-HxCDF	7	U.S. EPA (2004)[43]
	6.9	Fernandez-Gonzalez et al. (2015)[44]
	7	Chen et al. (2001)[46]
	7.184	Chen et al. (2001)[46]
1,2,3,6,7,8-HxCDF	7	Fernandez-Gonzalez et al. (2015)[44]
	7.196	Chen et al. (2001)[46]
2,3,4,6,7,8-HxCDF	7.1	Fernandez-Gonzalez et al. (2015)[44]
	6.937	Chen et al. (2001)[46]
1,2,3,7,8,9-HxCDF	7	Fernandez-Gonzalez et al. (2015)[44]
	7.125	Chen et al. (2001)[46]
1,2,3,4,6,7,8-HpCDF	7.4	U.S. EPA (2004)[43]
	7.4	Fernandez-Gonzalez et al. (2015)[44]
	7.92	Geyer et al. (2000)[45]
	7.4	Chen et al. (2001)[46]
	7.477	Chen et al. (2001)[46]
1,2,3,4,7,8,9-HpCDF	7.3	Fernandez-Gonzalez et al. (2015)[44]
	7.616	Chen et al. (2001)[46]
OCDF	8	U.S. EPA (2004)[43]
	7.6	Fernandez-Gonzalez et al. (2015)[44]
	8.78	Geyer et al. (2000)[45]
	8	Chen et al. (2001)[46]
2,3,7,8-TeCDD	6.8	U.S. EPA (2004)[43]

PCDD/F congeners	Log K_{OW}	literature
	6.3	Fernandez-Gonzalez et al. (2015)[44]
	6.64	Geyer et al. (2000)[45]
	6.8	Chen et al. (2001)[46]
	6.656	Chen et al. (2001)[46]
1,2,3,7,8-PeCDD	6.64	U.S. EPA (2004)[43]
	6.6	Fernandez-Gonzalez et al. (2015)[44]
	7.215	Chen et al. (2001)[46]
1,2,3,4,7,8-HxCDD	7.8	U.S. EPA (2004)[43]
	6.9	Fernandez-Gonzalez et al. (2015)[44]
	7.8	Chen et al. (2001)[46]
	7.628	Chen et al. (2001)[46]
1,2,3,6,7,8-HxCDD	6.9	Fernandez-Gonzalez et al. (2015)[44]
	7.639	Chen et al. (2001)[46]
1,2,3,7,8,9-HxCDD	6.9	Fernandez-Gonzalez et al. (2015)[44]
	7.61	Chen et al. (2001)[46]
1,2,3,4,6,7,8-HpCDD	8	U.S. EPA (2004)[43]
	7.2	Fernandez-Gonzalez et al. (2015)[44]
	8	Chen et al. (2001)[46]
	8.051	Chen et al. (2001)[46]
OCDD	8.2	U.S. EPA (2004)[43]
	7.5	Fernandez-Gonzalez et al. (2015)[44]
	8.6	Geyer et al. (2000)[45]
	8.2	Chen et al. (2001)[46]
	8.45	Chen et al. (2001)[46]

Table S35. K_{OW} of PBDD/F congeners

PBDD/F congeners	Log K_{OW}	literature
2,3,7,8-TeBDF	5.98	Puzyn et al. (2008)[47]
	6.71	
1,2,3,7,8-PeBDF	7.04	
	6.98	
2,3,4,7,8-PeBDF	7.09	
1,2,3,4,7,8-HxBDF	7.54	
1,2,3,4,6,7,8-HpBDF	7.93	
OctBDF	8.26	
2,3,7,8-TeBDD	6.5	
	6.94	
1,2,3,7,8-PeBDD	7.32	
1,2,3,4,7,8-HxBDD	7.84	
1,2,3,6,7,8-HxBDD	7.86	
1,2,3,7,8,9-HxBDD	7.62	
1,2,3,4,6,7,8-HpBDD	8.16	
OctBDD	8.6	

Table S36. K_{OW} of PBDE congeners

PBDE congeners	Log K_{OW}	literature
BDE #28(3Br)	5.94	Yue and Li (2013)[48]
	5.96	Yue and Li (2013)[48]
	5.94	EFSA CONTAM (2011)[49]
	5.96	EFSA CONTAM (2011)[49]
	5.94	Braekevelt et al. (2003)[50]
	5.94	ATSDR (2004)[51]
	6.24	Bao et al. (2011)[52]
	5.98	Tittlemier et al. (2002)[53]
BDE #47(4Br)	6.81	Yue and Li (2013)[48]
	6.76	Yue and Li (2013)[48]
	6.66	Yue and Li (2013)[48]
	6.81	EFSA CONTAM (2011)[49]
	6.76	EFSA CONTAM (2011)[49]
	6.81	Braekevelt et al. (2003)[50]
	6.8	Bao et al. (2011)[52]
	6.01	Palm et al. (2002)[54]
	6.19	Palm et al. (2002)[54]
	6.77	Palm et al. (2002)[54]
	6.81	ATSDR (2004)[51]
	6.78	Kuramochi et al. (2007)[55]
BDE #100(5Br)	7.24	Yue and Li (2013)[48]
	7.49	Yue and Li (2013)[48]
	7.31	Yue and Li (2013)[48]
	7.24	EFSA CONTAM (2011)[49]
	7.49	EFSA CONTAM (2011)[49]
	7.24	Braekevelt et al. (2003)[50]
	7.09	Bao et al. (2011)[52]
	7.24	ATSDR (2004)[51]
	6.86	Tittlemier et al. (2002)[53]
BDE #99(5Br)	7.32	Yue and Li (2013)[48]
	7.27	Yue and Li (2013)[48]
	7.12	Yue and Li (2013)[48]
	7.32	EFSA CONTAM (2011)[49]
	7.27	EFSA CONTAM (2011)[49]

PBDE congeners	Log K_{OW}	literature
	7.32	Braekevelt et al. (2003)[50]
	7.38	Bao et al. (2011)[52]
	6.53	Palm et al. (2002)[54]
	6.71	Palm et al. (2002)[54]
	7.66	Palm et al. (2002)[54]
	7.32	ATSDR (2004)[51]
	7.39	Kuramochi et al. (2007)[55]
BDE #154(6Br)	7.82	Yue and Li (2013)[48]
	7.89	Yue and Li (2013)[48]
	7.7	Yue and Li (2013)[48]
	7.82	EFSA CONTAM (2011)[49]
	7.89	EFSA CONTAM (2011)[49]
	7.82	Braekevelt et al. (2003)[50]
	7.62	Bao et al. (2011)[52]
	7.82	ATSDR (2004)[51]
	7.39	Tittlemier et al. (2002)[53]
BDE #153(6Br)	7.9	Yue and Li (2013)[48]
	7.58	Yue and Li (2013)[48]
	7.48	Yue and Li (2013)[48]
	7.9	EFSA CONTAM (2011)[49]
	7.58	EFSA CONTAM (2011)[49]
	7.9	Braekevelt et al. (2003)[50]
	7.86	Bao et al. (2011)[52]
	7.9	ATSDR (2004)[51]
	8.05	Kuramochi et al. (2007)[55]
	7.62	Tittlemier et al. (2002)[53]
BDE #183(7Br)	8.27	Yue and Li (2013)[48]
	8.35	Yue and Li (2013)[48]
	8.12	Yue and Li (2013)[48]
	8.27	EFSA CONTAM (2011)[49]
	8.35	EFSA CONTAM (2011)[49]
	8.27	Braekevelt et al. (2003)[50]
	8.61	Bao et al. (2011)[52]
BDE #197(8Br)	8.54	Papa et al. (2009)[56]; Papa et al. (2011)[57]
	9.09	Braekevelt et al. (2003)[50]; Papa et al. (2011)[57]

PBDE congeners	Log K_{OW}	literature
BDE #203(8Br)	8.7	Papa et al. (2009)[56]; Papa et al. (2011)[57]
	9.09	Braekevelt et al. (2003)[50]; Papa et al. (2011)[58]
BDE #196(8Br)	8.7	Papa et al. (2009)[56]; Papa et al. (2011)[57]
	9.09	Braekevelt et al. (2003)[50]; Papa et al. (2011)[57]
	9.29	Bao et al. (2011)[52]
BDE #208(9Br)	9.65	Bao et al. (2011)[52]
BDE #207(9Br)	9.65	Bao et al. (2011)[52]
BDE #206(9Br)	9.08	Braekevelt et al. (2003)[50]
BDE #209(10Br)	9.97	Yue and Li (2013)[48]
	9.31	Yue and Li (2013)[48]
	9.16	Yue and Li (2013)[48]
	9.87	Bao et al. (2011)[52]

Table S37. K_{OW} of dioxin-like PCB congeners

PCB congeners	Log K_{OW}	literature
PCB#77(4CL)	6.5	U.S. EPA (2004)[43]
	6.5	Fernandez-Gonzalez et al. (2015)[44]
	6.63	Geyer et al. (2000)[45]
PCB#81(4CL)	6.36	U.S. EPA (2004)[43]
	6.5	Fernandez-Gonzalez et al. (2015)[44]
	6.4	Geyer et al. (2000)[45]
PCB#105(5CL)	5.81	Kelly et al. (2007)[58]
	6	U.S. EPA (2004)[43]
	6.7	Fernandez-Gonzalez et al. (2015)[44]
	6.65	Geyer et al. (2000)[45]
PCB#114(5CL)	6.65	U.S. EPA (2004)[43]
	6.7	Fernandez-Gonzalez et al. (2015)[44]
	6.65	Geyer et al. (2000)[45]
PCB#118(5CL)	6.74	Kelly et al. (2007)[58]
	7.12	U.S. EPA (2004)[43]
	6.8	Fernandez-Gonzalez et al. (2015)[44]
	7.12	Rapaport et al. (1984)[59]
PCB#123(5CL)	6.74	U.S. EPA (2004)[43]
PCB#126(5CL)	6.89	U.S. EPA (2004)[43]
	7	Fernandez-Gonzalez et al. (2015)[44]
	7.2	Geyer et al. (2000)[45]
PCB#156(6CL)	7.16	U.S. EPA (2004)[43]
	7.2	Fernandez-Gonzalez et al. (2015)[44]
	7.18	Geyer et al. (2000)[45]
PCB#157(6CL)	7.19	U.S. EPA (2004)[43]
	7.2	Fernandez-Gonzalez et al. (2015)[44]
PCB#167(6CL)	7.09	U.S. EPA (2004)[45]
	7.3	Fernandez-Gonzalez et al. (2015)[44]
	7.27	Geyer et al. (2000)[45]
PCB#169(6CL)	7.46	U.S. EPA (2004)[43]
	7.6	Fernandez-Gonzalez et al. (2015)[44]
	7.41	Geyer et al. (2000)[45]
	7.68	Geyer et al. (2000)[45]
PCB#189(7CL)	7.71	U.S. EPA (2004)[43]

PCB congeners	Log K_{OW}	literature
	7.8	Fernandez-Gonzalez et al. (2015)[44]
	7.71	Geyer et al. (2000)[45]

Table S38. K_{OW} of PBB congeners

PBB congeners	Log K_{OW}	literature
PBB#15(2Br)	5.72	Geyer et al. (2000)[44]
PBB#52(4Br)	6.5	Geyer et al. (2000)[44]
	6.5	EFSA CONTAM (2010)[60]
PBB#153(6Br)	7.5	Geyer et al. (2000)[45]
	8	EFSA CONTAM (2010)[60]
PBB#180(7Br)	8.3	EFSA CONTAM (2010)[60]
PBB#194(8Br)	8.7	EFSA CONTAM (2010)[60]

S5. The elemental analysis of suspended particulate matter (SPM)

At sampling sites AP1 and KH1, the elemental composition of SPM in seawater was analyzed using a portable X-ray fluorescence (pXRF) spectrometer. The primary elements in the SPM were chlorine (Cl), potassium (K), calcium (Ca), and light elements, including sodium (Na), carbon (C), hydrogen (H), and oxygen (O), as detailed in Table S39. Additionally, samples of SPM were transferred into a furnace and incinerated at 550 °C until a constant weight was achieved to differentiate between fixed solids and volatile solids. The determination of volatile solids provided an approximation of the organic matter present in the SPM, as shown in Table S40.

Table S39. Elemental component of SPM

AP1			KH1		
Elements	Concentration (mg/kg)	Proportion	Elements	Concentration (mg/kg)	Proportion
Si	20,500 ± 566	2.05%	Si	10,600 ± 251	1.06%
S	27,200 ± 903	2.72%	S	9,470 ± 337	0.948%
Cl	296,000 ± 4,770	29.6%	Cl	109,000 ± 1,280	10.9%
K	101,000 ± 1,690	10.1%	K	25,700 ± 325	2.57%
Ca	63,900 ± 1,100	6.38%	Ca	16,300 ± 214	1.63%
Ti	37,200 ± 1,480	3.72%	Ti	15,800 ± 522	1.58%
V	23,900 ± 931	2.39%	V	9,910 ± 320	0.992%
Mn	2,240 ± 208	0.224%	Mn	613 ± 59.3	0.0613%
Fe	20,600 ± 556	2.06%	Fe	1,700 ± 77.4	0.170%
Zn	49,100 ± 835	4.91%	Zn	13,000 ± 163	1.30%
Rb	98.0 ± 7.68	0.00979%	Rb	23.0 ± 2.09	0.00230%
Sr	486 ± 16.3	0.0486%	Sr	118 ± 3.88	0.0118%
Zr	174 ± 10.8	0.0174%	Zr	69.0 ± 3.74	0.00690%
Bi	37.0 ± 8.67	0.00370%	Mo	51.0 ± 5.03	0.00510%
LE*	282,000 ± 8,160	28.2%	Pb	23.0 ± 4.88	0.00230%
Al	76,400 ± 10,700	7.63%	LE*	787,000 ± 2,400	78.8%

*: LE represents the light elements including sodium (Na), carbon (C), hydrogen (H), and oxygen (O).

Table S40. The proportion of fixed solids and volatile solids in SPM

Sites	AP1	KH1
Fixed solids (%)	78.2	71.1
Volatile solids (%)	21.8	28.9

S6. The concentration of POPs in the virgin microplastic pellets

The concentrations of POPs in the virgin low-density polyethylene (LDPE) pellet samples obtained from two suppliers were shown in Table S41. The results for virgin pellet indicated that the concentrations of POPs were either below detection limit or present only in trace amounts.

Table S41. The concentrations of POPs in the virgin LDPE pellets

PCDD/Fs ($pg \cdot g^{-1}$)	White pellet	Black pellet
2,3,7,8-TeCDF	N.D.	N.D.
1,2,3,7,8-PeCDF	0.0334	N.D.
2,3,4,7,8-PeCDF	N.D.	N.D.
1,2,3,4,7,8-HxCDF	N.D.	N.D.
1,2,3,6,7,8-HxCDF	N.D.	N.D.
2,3,4,6,7,8-HxCDF	N.D.	N.D.
1,2,3,7,8,9-HxCDF	N.D.	N.D.
1,2,3,4,6,7,8-HpCDF	N.D.	N.D.
1,2,3,4,7,8,9-HpCDF	N.D.	N.D.
OCDF	0.0935	0.122
2,3,7,8-TeCDD	0.0434	N.D.
1,2,3,7,8-PeCDD	N.D.	N.D.
1,2,3,4,7,8-HxCDD	N.D.	N.D.
1,2,3,6,7,8-HxCDD	N.D.	N.D.
1,2,3,7,8,9-HxCDD	0.0819	0.121
1,2,3,4,6,7,8-HpCDD	N.D.	0.0842
OCDD	0.0940	0.213
Σ_{17}PCDD/Fs	0.346	0.540

PBDEs ($pg \cdot g^{-1}$)	White pellet	Black pellet
BDE #28(3Br)	0.949	0.944
BDE #47(4Br)	6.34	6.30
BDE #100(5Br)	1.12	1.12
BDE #99(5Br)	9.15	9.10
BDE #154(6Br)	1.05	4.03
BDE #153(6Br)	3.10	11.5
BDE #183(7Br)	6.22	13.9
BDE #197(8Br)	3.48	10.1
BDE #203(8Br)	2.63	16.8
BDE #196(8Br)	5.08	27.7
BDE #208(9Br)	6.92	13.9
BDE #207(9Br)	11.4	11.3
BDE #206(9Br)	7.75	22.7
BDE #209(10Br)	82.4	204
Σ_{14}PBDEs	148	354

PBDD/Fs ($pg \cdot g^{-1}$)	White	Black pellet
2,3,7,8-TeBDF	N.D.	N.D.
1,2,3,7,8-PeBDF	N.D.	N.D.
2,3,4,7,8-PeBDF	N.D.	N.D.
1,2,3,4,7,8-HxBDF	N.D.	N.D.
1,2,3,4,6,7,8-HpBDF	N.D.	3.01
OctBDF	N.D.	N.D.
2,3,7,8,-TeBDD	N.D.	N.D.
1,2,3,7,8-PeBDD	N.D.	N.D.
1,2,3,4/6,7,8-HxBDD	N.D.	N.D.
1,2,3,7,8,9-HxBDD	N.D.	N.D.
1,2,3,4,6,7,8-HpBDD	N.D.	N.D.
OctBDD	N.D.	N.D.
Σ_{12}PBDD/Fs	N.D.	3.01

dioxin-like PCBs ($pg \cdot g^{-1}$)	White	Black pellet
PCB#77(4CL)	2.71	2.66
PCB#81(4CL)	N.D.	N.D.
PCB#105(5CL)	N.D.	N.D.
PCB#114(5CL)	N.D.	N.D.
PCB#118(5CL)	N.D.	N.D.
PCB#123(5CL)	N.D.	N.D.
PCB#126(5CL)	N.D.	0.309
PCB#156(6CL)	N.D.	N.D.
PCB#157(6CL)	N.D.	N.D.
PCB#167(6CL)	N.D.	N.D.
PCB#169(6CL)	N.D.	N.D.
PCB#189(7CL)	N.D.	N.D.
Σ_{12}PCB	2.71	2.97

PBBs ($pg \cdot g^{-1}$)	White	Black pellet
PBB#15(2Br)	N.D.	N.D.
PBB#52(4Br)	N.D.	N.D.
PBB#153(6Br)	N.D.	N.D.
PBB#180(7Br)	N.D.	N.D.
PBB#194(8Br)	N.D.	N.D.
Σ_5PCB	N.D.	N.D.

S7. The data on EF_{SW-P}, EF_{MP-T}, and the concentration percentage (%) of both PCDD/F and PBDE homologues

Table S42. The data on logarithm of EF_{SW-P} of POPs and TEQ-POPs

	AP1	AP2	KH1	KH2	Mean	SD
PCDD/Fs	5.10	5.05	5.54	5.57	5.32	0.278
PBDD/Fs	-	-	6.80	6.43	6.62	0.262
PBDEs	5.46	5.34	5.21	5.19	5.30	0.126
PCBs	4.49	5.20	4.71	4.27	4.67	0.398
PBBs	4.41	4.93	-	-	4.67	0.368
TEQ-PCDD/Fs	5.55	5.64	5.17	5.49	5.46	0.205
TEQ-PBDD/Fs	-	-	6.87	6.36	6.62	0.361
TEQ-PCBs	4.73	5.4	4.98	4.49	4.90	0.389

Table S43. The data on logarithm of EF_{MP-T} of POPs and TEQ-POPs

	AP1	AP2	KH1	KH2	Mean	SD
PCDD/Fs	5.46	5.99	5.91	6.41	5.94	0.389
PBDD/Fs	-	-	7.14	-	7.14	-
PBDEs	6.46	6.74	5.40	6.03	6.16	0.583
PCBs	6.63	6.49	5.81	5.89	6.21	0.415
PBBs	7.77	6.31	-	-	7.04	1.03
TEQ-PCDD/Fs	6.44	6.75	6.24	7.24	6.67	0.436
TEQ-PBDD/Fs	-	-	7.22	-	7.22	-
TEQ-PCBs	7.69	7.76	6.09	4.81	6.59	1.41

Table S44. The data on the concentration percentage (%) of PCDD/F homologues in SPM in seawater

	AP1	AP2	KH1	KH2	Mean	SD
TCDF	N.D.	N.D.	N.D.	1.26	1.26	N.D.
PeCDFs	3.11	6.88	2.57	3.96	4.13	1.92
HxCDFs	3.19	6.55	4.77	6.27	5.20	1.55
HpCDFs	7.15	6.70	7.54	8.55	7.48	0.788
OCDF	14.5	12.0	23.1	13.3	15.7	5.06
TCDD	N.D.	N.D.	N.D.	N.D.	N.D.	N.D.
PeCDD	1.13	0.909	0.546	0.842	0.857	0.241
HxCDDs	0.744	2.20	1.15	1.61	1.43	0.627
HpCDD	12.1	7.69	8.28	5.75	8.47	2.68
OCDD	58.1	57.1	52.0	58.5	56.4	3.00

Table S45. The data on the concentration percentage (%) of PBDE homologues in SPM in seawater

	AP1	AP2	KH1	KH2	Mean	SD
TriBDE	0.0525	0.158	0.00763	0.00909	0.0567	0.0705
TetraBDE	3.05	6.64	0.293	0.422	2.60	2.97
PentaBDEs	4.36	6.27	0.204	0.381	2.80	3.00
HexaBDEs	1.22	1.31	0.358	0.147	0.760	0.594
HeptaBDE	1.38	1.68	0.647	0.393	1.02	0.603
OctaBDEs	3.37	5.43	9.13	5.61	5.88	2.39
NonaBDEs	10.3	12.0	29.7	26.2	19.5	9.83
DecaBDE	76.3	66.5	59.7	66.9	67.3	6.82

Table S46. The data on the concentration percentage (%) of PCDD/F homologues in the floating MPs

	AP1	AP2	KH1	KH2	Mean	SD
TCDF	13.6	1.82	11.6	14.5	10.4	5.84
PeCDFs	4.54	4.96	12.6	N.D.	7.35	4.52
HxCDFs	ND	8.95	11.9	N.D.	10.4	2.09
HpCDFs	9.95	24.7	10.7	9.91	13.8	7.26
OCDF	16.5	7.84	7.40	25.8	14.4	8.70
TCDD	N.D.	N.D.	N.D.	N.D.	N.D.	N.D.
PeCDD	6.12	2.48	4.27	19.6	8.11	7.79
HxCDDs	ND	0.882	N.D.	N.D.	0.882	N.D.
HpCDD	12.6	10.2	7.65	N.D.	10.1	2.46
OCDD	36.7	38.2	33.9	30.2	34.7	3.55

Table S47. The data on the total concentration percentage (%) of PBDE homologues in the floating MPs

	AP1	AP2	KH1	KH2	Mean	SD
TriBDE	0.181	0.239	0.233	0.0803	0.183	0.0736
TetraBDE	1.60	2.30	2.31	0.357	1.64	0.920
PentaBDEs	1.04	2.71	2.40	0.576	1.68	1.03
HexaBDEs	3.09	2.20	3.99	0.133	2.35	1.65
HeptaBDE	3.72	3.54	2.52	0.470	2.56	1.49
OctaBDEs	4.83	11.4	2.79	4.42	5.87	3.82
NonaBDEs	21.1	27.7	21.1	18.7	22.2	3.88
DecaBDE	64.4	49.8	64.7	75.2	63.5	10.4

Table S48. The data on logarithm of EF_{SW-P} of POP homologues in SPM in seawater

	AP1	AP2	KH1	KH2	Mean	SD
TCDF	-	-	-	-	-	-
PeCDFs	5.44	5.52	5.05	5.08	5.27	0.243
HxCDFs	-	5.09	5.51	5.38	5.33	0.214
HpCDFs	5.51	5.03	5.56	5.49	5.40	0.247
OCDF	4.98	5.13	5.97	5.55	5.41	0.446
TCDD	-	-	-	-	-	-
PeCDD	5.56	-	5.04	-	5.30	0.368
HxCDDs	-	-	5.59	-	5.59	-
HpCDD	5.58	5.16	5.71	5.53	5.49	0.238
OCDD	5.01	4.97	5.48	5.67	5.28	0.346
TBDF	-	-	-	-	-	-
PeBDFs	-	-	-	-	-	-
HxBDF	-	-	-	-	-	-
HpBDF	-	-	6.48	5.99	6.24	0.347
OBDF	-	-	-	-	-	-
TBDD	-	-	-	-	-	-
PeBDD	-	-	-	-	-	-
HxBDDs	-	-	-	-	-	-
HpBDD	-	-	-	-	-	-
OBDD	-	-	-	-	-	-
TriBDE	4.20	4.31	4.18	3.76	4.11	0.240
TetraBDE	4.36	4.59	4.52	4.06	4.38	0.235
PentaBDEs	4.57	4.71	4.40	4.17	4.46	0.233
HexaBDEs	5.03	4.95	5.49	4.62	5.02	0.359
HeptaBDE	6.18	5.88	6.68	5.46	6.05	0.515
OctaBDEs	6.00	5.93	6.01	5.69	5.91	0.147
NonaBDEs	5.75	5.63	5.39	5.33	5.53	0.200
DecaBDE	6.13	5.80	5.10	5.17	5.55	0.498
TetraCBs	4.45	4.77	4.33	3.91	4.37	0.359
PentaCBs	4.42	5.14	4.66	4.20	4.61	0.401
HexaCBs	4.83	5.55	5.14	4.75	5.07	0.362
HeptaCB	5.10	5.66	5.29	4.98	5.26	0.299
DiBB	4.44	-	-	-	4.44	-
TetraBB	-	5.03	-	-	5.03	-
HexaBB	-	-	-	-	-	-
HeptaBB	-	-	-	-	-	-
OctaBB	-	-	-	-	-	-

"-": There is N.D. data for POP homologues in MPs, POP homologues in SPM, or dissolved POP homologues in seawater, so enrichment factors cannot be calculated.

Table S49. The data on logarithm of EF_{MP-T} of POP homologues in the floating MPs

	AP1	AP2	KH1	KH2	Mean	SD
TCDF	6.57	-	6.40	-	6.48	0.124
PeCDFs	5.97	6.32	6.11	-	6.13	0.179
HxCDFs	-	6.17	6.28	-	6.23	0.0797
HpCDFs	6.01	6.54	6.09	6.39	6.26	0.251
OCDF	5.40	5.89	5.85	6.67	5.95	0.529
TCDD	-	-	-	-	-	-
PeCDD	6.65	-	6.30	-	6.48	0.243
HxCDDs	-	-	-	-	-	-
HpCDD	5.95	6.22	6.05	-	6.08	0.136
OCDD	5.17	5.74	5.67	6.21	5.70	0.427
TBDF	-	-	-	-	-	-
PeBDFs	-	-	-	-	-	-
HxBDF	-	-	-	-	-	-
HpBDF	-	-	6.82	-	6.82	-
OBDF	-	-	-	-	-	-
TBDD	-	-	-	-	-	-
PeBDD	-	-	-	-	-	-
HxBDDs	-	-	-	-	-	-
HpBDD	-	-	-	-	-	-
OBDD	-	-	-	-	-	-
TriBDE	5.73	5.89	5.86	5.56	5.76	0.152
TetraBDE	5.08	5.53	5.61	4.84	5.26	0.369
PentaBDEs	4.95	5.74	5.67	5.20	5.39	0.379
HexaBDEs	6.43	6.57	6.73	5.42	6.29	0.591
HeptaBDE	7.61	7.60	7.46	6.38	7.27	0.593
OctaBDEs	7.15	7.65	5.69	6.44	6.73	0.855
NonaBDEs	7.07	7.40	5.43	6.04	6.48	0.909
DecaBDE	7.06	7.07	5.33	6.07	6.38	0.844
TetraCBs	7.17	6.61	5.67	6.07	6.38	0.653
PentaCBs	6.50	6.42	5.81	5.84	6.14	0.371
HexaCBs	6.73	6.74	5.97	5.99	6.36	0.437
HeptaCB	6.77	6.91	5.91	6.31	6.47	0.456
DiBB	-	-	-	-	-	-
TetraBB	7.90	6.58	-	-	7.24	0.933
HexaBB	-	-	-	-	-	-
HeptaBB	-	-	-	-	-	-
OctaBB	-	-	-	-	-	-

"-": There is N.D. data for POP homologues in MPs, POP homologues in SPM, or dissolved POP homologues in seawater, so enrichment factors cannot be calculated.

S8. The estimated POPs and the estimated enrichment factor for POPs in organic matter of SPM

A simple calculation with the content of POPs in SPM and two measurements of volatile solids was conducted to consider the contribution of organic matter in SPM to the enrichment factor for POPs in organic matter of SPM ($EF_{Organic}$). The assumptions for the calculations are as follows:

- The results of two measurements of volatile solids represent the content of organic matter in SPM.
- The inorganic matter in SPM does not absorb POPs, and only the organic matter in SPM absorbs POPs.

The average of the two measurements of volatile solids in Table S40 is 25.4%, representing the content of organic matter. The concentration of POPs in SPM is divided by the content of organic matter (25.4%) to represent the concentration of POPs in the organic matter. The estimated results of POPs in the organic matter of SPM are presented in Tables S50–54. The estimated $EF_{Organic}$ is shown in Table S55. These results are very rough estimation, and further experimental data are needed to accurately assess the content of POPs in organic matter extracted from SPM.

Table S50. The estimated PCDD/Fs in organic matter of SPM in seawater

Concentration (unit: $pg \cdot g^{-1}$)	AP1	AP2	KH1	KH2
2,3,7,8-TeCDF	N.D.	N.D.	N.D.	1.07
1,2,3,7,8-PeCDF	1.96	1.34	1.17	1.70
2,3,4,7,8-PeCDF	2.79	1.57	1.59	1.65
1,2,3,4,7,8-HxCDF	N.D.	0.801	2.13	1.80
1,2,3,6,7,8-HxCDF	1.96	1.07	1.47	1.54
2,3,4,6,7,8-HxCDF	2.91	0.903	1.52	1.96
1,2,3,7,8,9-HxCDF	N.D.	N.D.	N.D.	N.D.
1,2,3,4,6,7,8-HpCDF	8.76	2.48	7.06	6.31
1,2,3,4,7,8,9-HpCDF	2.17	0.351	1.02	0.931
OCDF	22.1	5.05	24.9	11.2
2,3,7,8-TeCDD	N.D.	N.D.	N.D.	N.D.
1,2,3,7,8-PeCDD	1.73	0.385	0.588	0.714
1,2,3,4,7,8-HxCDD	N.D.	N.D.	N.D.	N.D.
1,2,3,6,7,8-HxCDD	1.14	0.489	N.D.	0.540
1,2,3,7,8,9-HxCDD	N.D.	0.446	1.23	0.821
1,2,3,4,6,7,8-HpCDD	18.5	3.25	8.88	4.85
OCDD	88.8	24.1	56.0	49.7
Σ17PCDD/Fs	153	42.2	107	84.8

Table S51. The estimated PBDD/Fs in organic matter of SPM in seawater

Concentration (unit: pg·g^{-1})	AP1	AP2	KH1	KH2
2,3,7,8-TeBDF	N.D.	N.D.	N.D.	N.D.
1,2,3,7,8-PeBDF	N.D.	N.D.	N.D.	N.D.
2,3,4,7,8-PeBDF	N.D.	N.D.	4.46	N.D.
1,2,3,4,7,8-HxBDF	N.D.	N.D.	41.4	8.28
1,2,3,4,6,7,8-HpBDF	60.0	21.7	381	64.3
OctBDF	82.8	27.9	371	107
2,3,7,8,-TeBDD	N.D.	N.D.	N.D.	N.D.
1,2,3,7,8-PeBDD	N.D.	N.D.	N.D.	N.D.
1,2,3,4/6,7,8-HxBDD	N.D.	N.D.	N.D.	N.D.
1,2,3,7,8,9-HxBDD	N.D.	N.D.	N.D.	N.D.
1,2,3,4,6,7,8-HpBDD	N.D.	N.D.	N.D.	N.D.
OctBDD	N.D.	N.D.	N.D.	N.D.
Σ12PBDD/Fs	143	49.7	797	179

Table S52. The estimated PBDEs in organic matter of SPM in seawater

Concentration (unit: pg·g^{-1})	AP1	AP2	KH1	KH2
BDE #28(3Br)	14.4	17.6	14.0	5.21
BDE #47(4Br)	836	738	536	243
BDE #100(5Br)	207	130	82.1	45.0
BDE #99(5Br)	990	568	291	174
BDE #154(6Br)	159	66.7	236	36.8
BDE #153(6Br)	177	79.7	422	47.3
BDE #183(7Br)	378	187	1180	226
BDE #197(8Br)	201	121	2990	552
BDE #203(8Br)	342	269	7100	1360
BDE #196(8Br)	382	215	6630	1310
BDE #208(9Br)	442	271	7690	2520
BDE #207(9Br)	1140	493	23500	6550
BDE #206(9Br)	1240	568	23100	5960
BDE #209(10Br)	20900	7420	109000	38400
Σ14BDE	27500	11200	183000	57600

Table S53. The estimated dioxin-like PCBs in organic matter of SPM in seawater

Concentration (unit: $pg \cdot g^{-1}$)	AP1	AP2	KH1	KH2
PCB#77(4CL)	17.4	26.8	21.9	7.97
PCB#81(4CL)	0.809	1.19	N.D.	N.D.
PCB#105(5CL)	43.4	145	75.3	25.2
PCB#114(5CL)	1.44	6.67	4.50	1.01
PCB#118(5CL)	136	576	218	77.3
PCB#123(5CL)	4.42	31.1	3.94	1.76
PCB#126(5CL)	1.45	3.65	3.24	1.29
PCB#156(6CL)	28.0	170	49.7	20.7
PCB#157(6CL)	4.77	20.2	8.84	3.47
PCB#167(6CL)	20.9	84.8	34.8	13.8
PCB#169(6CL)	ND	0.473	N.D.	N.D.
PCB#189(7CL)	4.50	25.2	8.09	4.54
Σ12PCB	264	1090	430	157

Table S54. The estimated PBBs in organic matter of SPM in seawater

Concentration (unit: $pg \cdot g^{-1}$)	AP1	AP2	KH1	KH2
PBB#15(2Br)	5.84	N.D.	N.D.	N.D.
PBB#52(4Br)	N.D.	1.08	N.D.	N.D.
PBB#153(6Br)	N.D.	9.23	N.D.	N.D.
PBB#180(7Br)	N.D.	N.D.	N.D.	N.D.
PBB#194(8Br)	N.D.	N.D.	N.D.	N.D.
Σ5PBB	5.84	10.3	N.D.	N.D.

Table S55. The estimated logarithm of $EF_{Organic}$ of POPs and TEQ-POPs

	AP1	AP2	KH1	KH2	Mean	SD
PCDD/Fs	5.70	5.65	6.14	6.17	5.92	0.278
PBDD/Fs	-	-	7.40	7.03	7.22	0.262
PBDEs	6.06	5.94	5.81	5.79	5.90	0.126
PCBs	5.09	5.80	5.31	4.87	5.27	0.398
PBBs	5.01	5.53	-	-	5.27	0.368
TEQ-PCDD/Fs	6.15	6.24	5.77	6.09	6.06	0.205
TEQ-PBDD/Fs	-	-	7.47	6.96	7.22	0.361
TEQ-PCBs	5.33	6.00	5.58	5.09	5.50	0.389

References

(1) Nguyen, D. D., Tsai, C. L., Hsu, Y. C., Chen, Y. W., Weng, Y. M., & Chang, M. B. (2017). PCDD/Fs and dl-PCBs concentrations in water samples of Taiwan. Chemosphere, 173, 603-611.

(2) Thuan, N. T., Tsai, C. L., Weng, Y. M., Lee, T. Y., & Chang, M. B. (2011). Analysis of polychlorinated dibenzo-p-dioxins and furans in various aqueous samples in Taiwan. Chemosphere, 83(6), 760-766.

(3) Chen, C. F., Ju, Y. R., Lim, Y. C., Wang, M. H., Albarico, F. P. J. B., Chen, C. W., & Dong, C. D. (2022). Occurrence and ecological risks of PAHs in the dissolved and particulate phases of coastal surface water of Taiwan. Regional Studies in Marine Science, 54, 102503.

(4) Chen, C. F., Lim, Y. C., Ju, Y. R., Albarico, F. P. J. B., Cheng, J. W., Chen, C. W., & Dong, C. D. (2022). Method development for low-concentration PAHs analysis in seawater to evaluate the impact of ship scrubber washwater effluents. Water, 14(3), 287.

(5) Cormier, B., Gambardella, C., Tato, T., Perdriat, Q., Costa, E., Veclin, C., Le Bihanic, F., Grassl, B., Dubocq, F., Kärrman, A., Van Arkel, K., Lemoine, S., Lagarde, F., Morin, B., Garaventa, F., Faimali, M., Cousin, X., Bégout, M.-L., Beiras, R., Cachot, J., 2021. Chemicals sorbed to environmental microplastics are toxic to early life stages of aquatic organisms. Ecotoxicology and Environmental Safety 208, 111665.

(6) Llorca, M., Vega-Herrera, A., Schirinzi, G., Savva, K., Abad, E., Farré, M., 2021. Screening of suspected micro(nano)plastics in the Ebro Delta (Mediterranean Sea). Journal of Hazardous Materials 404, 124022.

(7) Sun, B., Liu, J., Zhang, Y.-Q., Leungb, K.M.Y., Zeng, E.Y., 2021. Leaching of polybrominated diphenyl ethers from microplastics in fish oil: Kinetics and bioaccumulation. Journal of Hazardous Materials 406, 124726.

(8) Aminot, Y., Lanctôt, C., Bednarz, V., Robson, W.J., Taylor, A., Ferrier-Pagès, C., Metian, M., Tolosa, I., 2020. Leaching of flame-retardants from polystyrene debris: Bioaccumulation and potential effects on coral. Marine Pollution Bulletin 151, 110862.

(9) Tanaka, K., van Franeker, J.A., Deguchi, T., Takada, H., 2019. Piece-by-piece analysis of additives and manufacturing byproducts in plastics ingested by seabirds: Implication for risk of exposure to seabirds. Marine Pollution Bulletin 145, 36-41.

(10) Tian, Z., Kim, S.-K., Hyun, J.-H., 2020. Environmental Distribution of Styrene Oligomers (SOs) Coupled with Their Source Characteristics: Tracing the Origin of SOs in the Environment. Journal of Hazardous Materials 398, 122968.

(11) Fikarová, K., Cocovi-Solberg, D.J., Rosende, M., Horstkotte, B., Sklenářová, H., Miró, M., 2019. A flow-based platform hyphenated to on-line liquid chromatography for automatic leaching tests of chemical additives from microplastics into seawater. Journal of Chromatography A 1602, 160-167.

(12) Sun, B., Hu, Y., Cheng, H., Tao, S., 2019. Releases of brominated flame retardants (BFRs) from microplastics in aqueous medium: Kinetics and molecular-size dependence of diffusion. Water Research 151, 215-225.

(13) Suhrhoff, T.J., Scholz-Böttcher, B.M., 2016. Qualitative impact of salinity, UV radiation and turbulence on leaching of organic plastic additives from four common plastics — A lab experiment. Marine Pollution Bulletin 102, 84-94.

(14) Hirai, H., Takada, H., Ogata, Y., Yamashita, R., Mizukawa, K., Saha, M., Kwan, C., Moore, C., Gray, H., Laursen, D., Zettler, E.R., Farrington, J.W., Reddy, C.M., Peacock, E.E., Ward, M.W., 2011. Organic micropollutants in marine plastics debris from the open ocean and remote and urban beaches. Marine Pollution Bulletin 62, 1683-1692.

(15) Syberg, K., Knudsen, C.M.H., Tairova, Z., Khan, F.R., Shashoua, Y., Geertz, T., Pedersen, H.B., Sick, C., Mortensen, J., Strand, J., Palmqvist, A., 2020. Sorption of PCBs to environmental plastic pollution in the North Atlantic Ocean: Importance of size and polymer type. Case Studies in Chemical and Environmental Engineering 2, 100062.

(16) Pflieger, M., Makorič, P., Kovač Viršek, M., Koren, Š., 2017. Extraction of Organochlorine Pesticides from Plastic Pellets and Plastic Type Analysis. J Vis Exp.

(17) Van, A., Rochman, C.M., Flores, E.M., Hill, K.L., Vargas, E., Vargas, S.A., Hoh, E., 2012. Persistent organic pollutants in plastic marine debris found on beaches in San Diego, California. Chemosphere 86, 258-263.

(18) Heskett, M., Takada, H., Yamashita, R., Yuyama, M., Ito, M., Geok, Y.B., Ogata, Y., Kwan, C., Heckhausen, A., Taylor, H., Powell, T., Morishige, C., Young, D., Patterson, H., Robertson, B., Bailey, E., Mermoz, J., 2012. Measurement of persistent organic pollutants (POPs) in plastic resin pellets from remote islands: Toward establishment of background concentrations for International Pellet Watch. Marine Pollution Bulletin 64, 445-448.

(19) Wang, L. C., Wang, Y. F., Hsi, H. C., & Chang-Chien, G. P. (2010). Characterizing the emissions of polybrominated diphenyl ethers (PBDEs) and polybrominated dibenzo-p-dioxins and dibenzofurans (PBDD/Fs) from metallurgical processes. Environmental science & technology, 44(4), 1240-1246.

(20) Chang, Y. C., Lee, W. J., Yang, H. H., Wang, L. C., Lu, J. H., Tsai, Y. I., Cheng, M. T., Young, L. H., Chiang, C. J., 2014. Reducing emissions of persistent organic

pollutants from a diesel engine by fueling with water-containing butanol diesel blends. Environmental science & technology, 48(10), 6010-6018.
(21) Chao, H. R., Lin, D. Y., Chen, K. Y., Gou, Y. Y., Chiou, T. H., Lee, W. J., ... & Wang, L. C. (2014). Atmospheric concentrations of persistent organic pollutants over the Pacific Ocean near southern Taiwan and the northern Philippines. Science of the total environment, 491, 51-59.
(22) Mwangi, J. K., Lee, W. J., Wang, L. C., Sung, P. J., Fang, L. S., Lee, Y. Y., & Chang-Chien, G. P. (2016). Persistent organic pollutants in the Antarctic coastal environment and their bioaccumulation in penguins. Environmental Pollution, 216, 924-934.
(23) Wang, L. C., Chun-Te Lin, J., Dong, C. D., Chen, C. W., & Liu, T. K. (2021). The sorption of persistent organic pollutants in microplastics from the coastal environment. Journal of Hazardous Materials, 420, 126658.
(24) Ye, J. A., Wang, L. C., & Liu, T. K. (2024). Accumulation of persistent organic pollutants by MPs in coastal wastewater treatment plants. Marine Pollution Bulletin, 207, 116922.
(25) Pouch, A., Zaborska, A., & Pazdro, K. (2021). Levels of dioxins and dioxin-like polychlorinated biphenyls in seawater from the Hornsund fjord (SW Svalbard). Marine Pollution Bulletin, 162, 111917.
(26) Liu, H., Tang, S., Zheng, X., Zhu, Y., Ma, Z., Liu, C., Hecker, M., Saunders, D. M. V., Giesy, J. P., Zhang, X., & Yu, H. (2015). Bioaccumulation, biotransformation, and toxicity of BDE-47, 6-OH-BDE-47, and 6-MeO-BDE-47 in early life-stages of zebrafish (Danio rerio). Environmental Science & Technology, 49(3), 1823-1833.
(27) Ge, W., Mou, Y., Chai, C., Zhang, Y., Wang, J., Ju, T., Jiang, T., & Xia, B. (2018). Polybrominated diphenyl ethers in the dissolved and suspended phases of seawater from Sanggou Bay, east China. Chemosphere, 203, 253-262.
(28) Liu, Y., Li, X., Zhang, S., & Ren, M. (2008). Polychlorinated dibenzo-p-dioxins and dibenzofurans (PCDD/Fs) in water and suspended particulate matter from the Xijiang River, China. Journal of hazardous materials, 152(1), 40-47.
(29) Moon, H. B., Choi, H. G., Lee, P. Y., & Ok, G. (2008). Congener-specific characterization and sources of polychlorinated dibenzo-p-dioxins, dibenzofurans and dioxin-like polychlorinated biphenyls in marine sediments from industrialized bays of Korea. Environmental Toxicology and Chemistry, 27(2), 323-333.
(30) van den Berg, M., Denison, M. S., Birnbaum, L. S., DeVito, M. J., Fiedler, H., Falandysz, J., Rose, M., Schrenk, D., Safe, S., Tohyama, C., Tritscher, A., Tysklind, M., & Peterson, R. E. (2013). Polybrominated dibenzo-p-dioxins, dibenzofurans, and biphenyls: inclusion in the toxicity equivalency factor concept

for dioxin-like compounds. Toxicological Sciences, 133(2), 197-208.
(31) Wang, F., Wong, C. S., Chen, D., Lu, X., Wang, F., & Zeng, E. Y. (2018). Interaction of toxic chemicals with microplastics: a critical review. Water research, 139, 208-219.
(32) Bjurlid, F., Roos, A., Jogsten, I. E., & Hagberg, J. (2018). Temporal trends of PBDD/Fs, PCDD/Fs, PBDEs and PCBs in ringed seals from the Baltic Sea (Pusa hispida botnica) between 1974 and 2015. Science of the Total Environment, 616, 1374-1383.
(33) Zacs, D., Rjabova, J., Fernandes, A., & Bartkevics, V. (2016). Brominated, chlorinated and mixed brominated/chlorinated persistent organic pollutants in European eels (Anquilla anquilla) from Latvian lakes. Food Additives & Contaminants: Part A, 33(3), 460-472.
(34) Falandysz, J., Smith, F., & Fernandes, A. R. (2020). Polybrominated dibenzo-p-dioxins (PBDDs) and–dibenzofurans (PBDFs) in cod (Gadus morhua) liver-derived products from 1972 to 2017. Science of The Total Environment, 722, 137840.
(35) Goto, A., Tue, N. M., Someya, M., Isobe, T., Takahashi, S., Tanabe, S., & Kunisue, T. (2017). Spatio-temporal trends of polybrominated dibenzo-p-dioxins and dibenzofurans in archived sediments from Tokyo Bay, Japan. Science of the Total Environment, 599, 340-347.
(36) Xu, P., Ge, W., Chai, C., Zhang, Y., Jiang, T., & Xia, B. (2019). Sorption of polybrominated diphenyl ethers by microplastics. Marine pollution bulletin, 145, 260-269.
(37) Zhu, X., Beiyuan, J., Lau, A. Y., Chen, S. S., Tsang, D. C., Graham, N. J., Lin, D., Sun, J., Pan, Y., Yang, X., & Li, X. D. (2018). Sorption, mobility, and bioavailability of PBDEs in the agricultural soils: Roles of co-existing metals, dissolved organic matter, and fertilizers. Science of the Total Environment, 619, 1153-1162.
(38) Wang, L. C., Chun-Te Lin, J., Dong, C. D., Chen, C. W., & Liu, T. K. (2021). The sorption of persistent organic pollutants in microplastics from the coastal environment. Journal of Hazardous Materials, 420, 126658.
(39) Jiang, J. J., Lee, C. L., Fang, M. D., Ko, F. C., & Baker, J. E. (2011). Polybrominated diphenyl ethers and polychlorinated biphenyls in sediments of southwest Taiwan: regional characteristics and potential sources. Marine pollution bulletin, 62(4), 815-823.
(40) Cheng, J. O., Tseng, P. H., Chou, P. H., Hsieh, C. Y., & Ko, F. C. (2021). Revisiting of persistent organic pollution occurrence and distribution in the surface sediment along western Taiwan coast. Marine Pollution Bulletin, 173, 113118.

(41) von der Recke, R., & Vetter, W. (2008). Congener pattern of hexabromobiphenyls in marine biota from different proveniences. Science of the total environment, 393(2-3), 358-366.
(42) Gieroń, J., Grochowalski, A., & Chrząszcz, R. (2010). PBB levels in fish from the Baltic and North seas and in selected food products from Poland. Chemosphere, 78(10), 1272-1278.
(43) U.S. EPA. Exposure and Human Health Reassessment of 2,3,7,8-Tetrachlorodibenzo-P-Dioxin (Tcdd) and Related Compounds National Academy Sciences (External Review Draft) (2004). U.S. Environmental Protection Agency, Washington, D.C., EPA/600/P-00/001Cb, 2004.
(44) Fernandez-Gonzalez, R., Yebra-Pimentel, I., Martinez-Carballo, E., & Simal-Gandara, J. (2015). A critical review about human exposure to polychlorinated dibenzo-p-dioxins (PCDDs), polychlorinated dibenzofurans (PCDFs) and polychlorinated biphenyls (PCBs) through foods. Critical reviews in food science and nutrition, 55(11), 1590-1617.
(45) Geyer, H. J., Rimkus, G. G., Scheunert, I., Kaune, A., Schramm, K. W., Kettrup, A., Zeeman, M., Muir, D. C. G., Hansen, L. G., & Mackay, D. (2000). Bioaccumulation and occurrence of endocrine-disrupting chemicals (EDCs), persistent organic pollutants (POPs), and other organic compounds in fish and other organisms including humans. In Bioaccumulation–New Aspects and Developments (pp. 1-166). Springer, Berlin, Heidelberg.
(46) Chen, J., Quan, X., Yazhi, Z., Yan, Y., & Yang, F. (2001). Quantitative structure–property relationship studies on n-octanol/water partitioning coefficients of PCDD/Fs. Chemosphere, 44(6), 1369-1374.
(47) Puzyn, T., Suzuki, N., & Haranczyk, M. (2008). How do the partitioning properties of polyhalogenated POPs change when chlorine is replaced with bromine?. Environmental science & technology, 42(14), 5189-5195.
(48) Yue, C., & Li, L. Y. (2013). Filling the gap: estimating physicochemical properties of the full array of polybrominated diphenyl ethers (PBDEs). Environmental pollution, 180, 312-323.
(49) EFSA Panel on Contaminants in the Food Chain (CONTAM). (2011). Scientific opinion on polybrominated diphenyl ethers (PBDEs) in food. EFSA journal, 9(5), 2156.
(50) Braekevelt, E., Tittlemier, S. A., & Tomy, G. T. (2003). Direct measurement of octanol–water partition coefficients of some environmentally relevant brominated diphenyl ether congeners. Chemosphere, 51(7), 563-567.
(51) ATSDR (Agency for Toxic Substances and Disease Registry), Public Health Service, U.S. Department of Health and Human Services. (2004) Toxicological

profile for polybrominated biphenyls and polybrominated diphenyl ethers. ATSDR, Atlanta, GA. Available online at http://www.atsdr.cdc.gov/toxpro2.html

(52) Bao, L. J., You, J., & Zeng, E. Y. (2011). Sorption of PBDE in low-density polyethylene film: Implications for bioavailability of BDE-209. Environmental toxicology and chemistry, 30(8), 1731-1738.

(53) Tittlemier, S. A., Halldorson, T., Stern, G. A., & Tomy, G. T. (2002). Vapor pressures, aqueous solubilities, and Henry's law constants of some brominated flame retardants. Environmental Toxicology and Chemistry: An International Journal, 21(9), 1804-1810.

(54) Palm, A., Cousins, I. T., Mackay, D., Tysklind, M., Metcalfe, C., & Alaee, M. (2002). Assessing the environmental fate of chemicals of emerging concern: a case study of the polybrominated diphenyl ethers. Environmental Pollution, 117(2), 195-213.

(55) Kuramochi, H., Maeda, K., & Kawamoto, K. (2007). Physicochemical properties of selected polybrominated diphenyl ethers and extension of the UNIFAC model to brominated aromatic compounds. Chemosphere, 67(9), 1858-1865.

(56) Papa, E., Kovarich, S., & Gramatica, P. (2009). Development, validation and inspection of the applicability domain of QSPR models for physicochemical properties of polybrominated diphenyl ethers. QSAR & Combinatorial Science, 28(8), 790-796.

(57) Papa, E., Kovarich, S., & Gramatica, P. (2011). On the use of local and global QSPRs for the prediction of physico-chemical properties of polybrominated diphenyl ethers. Molecular Informatics, 30(2-3), 232-240.

(58) Kelly, B. C., Ikonomou, M. G., Blair, J. D., Morin, A. E., & Gobas, F. A. (2007). Food web specific biomagnification of persistent organic pollutants. science, 317(5835), 236-239.

(59) Rapaport, R. A., & Eisenreich, S. J. (1984). Chromatographic determination of octanol-water partition coefficients (Kow's) for 58 PCB polychlorinated biphenyl congeners. Environmental science & technology, 18(3), 163-170.

(60) EFSA Panel on Contaminants in the Food Chain (CONTAM). (2010). Scientific opinion on polybrominated biphenyls (PBBs) in food. EFSA Journal, 8(10), 1789.
